# Supplementary material for: Causal effect of children’s secondary education on parental health outcomes: findings from a natural experiment in Botswana
Source: BMJ Open. 2021 Jan 12;11(1):e043247. doi: 10.1136/bmjopen-2020-043247 (PMC7805356; doi:10.1136/bmjopen-2020-043247)
Supplement: Supplementary data [file bmjopen-2020-043247supp001.pdf]

Table S1. Alternative Hypotheses Possibly Affecting Post-1980 Birth Cohorts.

Alternative hypothesis	Policy (year) and description	Concern or not
Curriculum changes	Revised National Policy of Education (1994): increase the vocational orientation of academic subjects, increase the number of practical subjects, emphasize professional skills, relate the curriculum to the professional environment, and increase career guidance.	Not implemented nationally; gradual implementation over at least five years.
National ART program	National ART Program (2002): ART became available free of charge through public health services. Aimed to deliver care with a high level of clinical monitoring and a low tolerance of adverse events, as in high-resource settings.	Rolled out later and/or did not affect specific birth cohorts.
Home based care, Prevention of Mother to Child Transmission, HIV testing policies	Community Home Based Care (1995): ensure continuity of comprehensive care services and social support to AIDS patients.<br>Prevention of Mother to Child Transmission (1999): available in all public health facilities.<br>HIV testing (2000): introduction of voluntary counselling and testing centers.	Rolled out later and did not affect specific birth cohorts.
Abortion policy change	The Penal Code Amendment Act of 11 October 1991 (1991): abortion was de-criminalized within the first 16 weeks of pregnancy under certain circumstances.	Implemented earlier and did not affect specific birth cohorts.
Family planning changes	National Population Policy (1997): improve quality of life and standard of living of all people; through reduced population growth rate, low fertility, low morbidity and mortality, and a balanced population distribution.	Proposed almost two years after the 1996 reform (August 1997); unlikely to have affected specific birth cohorts.
Socio-economic reforms	National Development Plan Number (19911997): outline short to medium-term development initiatives for Botswana	Implemented gradually over many years.
Drought relief program	Botswana’s drought relief program (1982-1990): address the loss of livestock and malnutrition, particularly among children sufficient food.	Implemented gradually over many years.

Abbreviation: ART: anti-retroviral therapy
