# Supplementary material for: Causal effect of children’s secondary education on parental health outcomes: findings from a natural experiment in Botswana
Source: BMJ Open. 2021 Jan 12;11(1):e043247. doi: 10.1136/bmjopen-2020-043247 (PMC7805356; doi:10.1136/bmjopen-2020-043247)
Supplement: Supplementary data [file bmjopen-2020-043247supp013.pdf]

Supplementary webappendix

- Text S1
- Figures S1-S5
- Tables S1-S7

Ludwig JO, *et al. BMJ Open* 2021; 11:e043247. doi: 10.1136/bmjopen-2020-043247

Text S1. Study context and education policy reform

Botswana is a sparsely-populated, land-locked, middle-income country in southern Africa, with a rapidly growing economy which is highly dependent on diamond-mining. Botswana has among the world's highest HIV prevalence rates, and with the scale-up of HIV treatment, life expectancy at birth increased from 48.1 years to 61.9 years between 2001 and 2011 (World Health Organization 2020).

Botswana education system and policy reform

Botswana's public education system is based on the "7-3-2" system, including seven years of primary school, three years of junior secondary school, and two years of senior secondary school. In January 1996, the Government of Botswana shifted grade 10 from senior secondary school to junior secondary school, as part of implementing the country's Revised National Policy on Education (Government of Botswana 1994). Botswana's National Commission on Education had criticized the former "7-2-3" system for not adequately preparing students for the labor market. Botswana's public education system is strongly centralized so that the policy was implemented rapidly and universally. Moreover, the vast majority of the population attends public schools with less than 1% of secondary school students in private schools (Statistics Botswana 2013). More teachers were hired, and classrooms were created. Travel times were reduced, and the number of grade 10 seats increased dramatically since there were many more junior secondary than senior secondary schools during the pre-reform period. The schooling reform also raised the benefits of completing grade 10 because it was now required to obtain a "Junior Certificate".

The result of the reform was a very large increase in the number of students attending grade 10 from 1997 to 1998 when the first cohorts were affected (i.e., those starting junior secondary school in 1996). Previous studies have found that the reform led to a large increase in schooling attainment for affected cohorts (Borkum 2009; De Neve et al. 2015; Lindskog and Durevall 2019) (**Figure S1**). In addition to schooling attainment, the reform improved children's health outcomes (e.g., sexual and reproductive health) (Lindskog and Durevall 2019); economic outcomes (e.g., labor force participation) (Borkum 2009); skills; and health services utilization (e.g., HIV testing) (Bor and De Neve 2015). This natural experiment provides an opportunity to estimate the causal impact of schooling on parental outcomes, by comparing parents of birth cohorts exposed to the reform versus those unexposed. The 1996 policy change to an education system with three years of junior secondary school is currently still in operation.

Ludwig JO, *et al. BMJ Open* 2021; 11:e043247. doi: 10.1136/bmjopen-2020-043247

Alternative hypotheses possibly affecting post-1980 birth cohorts

First, we consulted widely with scholars and government officials in Botswana to understand how the education policy reform was implemented and competing events that may have differentially affected post-1980 cohorts. Key stakeholders were identified through snowball sampling, and included staff from the Ministry of Health (n=2), Statistics Botswana (Central Statistics Botswana) (n=3), University of Botswana's Department of Economics (n=1), University of Botswana's Department of Education (n=2), the Botswana Harvard Partnership (n=2), as well as a teacher who worked in Botswana during the time of the 1996 reform (n=1). Interview questions covered the implementation of the 1996 reform; institutional and historical context; and any competing events. We asked for other social reforms or population changes that might have happened around the time and potentially have affected children born after 1980.

Second, we conducted a desk review of both published and unpublished documents. These included the Revised National Policy of Education (1994), the Revised National Population Policy (1997), the Seventh National Development Plan (1991-1997), as well as research and review articles on Botswana's education system and curriculum. In **Table S1**, we list alternative policy hypotheses with a brief description of each policy and whether or not these could be a concern for our main identification strategy. Potential concerns included, for instance, the roll-out of socio-economic reforms, vaccine or disaster relief programs, and/or HIV treatment and prevention programs if these affected specific children's birth cohorts.

Ludwig JO, *et al. BMJ Open* 2021; 11:e043247. doi: 10.1136/bmjopen-2020-043247

Figure S1. Children's schooling by year of birth in Botswana

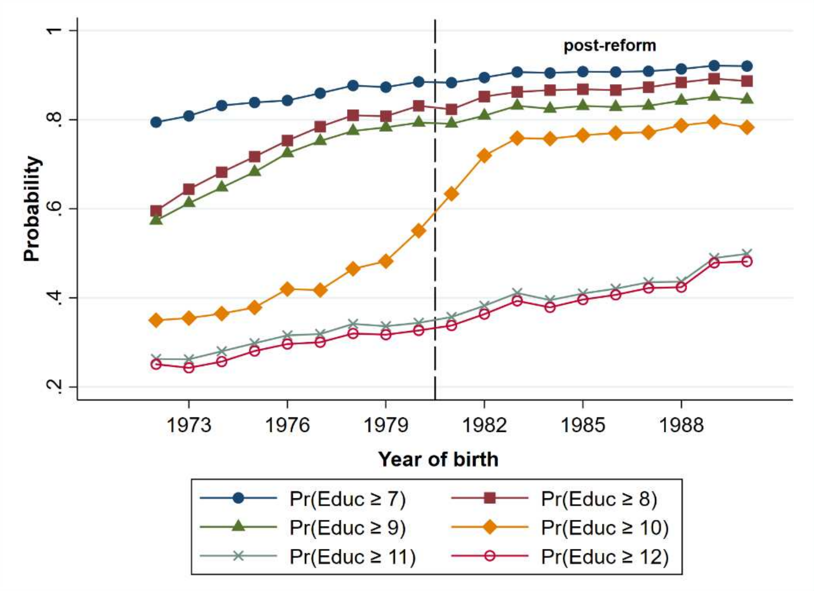

Notes: Figure replicates findings from De Neve et al., 2015 using data from the Botswana Census 2011 (*N*=108,092). The figure shows the probability that a child has attained at least X years of schooling by the time of the census. The sample includes survey respondents who were citizens of Botswana, born in Botswana and ages at least 18 years at the time of the census. Individuals born in 1981 or later (dashed line) would have entered junior secondary school in 1996 or later, and were thus classified as exposed to the education reform.

Figure S2. Assumptions underpinning causal inference

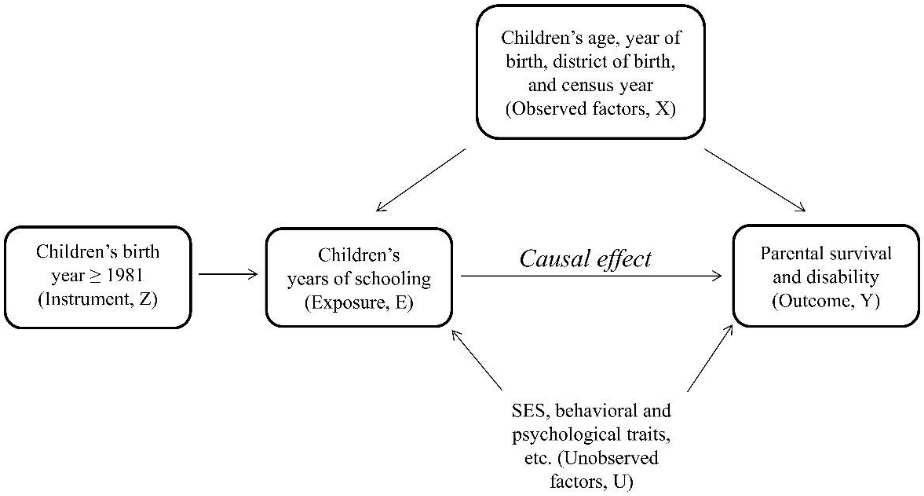

Notes: Directed acyclic graph illustrating the instrumental variable assumptions for causal interpretation. Conditional on *X*, *Z* is a valid instrument if *Z* causally affects *E*, *Z* is uncorrelated with *U*, and *Z* affects *Y* only through *E*. Under the assumption that *Z* only affects *E* in one direction, the instrumental variable estimators estimate a local average treatment effect.

Ludwig JO, *et al. BMJ Open* 2021; 11:e043247. doi: 10.1136/bmjopen-2020-043247

Figure S3. Cohort sizes

(a) Census 2001

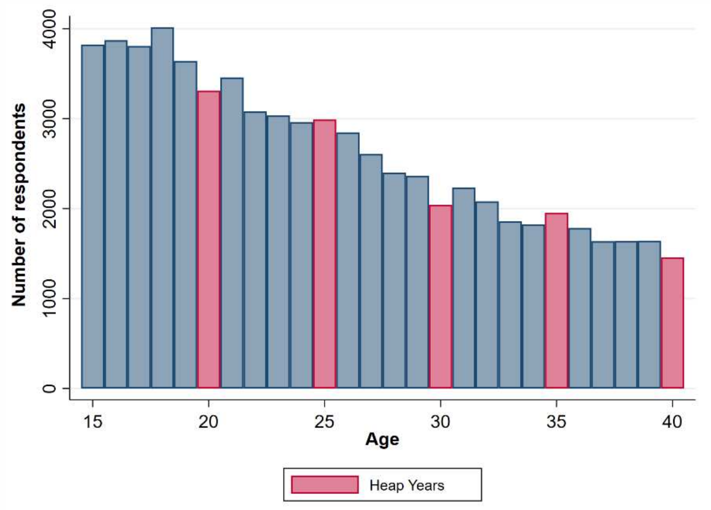

(b) Census 2011

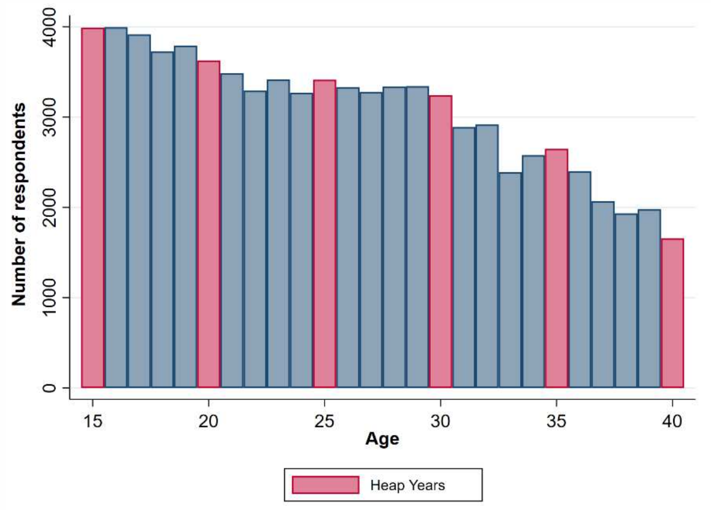

Notes: Figure shows the number of respondents by age (birth cohort), separately for each census wave. Respondents affected by the education policy reform were ages ≤ 20 (census 2001) or ≤ 30 (census 2011). Source: Botswana Census 2001 and 2011.

Figure S4. Parental survival and disability by children's year of birth

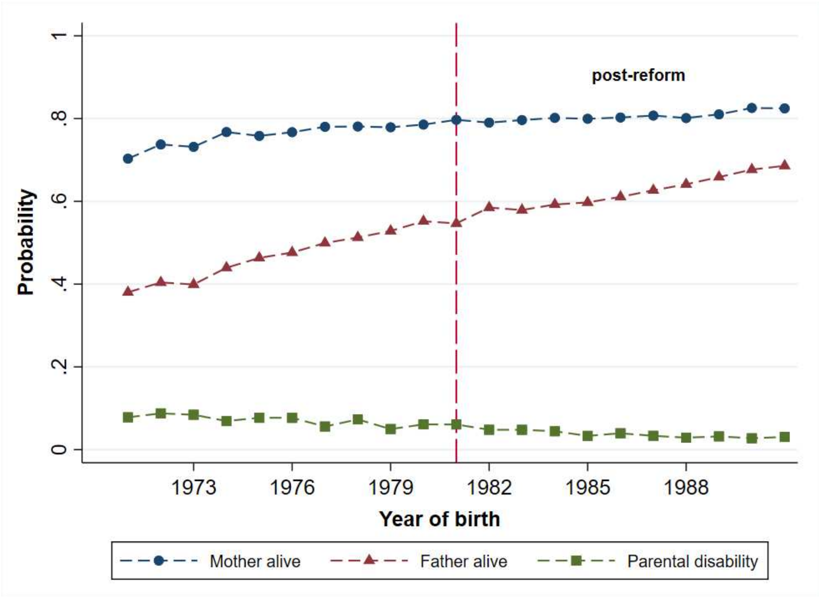

Notes: Figure shows survival of mothers (blue) and fathers (red) as well as parental disability (green) by children's birth cohort. The vertical dashed line indicates the timing of the policy reform. Parental disability was defined as any parental disability among the surviving mother and/or father. The sample for parental survival includes survey respondents who were citizens of Botswana and born in Botswana between 1971 and 1991; whereas the sample for disability additional includes only those children living with at least one parent. Source: Botswana Census 2011.

Figure S5. Bias component plots

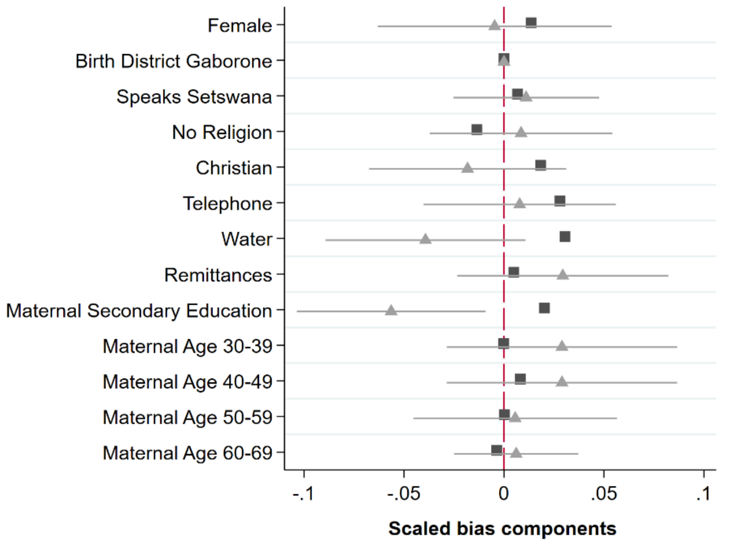

Notes: Bias component plots for the difference in children's sex, birth district, language, religion, household assets, household remittances, parental education, and parental age, by actual exposure (squares) and proposed instrument (triangles). The figures for the instrumental variable results account for the strength of the instrument as described in Jackson and Swanson (2015) and Davies (2017). All variables are binary. The horizontal lines indicate robust confidence intervals for years of schooling. Sample includes survey respondents who were citizens born in Botswana, at least 18 years old at the time of the census, born in or after 1975, and co-resided with at least one parent at the time of the census. Source: Botswana Census 2001 and 2011.

Table S1. Alternative Hypotheses Possibly Affecting Post-1980 Birth Cohorts.

**Alternative hypothesis**	**Policy (year) and description**	**Concern or not**
Curriculum changes	• Revised National Policy of Education (1994): increase the vocational orientation of academic subjects, increase the number of practical subjects, emphasize professional skills, relate the curriculum to the professional environment, and increase career guidance.	Not implemented nationally; gradual implementation over at least five years.
National ART program	• National ART Program (2002): ART became available free of charge through public health services. Aimed to deliver care with a high level of clinical monitoring and a low tolerance of adverse events, as in high-resource settings.	Rolled out later and/or did not affect specific birth cohorts.
Home based care, Prevention of Mother to Child Transmission, HIV testing policies	• Community Home Based Care (1995): ensure continuity of comprehensive care services and social support to AIDS patients.<br>• Prevention of Mother to Child Transmission (1999): available in all public health facilities.<br>• HIV testing (2000): introduction of voluntary counselling and testing centers.	Rolled out later and did not affect specific birth cohorts.
Abortion policy change	• The Penal Code Amendment Act of 11 October 1991 (1991): abortion was de-criminalized within the first 16 weeks of pregnancy under certain circumstances.	Implemented earlier and did not affect specific birth cohorts.
Family planning changes	• National Population Policy (1997): improve quality of life and standard of living of all people; through reduced population growth rate, low fertility, low morbidity and mortality, and a balanced population distribution.	Proposed almost two years after the 1996 reform (August 1997); unlikely to have affected specific birth cohorts.
Socio-economic reforms	• National Development Plan Number (1991-1997): outline short to medium-term development initiatives for Botswana	Implemented gradually over many years.
Drought relief program	• Botswana's drought relief program (1982-1990): address the loss of livestock and malnutrition, particularly among children sufficient food.	Implemented gradually over many years.

Abbreviation: ART: anti-retroviral therapy

Ludwig JO, *et al. BMJ Open* 2021; 11:e043247. doi: 10.1136/bmjopen-2020-043247

Table S2. Descriptive statistics: subset of children living with at least one parent (*N*=29,226)

Subsample: children co-residing with ≥ 1 parent	**Pre-reform cohorts (n = 9,467)**		**Post-reform cohorts (n = 19,759)**	
	**Census 2001**	**Census 2011**	**Census 2001**	**Census 2011**
Children's characteristics				
Any parental disability, %	5.7	6.4	4.8	3.5
Age, mean (SD)	23.1 (1.7)	33.3 (1.7)	18.9 (0.8)	23.1 (3.7)
Years of schooling, mean (SD)	9.1 (3.3)	9.1 (3.6)	9.5 (2.8)	10.4 (3.1)
Has at least ten years of schooling, %	45.0	42.1	68.2	78.0
Labor force participation, %	58.2	78.2	32.4	59.8
Speaks Setswana at home, %	84.5	86.6	83.0	83.0
Christian, %	71.8	77.4	73.4	79.3
Household characteristics				
Number of family members in household, mean (SD)	8.0 (3.6)	7.7 (3.8)	7.6 (3.5)	7.1 (3.5)
Any deaths in household last year, %	7.6	5.1	6.9	4.0
Ownership of dwelling, %	90.0	94.3	87.0	86.3
Access to electricity, %	20.9	53.6	21.1	53.2
Access to piped water, %	14.6	21.1	16.1	25.1
Telephone availability, %	37.3	18.6	35.8	16.9
Cellular phone availability, %	-	95.0	-	95.1
Receives remittances, %	24.1	35.0	24.0	32.9

Sample includes survey respondents who were citizens of Botswana, born in Botswana, at least 18 years old at the time of the census, born in or after 1975, and who co-resided with at least one parent at the time of the census. The unit of analysis was children born either during the pre-reform period (prior to 1981) or post-reform period (in or after 1981). SD=Standard Deviation. Source: Botswana Census 2001 and 2011.

Ludwig JO, *et al. BMJ Open* 2021; 11:e043247. doi: 10.1136/bmjopen-2020-043247

Table S3. OLS and ITT results controlling for parental socio-demographic characteristics

Dependent variable	**Parental disability (1=yes, 0=no)**		
Subsample	**Daughters**	**Sons**	**Both sexes**
	Risk difference (95% CI)		
A: OLS model			
Schooling (years)	-0.1*	-0.1	-0.1**
	(-0.2, 0.0)	(-0.2, 0.0)	(-0.2, -0.0)
B: OLS model			
≥ 10 years of schooling (1=yes,	-0.5	-1.0**	-0.7**
	(-1.3, 0.3)	(-1.9, -0.1)	(-1.3, -0.2)
C: ITT model			
Reform indicator	-0.3	-1.1	-0.7
	(-1.8, 1.1)	(-2.7, 0.5)	(-1.8, 0.4)
Additional controls			
Maternal age (years)	✓	✓	✓
Maternal schooling (years)	✓	✓	✓
Observations	15,006	12,127	27,133

Notes: Panels A and B show regression results from 'conventional' multivariable OLS models controlling for single-year age indicators, a continuous trend in year of birth, district of birth, as well as maternal age and maternal educational attainment (years of schooling completed). Regressions for the subsample with both children's sexes additionally control for an indicator for children's sex and interactions of each covariate with children's sex. Our models are robust to period effects, which we controlled for implicitly by simultaneously adjusting for children's age and year of birth. Panel C shows regression results from an ITT model (OLS), in which exposure to the reform was defined as a binary indicator (1=year of birth > 1980; 0=otherwise). The sample includes survey respondents who were citizens born in Botswana, at least 18 years old at the time of the census, born in or after 1975, and co-resided with at least their mother at the time of the census. Binary outcomes were multiplied by 100 to facilitate the interpretation of coefficients and standard errors on a % point scale. 95% robust confidence intervals in parentheses. *** p<0.01, ** p<0.05, * p<0.1. Source: Botswana Census 2001 and 2011.

Table S4. ITT results: testing the robustness of our findings to alternative specifications in children's age, YOB, additional controls for heap year, slope change in YOB, birth cohort windows, and analytical sample (outcome: maternal survival)

Dependent variable: mother alive (1=yes, 0=no)<br>***Sample: both sexes***<br>***Model: ITT***	**(1)**	**(2)**	**(3)**	**(4)**	**(5)**	**(6)**	**(7)**	**(8)**	**(9)**	**(10)**	**(11)**	**(12)**
Coefficient on endogenous variable (child)												
Reform indicator	-0.2	-0.2	0.1	0.4	0.8	0.6	-0.1	-0.2	-0.6	-0.6	0.7	-0.0
	(0.6)	(1.0)	(0.5)	(0.4)	(0.7)	(0.6)	(0.4)	(0.6)	(1.0)	(0.6)	(0.9)	(1.2)
Basic covariates (child)												
i.Age##i.Sex	✓	✓	-	-	-	-	-	✓	✓	✓	✓	✓
c.YOB##i.Sex	✓	✓	✓	✓	✓	✓	✓	✓	✓	✓	✓	✓
i.CensusYear##i.Sex	✓	✓	✓	✓	✓	✓	✓	✓	✓	✓	✓	✓
i.BirthDistrictr##i.Sex	✓	✓	✓	✓	✓	✓	✓	✓	✓	✓	✓	✓
Additional controls (child)												
c.YOB2##i.Sex	-	✓	-	-	-	-	-	-	-	-	-	-
i.ThreeYearAgeGroup##i.Sex	-	-	✓	-	-	-	-	-	-	-	-	-
c.Age##i.Sex	-	-	-	✓	✓	✓	✓	-	-	-	-	-
c.Age2##i.Sex	-	-	-	✓	✓	✓	✓	-	-	-	-	-
c.Age3##i.Sex	-	-	-	✓	-	-	-	-	-	-	-	-
c.Age4##i.Sex	-	-	-	✓	-	-	-	-	-	-	-	-
i.HeapYear##i.Sex	-	-	-	-	-	-	-	✓	-	-	-	-
c.YOB#i.Reform_Indicator, c.YOB#i.Reform_Indicator#i.Sex	-	-	-	-	-	-	-	-	✓	-	-	-
Birth cohort windows (child)												
Narrower birth cohort window, 1978 ≤ YOB ≤ 1984	-	-	-	-	✓	-	-	-	-	-	-	-
Narrower birth cohort window, 1976 ≤ YOB ≤ 1986	-	-	-	-	-	✓	-	-	-	-	-	-
Earlier birth cohorts included, 1971 ≤ YOB ≤ 1993	-	-	-	-	-	-	✓	-	-	-	-	-
Subsample, children with ≥ 9 years of schooling	-	-	-	-	-	-	-	-	-	✓	-	-
Subsample, co-resides with parent and gap ≥ 5.5 years of schooling	-	-	-	-	-	-	-	-	-	-	✓	-
Subsample, co-resides with parent and paternal age ≥ 50 years	-	-	-	-	-	-	-	-	-	-	-	✓
Observations	89,721	89,721	89,721	89,721	41,942	59,866	106,774	89,721	89,721	71,608	14,547	9,691

Notes: Robustness checks for the intention-to-treat (ITT) regression results shown in column 4 in Table 3 in the main text (maternal survival). The sample in models 1–4 and models 8–12 includes survey respondents who were citizens born in Botswana, at least 18 years old at the time of the census, and born in or after 1975. Binary outcomes were multiplied by 100 to facilitate the interpretation of coefficients and standard errors on a % point scale. Robust standard errors in parentheses. *** p<0.01, ** p<0.05, * p<0.1. Source: Botswana Census 2001 and 2011. YOB=year of birth.

Table S5. ITT results: testing the robustness of our findings to alternative specifications in children's age, YOB, additional controls for heap year, slope change in YOB, birth cohort windows, and analytical sample (outcome: paternal survival)

Dependent variable: father alive (1=yes, 0=no)<br>***Sample: both sexes***<br>***Model: ITT***	**(1)**	**(2)**	**(3)**	**(4)**	**(5)**	**(6)**	**(7)**	**(8)**	**(9)**	**(10)**	**(11)**	**(12)**
Coefficient on endogenous variable (child)												
Reform indicator	0.5	1.3	0.4	0.7	0.5	0.1	-0.2	-0.2	1.0	0.0	0.7	-0.5
	(0.8)	(1.4)	(0.7)	(0.6)	(0.9)	(0.7)	(0.5)	(0.6)	(1.2)	(0.8)	(0.9)	(2.1)
Basic covariates (child)												
i.Age##i.Sex	✓	✓	-	-	-	-	-	✓	✓	✓	✓	✓
c.YOB##i.Sex	✓	✓	✓	✓	✓	✓	✓	✓	✓	✓	✓	✓
i.CensusYear##i.Sex	✓	✓	✓	✓	✓	✓	✓	✓	✓	✓	✓	✓
i.BirthDistrictr##i.Sex	✓	✓	✓	✓	✓	✓	✓	✓	✓	✓	✓	✓
Additional controls (child)												
c.YOB2##i.Sex	-	✓	-	-	-	-	-	-	-	-	-	-
i.ThreeYearAgeGroup##i.Sex	-	-	✓	-	-	-	-	-	-	-	-	-
c.Age##i.Sex	-	-	-	✓	✓	✓	✓	-	-	-	-	-
c.Age2##i.Sex	-	-	-	✓	✓	✓	✓	-	-	-	-	-
c.Age3##i.Sex	-	-	-	✓	-	-	-	-	-	-	-	-
c.Age4##i.Sex	-	-	-	✓	-	-	-	-	-	-	-	-
i.HeapYear##i.Sex	-	-	-	-	-	-	-	✓	-	-	-	-
c.YOB#i.Reform_Indicator, c.YOB#i.Reform_Indicator#i.Sex	-	-	-	-	-	-	-	-	✓	-	-	-
Birth cohort windows (child)												
Narrower birth cohort window, 1978 < YOB < 1984	-	-	-	-	✓	-	-	-	-	-	-	-
Narrower birth cohort window, 1976 < YOB < 1986	-	-	-	-	-	✓	-	-	-			
Earlier birth cohorts included, 1971 < YOB < 1993	-	-	-	-	-	-	✓	-	-	-	-	-
Subsample, children with > 9 years of schooling	-	-	-	-	-	-	-	-	-	✓	-	-
Subsample, co-resides with parent and gap > 5.5 years of schooling	-	-	-	-	-	-	-	-	-	-	✓	-
Subsample, co-resides with parent and maternal age > 50 years	-	-	-	-	-	-	-	-	-	-	-	✓
Observations	89,721	89,721	89,721	89,721	41,942	59,866	106,774	89,721	89,721	71,608	14,547	13,856

Notes: Robustness checks for the intention-to-treat (ITT) regression results shown in column 7 in Table 3 in the main text (paternal survival). The sample in models 1–4 and models 8–12 includes survey respondents who were citizens born in Botswana, at least 18 years old at the time of the census, and born in or after 1975. Binary outcomes were multiplied by 100 to facilitate the interpretation of coefficients and standard errors on a % point scale. Robust standard errors in parentheses. *** p<0.01, ** p<0.05, * p<0.1. Source: Botswana Census 2001 and 2011. YOB=year of birth.

Table S6. OLS and ITT results: stratified by parental age group

Dependent variable	**Parental disability (1=yes, 0=no)**		
Parental age group	**Age 50-59**	**Age 60-69**	**Age 70-79**
		Risk difference (95% CI)	
A: OLS model			
Schooling (years)	-0.0	-0.3***	-0.1
	(-0.2, 0.1)	(-0.5, -0.1)	(-0.6, 0.3)
B: OLS model			
≥ 10 years of schooling (1=yes, 0=no)	-0.5	-1.6**	-2.0
	(-1.3, 0.3)	(-3.0, -0.2)	(-4.9, 0.9)
C: ITT model			
Reform indicator	-0.6	-1.0	-2.1
	(-2.4, 1.3)	(-4.6, 2.5)	(-10.2, 6.1)
Probability dependent variable, pre-reform	5.3	8.2	13.9
Observations	12,447	6,329	2,262

Notes: Panels A and B show regression results from an OLS model controlling for single-year age indicators, a continuous trend in year of birth, district of birth, and the interaction of each covariate with children's sex. Our models are robust to period effects, which we controlled for implicitly by simultaneously adjusting for children's age and year of birth. Panel C shows regression results from an ITT model (OLS), in which exposure to the reform was defined as a binary indicator (1=year of birth > 1980; 0=otherwise). The sample includes citizens born in Botswana, at least 18 years old at the time of the census, and born in or after 1975. Binary outcomes were multiplied by 100 to facilitate the interpretation of coefficients and standard errors on a % point scale. 95% robust confidence intervals in parentheses. *** p<0.01, ** p<0.05, * p<0.1. Source: Botswana Census 2001 and 2011.

Ludwig JO, *et al. BMJ Open* 2021; 11:e043247. doi: 10.1136/bmjopen-2020-043247

Table S7. OLS and ITT results: logistic regression models.

Dependent variable	**Mother alive (1=yes, 0=no)**			**Father alive (1=yes, 0=no)**		
Subsample	**Daughters**	**Sons**	**Both sexes**	**Daughters**	**Sons**	**Both sexes**
	Odds ratios (95% CI)					
A: OLS model						
Schooling (years)	1.063***	1.048***	1.055***	1.035***	1.023***	1.028***
	(1.056, 1.071)	(1.041, 1.056)	(1.050, 1.060)	(1.029, 1.041)	(1.017, 1.029)	(1.024, 1.033)
B: OLS model						
$\geq$ 10 years of schooling (1=yes, 0=no)	1.402***	1.309***	1.356***	1.199***	1.091***	1.145***
	(1.328, 1.479)	(1.238, 1.384)	(1.304, 1.409)	(1.148, 1.252)	(1.044, 1.141)	(1.110, 1.181)
C: ITT model						
Reform indicator	0.988	1.054	1.018	1.094	1.052	1.074
	(0.776, 1.258)	(0.816, 1.362)	(0.854, 1.214)	(0.913, 1.311)	(0.869, 1.274)	(0.942, 1.225)
Observations	47,121	42,600	89,721	47,121	42,600	89,721

Notes: Logistic regression models controlling for single-year children's age indicators, a continuous trend in children's year of birth and year of birth squared, and children's district of birth. Regressions for the subsample with both sexes additionally control for an indicator for children's sex and interactions of each covariate with children's sex. Our models are robust to period effects, which we controlled for implicitly by simultaneously adjusting for children's age and year of birth. Panel C shows regression results from an intention-to-treat (ITT) model in which exposure to the reform was defined as a binary indicator (1=year of birth > 1980; 0=otherwise). The sample includes survey respondents who were citizens born in Botswana, at least 18 years old at the time of the census, and born in or after 1975. Robust 95% confidence intervals in parentheses. *** p<0.01, ** p<0.05, * p<0.1. Source: Botswana Census 2001 and 2011.

Ludwig JO, *et al. BMJ Open* 2021; 11:e043247. doi: 10.1136/bmjopen-2020-043247

References for supplementary webappendix

1. Bor, J.and J.-W. De Neve (2015). "A Social Vaccine? HIV Infection, Fertility, and the Non-Pecuniary Returns to Secondary Schooling in Botswana." Population Association of America Annual Meeting. San Diego [Available at: https://paa2015.princeton.edu/abstracts/151006].
2. Borkum, E. (2009). "Grade structure, educational attainment and labor market outcomes: Evidence from Botswana" (Job Market Paper). Columbia University.
3. Government of Botswana. (1991). National Development Plan - 7. Gaborone, Botswana: Botswana Government Printer.
4. Government of Botswana. (1994). The revised national policy on education. Gaborone, Botswana: Botswana Government Printer.
5. Government of Botswana. (1997). National Population Policy. Gaborone, Botswana: Botswana Government Printer.
6. De Neve J-W, Fink G, Subramanian SV, Moyo S, & Bor J (2015) Length of secondary schooling and risk of HIV infection in Botswana: evidence from a natural experiment. *The Lancet Global Health* 3(8):e470-e477.
7. Statistics Botswana (2013). "Education Report Draft from 2009/10 Botswana Core Welfare Indicators Survey." Gaborone, Botswana: Government of Botswana [Available at: http://www.statsbots.org.bw/].
8. World Health Organization (2020). "Global Health Observatory data repository." World Health Organization. Geneva, Switzerland [Available at: https://www.who.int/data/gho].
