# Supplementary material for: Causal effect of children’s secondary education on parental health outcomes: findings from a natural experiment in Botswana
Source: BMJ Open. 2021 Jan 12;11(1):e043247. doi: 10.1136/bmjopen-2020-043247 (PMC7805356; doi:10.1136/bmjopen-2020-043247)
Supplement: Supplementary data [file bmjopen-2020-043247supp008.pdf]

Figure S1. Children's schooling by year of birth in Botswana

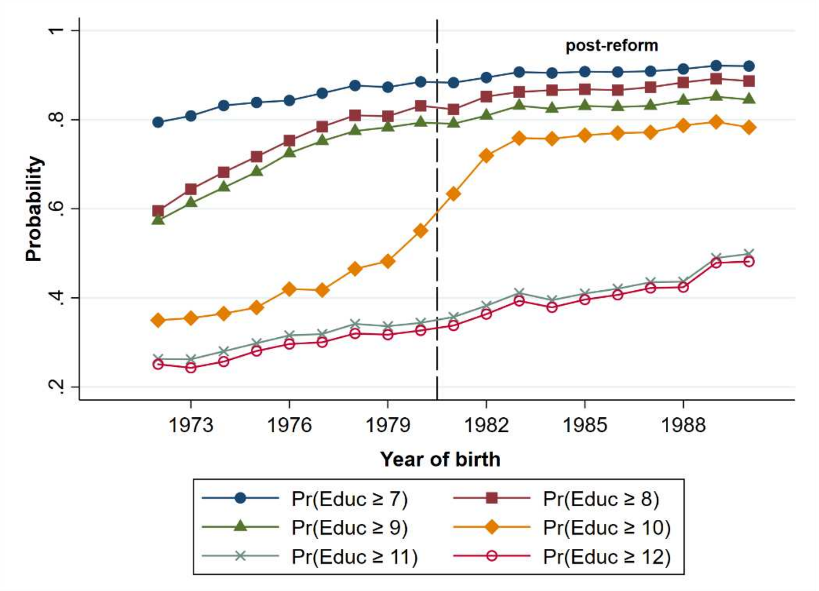

Notes: Figure replicates findings from De Neve et al., 2015 using data from the Botswana Census 2011 (*N*=108,092). The figure shows the probability that a child has attained at least X years of schooling by the time of the census. The sample includes survey respondents who were citizens of Botswana, born in Botswana and ages at least 18 years at the time of the census. Individuals born in 1981 or later (dashed line) would have entered junior secondary school in 1996 or later, and were thus classified as exposed to the education reform.
