# Supplementary material for: Causal effect of children’s secondary education on parental health outcomes: findings from a natural experiment in Botswana
Source: BMJ Open. 2021 Jan 12;11(1):e043247. doi: 10.1136/bmjopen-2020-043247 (PMC7805356; doi:10.1136/bmjopen-2020-043247)
Supplement: Supplementary data [file bmjopen-2020-043247supp009.pdf]

Figure S2. Assumptions underpinning causal inference

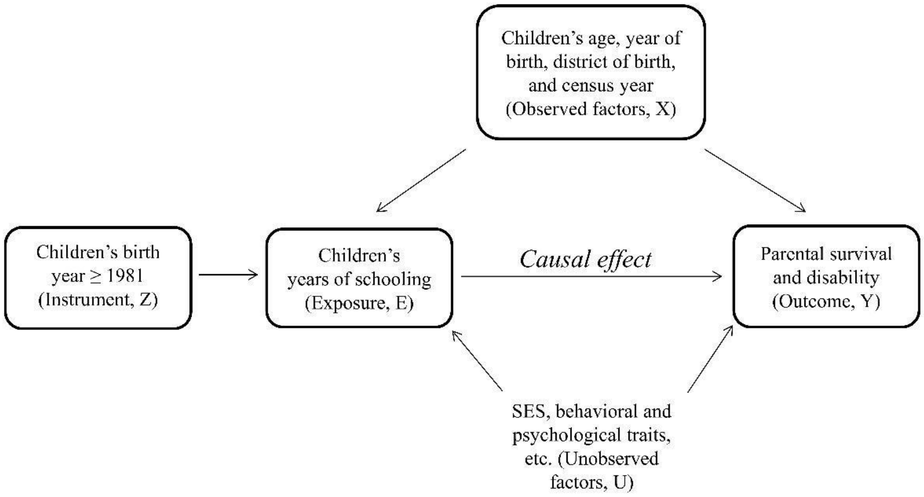

Notes: Directed acyclic graph illustrating the instrumental variable assumptions for causal interpretation. Conditional on X, Z is a valid instrument if Z causally affects E, Z is uncorrelated with U, and Z affects Y only through E. Under the assumption that Z only affects E in one direction, the instrumental variable estimators estimate a local average treatment effect.
