# Supplementary material for: Causal effect of children’s secondary education on parental health outcomes: findings from a natural experiment in Botswana
Source: BMJ Open. 2021 Jan 12;11(1):e043247. doi: 10.1136/bmjopen-2020-043247 (PMC7805356; doi:10.1136/bmjopen-2020-043247)
Supplement: Supplementary data [file bmjopen-2020-043247supp010.pdf]

Figure S3. Cohort sizes

(a) Census 2001

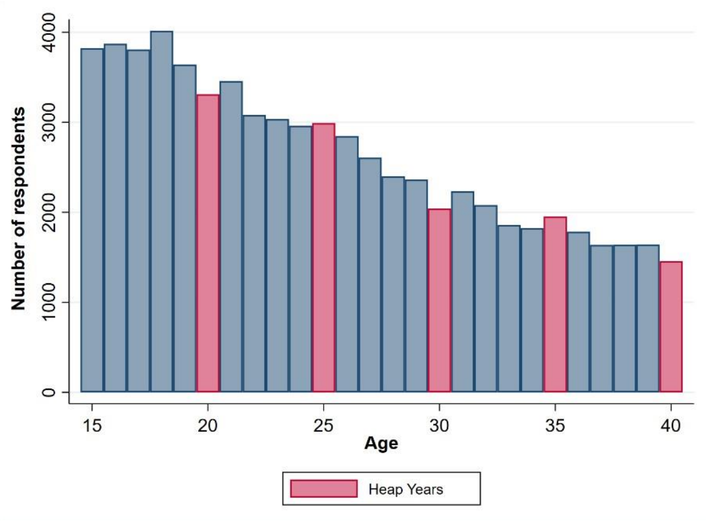

(b) Census 2011

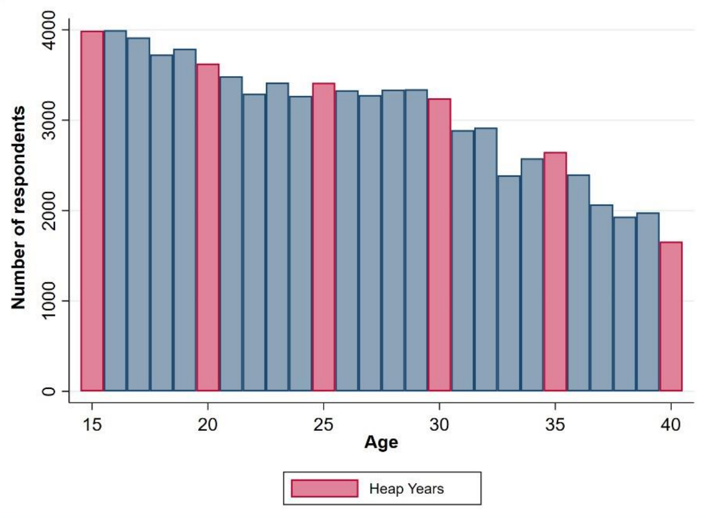

Notes: Figure shows the number of respondents by age (birth cohort), separately for each census wave. Respondents affected by the education policy reform were ages ≤ 20 (census 2001) or ≤ 30 (census 2011). Source: Botswana Census 2001 and 2011.
