# Supplementary material for: Causal effect of children’s secondary education on parental health outcomes: findings from a natural experiment in Botswana
Source: BMJ Open. 2021 Jan 12;11(1):e043247. doi: 10.1136/bmjopen-2020-043247 (PMC7805356; doi:10.1136/bmjopen-2020-043247)
Supplement: Supplementary data [file bmjopen-2020-043247supp002.pdf]

Table S2. Descriptive statistics: subset of children living with at least one parent (*N*=29,226)

| *Subsample: children co-residing with ≥ 1 parent* | Pre-reform cohorts (n = 9,467) | | Post-reform cohorts (n = 19,759) | |
|---|---|---|---|---|
| | Census 2001 | Census 2011 | Census 2001 | Census 2011 |
| *Children's characteristics* | | | | |
| Any parental disability, % | 5.7 | 6.4 | 4.8 | 3.5 |
| Age, mean (SD) | 23.1 (1.7) | 33.3 (1.7) | 18.9 (0.8) | 23.1 (3.7) |
| Years of schooling, mean (SD) | 9.1 (3.3) | 9.1 (3.6) | 9.5 (2.8) | 10.4 (3.1) |
| Has at least ten years of schooling, % | 45.0 | 42.1 | 68.2 | 78.0 |
| Labor force participation, % | 58.2 | 78.2 | 32.4 | 59.8 |
| Speaks Setswana at home, % | 84.5 | 86.6 | 83.0 | 83.0 |
| Christian, % | 71.8 | 77.4 | 73.4 | 79.3 |
| *Household characteristics* | | | | |
| Number of family members in household, mean (SD) | 8.0 (3.6) | 7.7 (3.8) | 7.6 (3.5) | 7.1 (3.5) |
| Any deaths in household last year, % | 7.6 | 5.1 | 6.9 | 4.0 |
| Ownership of dwelling, % | 90.0 | 94.3 | 87.0 | 86.3 |
| Access to electricity, % | 20.9 | 53.6 | 21.1 | 53.2 |
| Access to piped water, % | 14.6 | 21.1 | 16.1 | 25.1 |
| Telephone availability, % | 37.3 | 18.6 | 35.8 | 16.9 |
| Cellular phone availability, % | - | 95.0 | - | 95.1 |
| Receives remittances, % | 24.1 | 35.0 | 24.0 | 32.9 |

Sample includes survey respondents who were citizens of Botswana, born in Botswana, at least 18 years old at the time of the census, born in or after 1975, and who co-resided with at least one parent at the time of the census. The unit of analysis was children born either during the pre-reform period (prior to 1981) or post-reform period (in or after 1981). SD=Standard Deviation. Source: Botswana Census 2001 and 2011.
