# Supplementary material for: Causal effect of children’s secondary education on parental health outcomes: findings from a natural experiment in Botswana
Source: BMJ Open. 2021 Jan 12;11(1):e043247. doi: 10.1136/bmjopen-2020-043247 (PMC7805356; doi:10.1136/bmjopen-2020-043247)
Supplement: Supplementary data [file bmjopen-2020-043247supp003.pdf]

Table S3. OLS and ITT results controlling for parental socio-demographic characteristics

Dependent variable	**Parental disability (1=yes, 0=no)**		
Subsample	**Daughters**	**Sons**	**Both sexes**
	Risk difference (95% CI)		
A: OLS model			
Schooling (years)	-0.1*	-0.1	-0.1**
	(-0.2, 0.0)	(-0.2, 0.0)	(-0.2, -0.0)
B: OLS model			
≥ 10 years of schooling (1=yes,	-0.5	-1.0**	-0.7**
	(-1.3, 0.3)	(-1.9, -0.1)	(-1.3, -0.2)
C: ITT model			
Reform indicator	-0.3	-1.1	-0.7
	(-1.8, 1.1)	(-2.7, 0.5)	(-1.8, 0.4)
Additional controls			
Maternal age (years)	✓	✓	✓
Maternal schooling (years)	✓	✓	✓
Observations	15,006	12,127	27,133

Notes: Panels A and B show regression results from 'conventional' multivariable OLS models controlling for single-year age indicators, a continuous trend in year of birth, district of birth, as well as maternal age and maternal educational attainment (years of schooling completed). Regressions for the subsample with both children's sexes additionally control for an indicator for children's sex and interactions of each covariate with children's sex. Our models are robust to period effects, which we controlled for implicitly by simultaneously adjusting for children's age and year of birth. Panel C shows regression results from an ITT model (OLS), in which exposure to the reform was defined as a binary indicator (1=year of birth > 1980; 0=otherwise). The sample includes survey respondents who were citizens born in Botswana, at least 18 years old at the time of the census, born in or after 1975, and co-resided with at least their mother at the time of the census. Binary outcomes were multiplied by 100 to facilitate the interpretation of coefficients and standard errors on a % point scale. 95% robust confidence intervals in parentheses. *** p<0.01, ** p<0.05, * p<0.1. Source: Botswana Census 2001 and 2011.
