# Supplementary material for: Causal effect of children’s secondary education on parental health outcomes: findings from a natural experiment in Botswana
Source: BMJ Open. 2021 Jan 12;11(1):e043247. doi: 10.1136/bmjopen-2020-043247 (PMC7805356; doi:10.1136/bmjopen-2020-043247)
Supplement: Supplementary data [file bmjopen-2020-043247supp011.pdf]

Figure S4. Parental survival and disability by children's year of birth

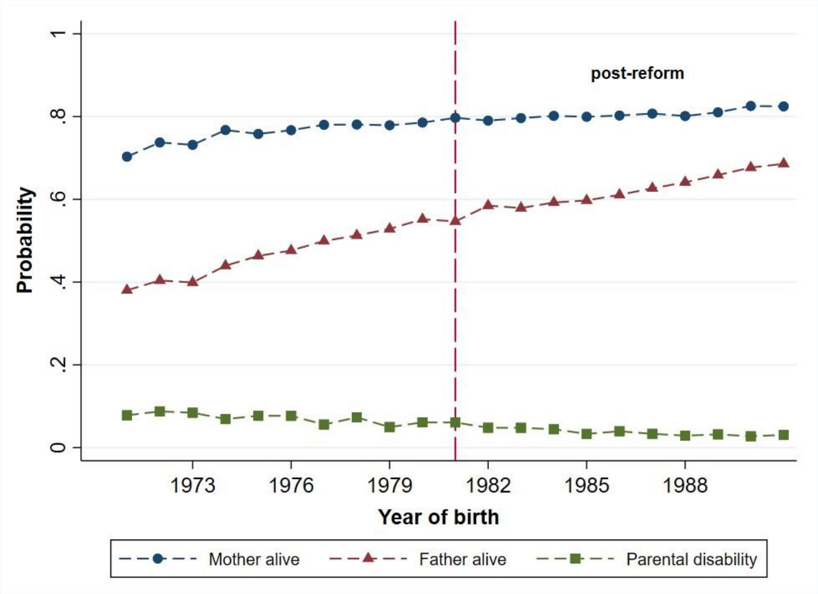

Notes: Figure shows survival of mothers (blue) and fathers (red) as well as parental disability (green) by children's birth cohort. The vertical dashed line indicates the timing of the policy reform. Parental disability was defined as any parental disability among the surviving mother and/or father. The sample for parental survival includes survey respondents who were citizens of Botswana and born in Botswana between 1971 and 1991; whereas the sample for disability additional includes only those children living with at least one parent. Source: Botswana Census 2011.
