# Supplementary material for: Causal effect of children’s secondary education on parental health outcomes: findings from a natural experiment in Botswana
Source: BMJ Open. 2021 Jan 12;11(1):e043247. doi: 10.1136/bmjopen-2020-043247 (PMC7805356; doi:10.1136/bmjopen-2020-043247)
Supplement: Supplementary data [file bmjopen-2020-043247supp004.pdf]

Table S4. ITT results: testing the robustness of our findings to alternative specifications in children's age, YOB, additional controls for heap year, slope change in YOB, birth cohort windows, and analytical sample (outcome: maternal survival)

Dependent variable: mother alive (1=yes, 0=no)<br>***Sample: both sexes***<br>***Model: ITT***	**(1)**	**(2)**	**(3)**	**(4)**	**(5)**	**(6)**	**(7)**	**(8)**	**(9)**	**(10)**	**(11)**	**(12)**
Coefficient on endogenous variable (child)												
Reform indicator	-0.2	-0.2	0.1	0.4	0.8	0.6	-0.1	-0.2	-0.6	-0.6	0.7	-0.0
	(0.6)	(1.0)	(0.5)	(0.4)	(0.7)	(0.6)	(0.4)	(0.6)	(1.0)	(0.6)	(0.9)	(1.2)
Basic covariates (child)												
i.Age##i.Sex	✓	✓	-	-	-	-	-	✓	✓	✓	✓	✓
c.YOB##i.Sex	✓	✓	✓	✓	✓	✓	✓	✓	✓	✓	✓	✓
i.CensusYear##i.Sex	✓	✓	✓	✓	✓	✓	✓	✓	✓	✓	✓	✓
i.BirthDistrictr##i.Sex	✓	✓	✓	✓	✓	✓	✓	✓	✓	✓	✓	✓
Additional controls (child)												
c.YOB2##i.Sex	-	✓	-	-	-	-	-	-	-	-	-	-
i.ThreeYearAgeGroup##i.Sex	-	-	✓	-	-	-	-	-	-	-	-	-
c.Age##i.Sex	-	-	-	✓	✓	✓	✓	-	-	-	-	-
c.Age2##i.Sex	-	-	-	✓	✓	✓	✓	-	-	-	-	-
c.Age3##i.Sex	-	-	-	✓	-	-	-	-	-	-	-	-
c.Age4##i.Sex	-	-	-	✓	-	-	-	-	-	-	-	-
i.HeapYear##i.Sex	-	-	-	-	-	-	-	✓	-	-	-	-
c.YOB#i.Reform_Indicator, c.YOB#i.Reform_Indicator#i.Sex	-	-	-	-	-	-	-	-	✓	-	-	-
Birth cohort windows (child)												
Narrower birth cohort window, 1978 ≤ YOB ≤ 1984	-	-	-	-	✓	-	-	-	-	-	-	-
Narrower birth cohort window, 1976 ≤ YOB ≤ 1986	-	-	-	-	-	✓	-	-	-	-	-	-
Earlier birth cohorts included, 1971 ≤ YOB ≤ 1993	-	-	-	-	-	-	✓	-	-	-	-	-
Subsample, children with ≥ 9 years of schooling	-	-	-	-	-	-	-	-	-	✓	-	-
Subsample, co-resides with parent and gap ≥ 5.5 years of schooling	-	-	-	-	-	-	-	-	-	-	✓	-
Subsample, co-resides with parent and paternal age ≥ 50 years	-	-	-	-	-	-	-	-	-	-	-	✓
Observations	89,721	89,721	89,721	89,721	41,942	59,866	106,774	89,721	89,721	71,608	14,547	9,691

Notes: Robustness checks for the intention-to-treat (ITT) regression results shown in column 4 in Table 3 in the main text (maternal survival). The sample in models 1–4 and models 8–12 includes survey respondents who were citizens born in Botswana, at least 18 years old at the time of the census, and born in or after 1975. Binary outcomes were multiplied by 100 to facilitate the interpretation of coefficients and standard errors on a % point scale. Robust standard errors in parentheses. *** p<0.01, ** p<0.05, * p<0.1. Source: Botswana Census 2001 and 2011. YOB=year of birth.
