# Supplementary material for: Causal effect of children’s secondary education on parental health outcomes: findings from a natural experiment in Botswana
Source: BMJ Open. 2021 Jan 12;11(1):e043247. doi: 10.1136/bmjopen-2020-043247 (PMC7805356; doi:10.1136/bmjopen-2020-043247)
Supplement: Supplementary data [file bmjopen-2020-043247supp006.pdf]

Table S6. OLS and ITT results: stratified by parental age group

Dependent variable	**Parental disability (1=yes, 0=no)**		
Parental age group	**Age 50-59**	**Age 60-69**	**Age 70-79**
	Risk difference (95% CI)		
A: OLS model			
Schooling (years)	-0.0	-0.3***	-0.1
	(-0.2, 0.1)	(-0.5, -0.1)	(-0.6, 0.3)
B: OLS model			
$\geq$ 10 years of schooling (1=yes, 0=no)	-0.5	-1.6**	-2.0
	(-1.3, 0.3)	(-3.0, -0.2)	(-4.9, 0.9)
C: ITT model			
Reform indicator	-0.6	-1.0	-2.1
	(-2.4, 1.3)	(-4.6, 2.5)	(-10.2, 6.1)
Probability dependent variable, pre-reform	5.3	8.2	13.9
Observations	12,447	6,329	2,262

Notes: Panels A and B show regression results from an OLS model controlling for single-year age indicators, a continuous trend in year of birth, district of birth, and the interaction of each covariate with children's sex. Our models are robust to period effects, which we controlled for implicitly by simultaneously adjusting for children's age and year of birth. Panel C shows regression results from an ITT model (OLS), in which exposure to the reform was defined as a binary indicator (1=year of birth > 1980; 0=otherwise). The sample includes citizens born in Botswana, at least 18 years old at the time of the census, and born in or after 1975. Binary outcomes were multiplied by 100 to facilitate the interpretation of coefficients and standard errors on a % point scale. 95% robust confidence intervals in parentheses. *** p<0.01, ** p<0.05, * p<0.1. Source: Botswana Census 2001 and 2011.
