# Supplementary material for: Causal effect of children’s secondary education on parental health outcomes: findings from a natural experiment in Botswana
Source: BMJ Open. 2021 Jan 12;11(1):e043247. doi: 10.1136/bmjopen-2020-043247 (PMC7805356; doi:10.1136/bmjopen-2020-043247)
Supplement: Supplementary data [file bmjopen-2020-043247supp007.pdf]

Table S7. OLS and ITT results: logistic regression models.

Dependent variable	**Mother alive (1=yes, 0=no)**			**Father alive (1=yes, 0=no)**		
Subsample	**Daughters**	**Sons**	**Both sexes**	**Daughters**	**Sons**	**Both sexes**
	Odds ratios (95% CI)					
A: OLS model						
Schooling (years)	1.063***	1.048***	1.055***	1.035***	1.023***	1.028***
	(1.056, 1.071)	(1.041, 1.056)	(1.050, 1.060)	(1.029, 1.041)	(1.017, 1.029)	(1.024, 1.033)
B: OLS model						
≥ 10 years of schooling (1=yes, 0=no)	1.402***	1.309***	1.356***	1.199***	1.091***	1.145***
	(1.328, 1.479)	(1.238, 1.384)	(1.304, 1.409)	(1.148, 1.252)	(1.044, 1.141)	(1.110, 1.181)
C: ITT model						
Reform indicator	0.988	1.054	1.018	1.094	1.052	1.074
	(0.776, 1.258)	(0.816, 1.362)	(0.854, 1.214)	(0.913, 1.311)	(0.869, 1.274)	(0.942, 1.225)
Observations	47,121	42,600	89,721	47,121	42,600	89,721

Notes: Logistic regression models controlling for single-year children's age indicators, a continuous trend in children's year of birth and year of birth squared, and children's district of birth. Regressions for the subsample with both sexes additionally control for an indicator for children's sex and interactions of each covariate with children's sex. Our models are robust to period effects, which we controlled for implicitly by simultaneously adjusting for children's age and year of birth. Panel C shows regression results from an intention-to-treat (ITT) model in which exposure to the reform was defined as a binary indicator (1=year of birth > 1980; 0=otherwise). The sample includes survey respondents who were citizens born in Botswana, at least 18 years old at the time of the census, and born in or after 1975. Robust 95% confidence intervals in parentheses. *** p<0.01, ** p<0.05, * p<0.1. Source: Botswana Census 2001 and 2011.
