# Supplementary material for: Causal effect of children’s secondary education on parental health outcomes: findings from a natural experiment in Botswana
Source: BMJ Open. 2021 Jan 12;11(1):e043247. doi: 10.1136/bmjopen-2020-043247 (PMC7805356; doi:10.1136/bmjopen-2020-043247)
Supplement: Supplementary data [file bmjopen-2020-043247supp012.pdf]

Figure S5. Bias component plots

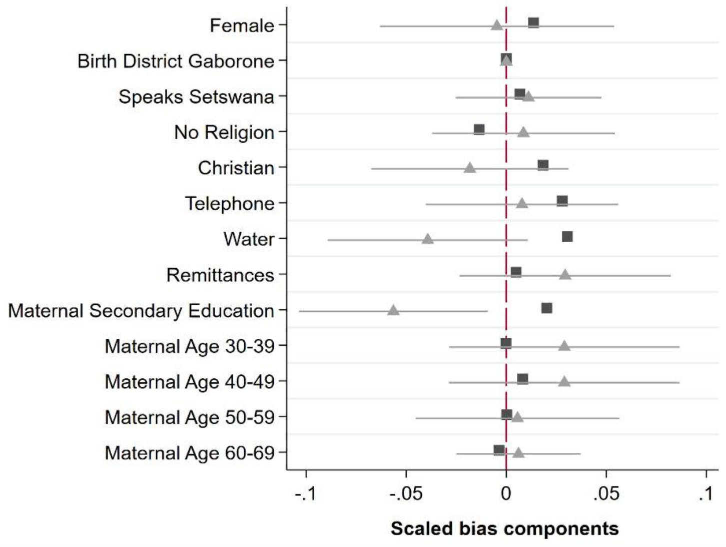

Notes: Bias component plots for the difference in children's sex, birth district, language, religion, household assets, household remittances, parental education, and parental age, by actual exposure (squares) and proposed instrument (triangles). The figures for the instrumental variable results account for the strength of the instrument as described in Jackson and Swanson (2015) and Davies (2017). All variables are binary. The horizontal lines indicate robust confidence intervals for years of schooling. Sample includes survey respondents who were citizens born in Botswana, at least 18 years old at the time of the census, born in or after 1975, and co-resided with at least one parent at the time of the census. Source: Botswana Census 2001 and 2011.
