# Supplementary material for: Causal effect of children’s secondary education on parental health outcomes: findings from a natural experiment in Botswana
Source: BMJ Open. 2021 Jan 12;11(1):e043247. doi: 10.1136/bmjopen-2020-043247 (PMC7805356; doi:10.1136/bmjopen-2020-043247)
Supplement: Author's manuscript [file bmjopen-2020-043247.draft_revisions.pdf]

BMJ Open is committed to open peer review. As part of this commitment we make the peer review history of every article we publish publicly available.

When an article is published we post the peer reviewers' comments and the authors' responses online. We also post the versions of the paper that were used during peer review. These are the versions that the peer review comments apply to.

The versions of the paper that follow are the versions that were submitted during the peer review process. They are not the versions of record or the final published versions. They should not be cited or distributed as the published version of this manuscript.

BMJ Open is an open access journal and the full, final, typeset and author-corrected version of record of the manuscript is available on our site with no access controls, subscription charges or pay-per-view fees (http://bmjopen.bmj.com).

If you have any questions on BMJ Open's open peer review process please email info.bmjopen@bmj.com

Causal Effect of Children's Education on Parental Health – Findings from a Natural Experiment in Botswana

Journal:	*BMJ Open*
Manuscript ID	bmjopen-2020-043247
Article Type:	Original research
Date Submitted by the Author:	29-Jul-2020

Complete List of Authors:	Ludwig, Jan Ole; Heidelberg Univ, Institute of Global Health, Medical Faculty and University Hospital<br>Davies, Neil; University of Bristol, Medical Research Council Integrative Epidemiology Unit; Bristol Medical School, Population Health Sciences<br>Bor, Jacob; Boston University, Global Health; Boston University School of Public Health, Department of Epidemiology<br>De Neve, JW; Heidelberg Univ, Institute of Global Health, Medical Faculty and University Hospital

Keywords:	EPIDEMIOLOGY, HEALTH ECONOMICS, PUBLIC HEALTH

SCHOLARONE™
Manuscripts

I, the Submitting Author has the right to grant and does grant on behalf of all authors of the Work (as defined in the below author licence), an exclusive licence and/or a non-exclusive licence for contributions from authors who are: i) UK Crown employees; ii) where BMJ has agreed a CC-BY licence shall apply, and/or iii) in accordance with the terms applicable for US Federal Government officers or employees acting as part of their official duties; on a worldwide, perpetual, irrevocable, royalty-free basis to BMJ Publishing Group Ltd ("BMJ") its licensees and where the relevant Journal is co-owned by BMJ to the co-owners of the Journal, to publish the Work in this journal and any other BMJ products and to exploit all rights, as set out in our licence.

The Submitting Author accepts and understands that any supply made under these terms is made by BMJ to the Submitting Author unless you are acting as an employee on behalf of your employer or a postgraduate student of an affiliated institution which is paying any applicable article publishing charge ("APC") for Open Access articles. Where the Submitting Author wishes to make the Work available on an Open Access basis (and intends to pay the relevant APC), the terms of reuse of such Open Access shall be governed by a Creative Commons licence – details of these licences and which Creative Commons licence will apply to this Work are set out in our licence referred to above.

Other than as permitted in any relevant BMJ Author's Self Archiving Policies, I confirm this Work has not been accepted for publication elsewhere, is not being considered for publication elsewhere and does not duplicate material already published. I confirm all authors consent to publication of this Work and authorise the granting of this licence.

For peer review only

Causal Effect of Children's Education on Parental Health – Findings from a Natural Experiment in Botswana

Authors: Jan Ole Ludwig, B.M.[1], Neil M. Davies, Ph.D.[2,3,4], Jacob Bor Sc.D. S.M.[5,6], Jan-Walter De Neve, M.D. Sc.D.[1,*]

Affiliations:

[1] Heidelberg Institute of Global Health, Medical Faculty and University Hospital, Heidelberg University, Im Neuenheimer Feld 130.3, Heidelberg 69120, Germany.

[2] Medical Research Council Integrative Epidemiology Unit, University of Bristol, BS8 2BN, United Kingdom.

[3] Population Health Sciences, Bristol Medical School, University of Bristol, Barley House, Oakfield Grove, Bristol, BS8 2BN, United Kingdom.

[4] K.G. Jebsen Center for Genetic Epidemiology, Department of Public Health and Nursing, NTNU, Norwegian University of Science and Technology, Norway.

[5] Department of Global Health, Boston University School of Public Health, Boston, MA 02118, United States.

[6] Department of Epidemiology, Boston University School of Public Health, Boston, MA 02118, United States.

For peer review only

Acknowledgments:

We thank respondents in the Botswana 2001 and 2011 Census, and staff at the Botswana Central Statistics Office. We also thank Prashant Bharadwaj for helpful comments. JOL was supported by the Else Kröner-Fresenius-Stiftung within the Heidelberg Graduate School of Global Health (grant number: not applicable). NMD was supported by an Economics and Social Research Council (ESRC) Future Research Leaders grant (grant number: ES/N000757/1) and a Norwegian Research Council grant (grant number: 295989). JB and JWDN were supported by the NICHD of National Institutes of Health (grant number: R03-HD098982). JWDN was also supported by the Alexander von Humboldt Foundation, funded by Germany's Federal Ministry of Education and Research Health (grant number: not applicable), German Research Foundation (grant number: 405898232), and Heidelberg University's Excellence Initiative Health (grant number: not applicable). The funders had no role in study design, data collection and analysis, decision to publish, or preparation

of the manuscript. The contents are the responsibility of the authors and do not necessarily reflect the views of any of the funders or the US government.

Contributions Statement:

JOL and JWDN conceived and designed the study. JL conducted the statistical analyses under the guidance of NMD, JB, and JWDN. JOL wrote the first draft of the report. NMD, JB, and JWDN reviewed and contributed important revisions to the report. All authors approved the final submitted version of the report.

Data Sharing Statement:

Data is publicly available at no cost from IPUMS (http://ipums.org).

Conflict of Interest:

The authors declare that they have no conflict of interest.

***Correspondence:**

Jan-Walter De Neve, Heidelberg Institute of Global Health, Medical Faculty and University Hospital, Heidelberg University, Im Neuenheimer Feld 130.3, Heidelberg 69120, Germany. E-mail: janwalter.deneve@uni-heidelberg.de. Phone: +49 (0) 6221 56-32873.

For peer review only

Abstract

Objectives: A growing literature highlights the intergenerational transmission of human capital, from parents to children. However, far less is known about "upward transmission", from children to parents. In this study we use a 1996 Botswana education policy reform as a natural experiment to identify the causal effect of children's secondary schooling on their parents' health.

Setting: Botswana's decennial census (2001 and 2011). Data were obtained through the Integrated Public Use Microdata Series (IPUMS) and are 10% random samples of the complete population in each of these census years (i.e., 'full censuses').

Participants: Survey respondents who were citizens born in Botswana, at least 18 years old at the time of the census, and born in or after 1975 (N = 89,721).

Primary and secondary outcome measures: Parental survival and disability at the time of the census, separately for mothers and fathers.

Results: The 1996 reform caused a large increase in grade ten enrollment, inducing an additional 0.4 years of schooling for the first cohorts affected (95% Confidence Interval: 0.3 – 0.5, $P < 0.001$). The reform, however, had no effect on parental survival and disability by the time exposed child cohorts reach age 30. Results were robust to a wide array of sensitivity analyses.

Conclusions: This study found little evidence that parents' survival and disability was affected by their offspring's educational attainment in Botswana. Parents' health may not be necessarily affected by increasing their offspring's educational attainment.

Keywords: Children's Education; Parental Health; Intergenerational; Natural Experiment; Instrumental Variable Analysis; Low- and Middle-Income Countries

For peer review only

Strengths and Limitations of this Study

- While a large literature has investigated the role of parental human capital on children's well-being, relatively little is known about "upward transmission", from children to parents.
- We explore the 1996 Botswana education policy reform as a natural experiment to estimate the causal effect of children's secondary education on their parents' health.
- Using nearly 90,000 census records from 2001 and 2011, we show that the 1996 reform resulted in an additional 0.4 years of schooling among exposed child cohorts.
- Children's education, however, had few detectable effects on parental survival and disability. Parents' health may not be affected by increasing their offspring's education.
- Limitations of our study include limited generalizability beyond the reform analyzed and lack of information on alternative parental health outcomes.

For peer review only

1. Introduction

Investments in children's human capital may yield large dividends not only for themselves (1) and their offspring (2, 3), but also for their parents. While a large literature has documented the substantial resource flows from parents to children, a growing literature from Asia (4), Latin America (5), and sub-Saharan Africa (6) suggests that intergenerational wealth transfers have started to reverse in aging low- and middle-income societies where governmental support for older adults is limited (7). In addition to sharing their wealth with parents, better-educated children may share their increased knowledge (8) and skills (9) with older household members, and may be better equipped to interact with the health system to help manage the health needs of their aging parents. Additional education may also open access to careers in the health sector, leading to increased intra-family medical expertise, with positive health benefits for family members (10). Adult children have been suggested to "repay" their parents for investments received in childhood, particularly in settings where families function as de facto social institutions (11).

The past decades have brought a massive increase in educational opportunities for younger cohorts in low- and middle-income countries (LMICs). Gross domestic product per capita in LMICs more than doubled between 2000 and 2018, from 2,310 to 4,662 in 2010 USD (12). The extent to which this younger generation shares its increased resources with their parents, however, remains largely unknown. More than a dozen studies have reported a positive correlation between children's human capital and parental health, including in China (13) and South Africa (14). Given the large number of potential factors (such as genetic traits and reverse causality) that may confound the relationship between children's human capital and parental health, however, these correlations are difficult to interpret and are unlikely to identify causal effects of educational opportunities

For peer review only - http://bmjopen.bmj.com/site/about/guidelines.xhtml

provided to children. A recent review concluded that spillover effects from offspring to parents are understudied and (quasi-)experimental studies from low-resource settings are needed (15).

In this study, we explore a large secondary schooling reform in sub-Saharan Africa to determine the impact of increasing educational investment in younger generations on their parents. In 1996, the government of Botswana launched a major education reform. Between 1997 and 1998, grade ten enrollment in the country more than doubled, from 15,437 to 36,562 (**Figure** 1, see **Text S1** in the supplementary materials for additional background). At the cohort level, the reform induced an additional 0.4 years of schooling among affected cohorts relative to the attainment the affected cohorts would have achieved if previous trends had continued. This reform constitutes a 'natural experiment' through comparison of cohorts exposed to the reform versus those unexposed, while controlling for gradual changes across cohorts. Using two waves of Botswana's decennial census (2001, 2011) to disentangle age and cohort effects, we used the resulting variation in exposure to identify the causal effect of children's secondary education on parental health.

(FIGURE 1 AROUND HERE)

only

For peer review only - http://bmjopen.bmj.com/site/about/guidelines.xhtml

2. Material and Methods

2.1. Data Sources and Study Population

Data for this study were obtained from the Botswana Population and Housing Census 2001 and 2011 through the Integrated Public Use Microdata Series (IPUMS) (16). The microdata provided by IPUMS are 10% random samples of the complete population in each of these years (i.e., 'full censuses'). Subnational administrative boundaries were harmonized across censuses by IPUMS. The censuses were conducted by the Central Statistics Office Botswana and included all persons in the country at the time of the census without regard of their usual residence. The censuses also collected basic demographic information on members of the household who were citizens outside of the country on the census night, but who would typically live in the household if they would have been in Botswana. In the 2011 Census, for instance, 24,561 citizens were living outside of Botswana (1.2%). Data on sex, age, years of schooling, and the survival status of mothers and fathers was available for 88.9% of respondents, yielding a total of 333,334 individuals in the pooled 2001 and 2011 census dataset (**Figure 2**).

(FIGURE 2 ABOUT HERE)

The study population included all citizens of Botswana and born in Botswana. Children ages younger than 18 were excluded because they would not have had the opportunity to complete secondary school. Children born prior to 1975 were excluded because previous secondary school reforms led to changes in education for these older cohorts. Using a narrow subsample of pre- and

For peer review only - http://bmjopen.bmj.com/site/about/guidelines.xhtml

post-reform cohorts (i.e., born between 1975 and 1993 based on the age and birth cohort cutoffs described above), there were good reasons to believe that control cohorts differed from exposed cohorts mostly with respect to their access to grade ten. We explore alternative sample specifications in sensitivity analyses (**Section 2.6**). Children not born in Botswana were excluded because they would not have been exposed to the reform if they migrated in adulthood.

2.2. Measurement of Exposure and Endpoints

Data on education, age in years, gender, citizenship, district of birth, parental survival, parental disability, parental age, and parental years of schooling were extracted from the census datasets. Year of birth was calculated based on age collected in the census. The key independent variable of interest was the child's total years of schooling ("What is the highest grade/level that [respondent] has completed?"). Only formal schooling was counted. Years of school accounts for the number of years of study regardless of the track or kind of study. Our main outcome of interest was parental survival status, separately for mothers and fathers. Parental survival status indicated whether the person's biological parent was alive at the time of the census (e.g., "Is [the person's] biological mother alive?") and was observed regardless of whether the parent is co-resident with the adult child. During the period of study, Botswana had very high adult mortality rates, due in large part to the country's HIV epidemic. In 2001, the probability of a 15-year old reaching age 60 ($_{45}e_{15}$) was about 37% (17). In this context, children's education could affect parental survival because information about HIV was disseminated via secondary school "life skills" curricula (18); because HIV stigma may be lower among the better educated, leading to higher HIV testing (19); and/or because better educated children may help their parents navigate the health system and gain access to HIV treatment (9). Other major determinants of morbidity and mortality among adults ages 50 – 69 years in Botswana include cardiovascular diseases, neoplasms, and diabetes and

For peer review only - http://bmjopen.bmj.com/site/about/guidelines.xhtml

kidney diseases (20). Diabetic retinopathy and peripheral neuropathy, for instance, are increasingly common causes of severe visual impairment and non-traumatic lower extremity disability, including in the context of sub-Saharan Africa.

Pointer variables provided by IPUMS indicate the location within the household of every child's parent, and make it possible to construct individual-level variables representing the characteristics of co-resident parents. For the subset of households with surviving, co-resident parents and children, we were therefore able to assess the impact of the schooling reform on key parental socio-economic characteristics and disability, as additional secondary outcomes. Children's education, for instance, has been suggested to affect parental fertility decisions by creating additional costs (educational expenses) and decreasing the perceived benefits derived from children by removing them from the family productive system (21). The census also asked a series of questions about disability in the 2001 and 2011 waves. Specifically, they asked about employment disability, blind- or vision-impaired, deaf or hearing-impaired, mute or speech-impaired, disability affecting lower extremities, disability affecting upper extremities, mental disability, and psychological disability. For our analysis, we coded the following parental disability variables: "employment disability" defined as whether a parent was economically inactive because of health-related reasons; "blind or vision-impaired" defined as whether a parent was blind or had limited vision; and "disability affecting lower extremities" defined as whether a parent was unable to use one or both legs (16). Disabilities were reported by a household respondent (typically the household head). Data on financial transfers between children and their parents, frequency of contact between children and parents, and the uptake of social or healthcare services were not collected during the census.

For peer review only - http://bmjopen.bmj.com/site/about/guidelines.xhtml

2.3. Unit of Analysis

Our unit of analysis was the adult child. In doing so, we follow the data structure and variables provided by IPUMS closely, which maps well unto our analytical approach since our identification strategy is based on cohort-specific variation to the education reform at the child level. Moreover, the census datasets do not contain information on all offspring's educational attainment which precludes an analysis at the parent level (16). The census datasets thus differ from more standard surveys focused on older adults and which contain information on the educational attainment of all offspring (e.g., the Health and Retirement Study in the United States). For our main analysis, we included adult children regardless of whether they were the head of household, their marital status, or whether the adult child co-resided with their parent(s).

2.4. Instrumental Variable Analysis

An instrumental variable (IV) is a variable that is associated with the treatment of interest, but is independent of confounding factors and has no direct effect on the outcome (22). In our application, the instrument was a 'reform cohort' indicator, taking the value one if the respondent was born in a cohort that was exposed to the education policy reform and zero otherwise. In Botswana, children were historically expected to start primary school at age seven years and enter junior secondary school at age 15 years. Individuals born in 1981 or later would therefore have entered junior secondary school in 1996 or later and were classified as exposed (**Figure S1**). We used this exogenous variation in educational attainment resulting from the policy reform to identify the causal effect of children's education on their parents' survival. First, we assessed whether children's cohorts exposed to the reform had higher educational attainment than birth cohorts not exposed to the reform using multivariable adjusted ordinary least squares (OLS) linear

For peer review only - http://bmjopen.bmj.com/site/about/guidelines.xhtml

probability regression models. We estimated the effect of exposure to the reform on total years of schooling completed and the probability of completing at least ten years of schooling (**Table 2**). Second, as a benchmark for our causal analysis, we estimated the 'naïve' cross-sectional association of children's education with their parents' survival, acknowledging that this association may reflect reverse causality and effects of confounders and therefore does not have a causal interpretation. We assessed the crude relationship graphically and in multivariable adjusted regression analyses (*Panels A* and *B*, **Table 3**). Third, we assessed the intention-to-treat (ITT) effect of having a child affected by the reform on parental survival. We examined the relationship graphically and adjusted for covariates (*Panel C*, **Table 3**). Fourth, we estimated 2SLS (instrumental variables) regression models, using exposure to the reform as an instrument for total years of children's schooling while adjusting for covariates (*Panel D*, **Table 3**). Natural experiments that change the probability of an exposure can be analyzed like randomized controlled trials with non-compliance. Exposure to the reform was modeled as an intercept shift for cohorts affected by the reform. Specifically, we estimated models of the form:

$$(1) \qquad Prob(YearsofSchooling_i|X_i, ReformIndicator_i) = \alpha\ ReformIndicator_i + \boldsymbol{\beta}\ \boldsymbol{X_i}$$

$$(2) \qquad Prob(ParentalSurvival_i=1|X_i, YearsofSchooling\_hat_i) = \gamma\ YearsofSchooling\_hat_i + \boldsymbol{\delta}\ \boldsymbol{X_i}$$

where $\boldsymbol{X_i} = [Children'sAge_i, f(Children'sYearofBirth), Children'sDistrictofBirth_i]$

In this model, γ estimates the causal effect of schooling on parental survival if the error terms in the linear models (1) and (2) are independent. In all models, we controlled for children's single-year age indicators ($Children'sAge_i$) to account for trends in age and education across children's cohorts. We focused on this age specification as it is highly flexible, but assess alternate specifications in sensitivity analyses. We also included a linear term in children's year of birth and

For peer review only - http://bmjopen.bmj.com/site/about/guidelines.xhtml

indicators for children's district of birth. We estimated models for women and men separately, as well as a pooled sample. When pooling sexes, we additionally included children's sex and the interactions of sex with all other covariates. Data on parent's own socio-demographic characteristics was also available for the sub-sample of children co-residing with a least one parent. When testing the association between children's education and secondary parental outcomes in a 'conventional' multivariable regression analysis, we therefore additionally included controls for parental age (years) and parental educational attainment (years of schooling).

2.5. Assumptions for Causal Inference

For a causal interpretation of our effect estimates, four assumptions must be satisfied (see **Figure S2** for a directed acyclic graph). First, our instrument must be associated with educational attainment. This is testable and we found evidence of large effects (F-statistic: 65.6, **Table 3**). The large value of the F-statistic indicates that our instrument was strongly associated with schooling. Second, the instrument must be independent of unobserved confounders of the instrument-outcome association conditional on observed covariates. This implies that children born before and after the reform cutoff were similar, after controlling for children's single-year age indicators, a continuous term in children's year of birth, and children's district of birth. Critically, the availability of multiple census years allowed us to generate variation in children's age in a given birth cohort group. Our models are therefore also robust to the existence of period effects, which we control for by simultaneously adjusting for children's age and a continuous term in year of birth (noting that census_year = birth cohort + age). We also assessed alternative specifications of children's age (quadratic, cubic, quartic) and year of birth (quadratic), and restricted the sample to a narrower birth cohort window around the reform cutoff (+/- 3 years and +/- 5 years around

the cutoff). We also allowed for different linear slopes in year of birth on either side of the reform cutoff akin to a 'fuzzy' regression discontinuity design (23).

We also used covariate balance plots to estimate the association of our exposure (schooling) and our instrument (reform indicator) with observed covariates, as described in detail elsewhere (24, 25). While this analysis cannot prove that our instrument is not associated with unobserved covariates, it can provide some indication if our instrument is less strongly associated with measured covariates than our exposure. Third, for our 2SLS estimates to have a causal interpretation, we assume that exposure to the policy reform affected our parental outcomes only through changes in secondary schooling. This assumption is plausible given that the education reform was a supply-side intervention (18), which would not have affected parents except through their children's increased access to school. Fourth, to interpret our results as local average treatment effects, we assume that there are no defiers (i.e., the monotonicity assumption) (26).

2.6. Sensitivity Analyses

In addition to the robustness checks described above, we conducted a wide range of sensitivity analyzes, including earlier birth cohorts (i.e., those born between 1971 and 1993, instead of those born between 1975 and 1993), modelling the outcome using a logit link function, and controlling for an indicator for heap year (defined as ages that are a multiple of five) to account for measurement error in age due to potential age heaping. Results could also be biased by differential response rates by birth cohort, manipulation of our 'running variable', or by selection bias from mortality risk or migration associated with being born after 1980. We therefore assessed cohort sizes by birth cohort (**Figure S3**). We note that we present our results for secondary parental outcomes under the null hypothesis that there is no effect of the reform on parental survival.

For peer review only - http://bmjopen.bmj.com/site/about/guidelines.xhtml

To further probe the robustness of our main findings, we conducted a number of subsample analyses. Specifically, we included households for whom additional education among children was most likely to have relatively large consequences for parental survival. Our hypothesis was that the role of children's resources may be more pronounced in households where: i) children were more likely to be affected by the reform, ii) there was a large intergenerational gap in educational attainment between adult children and their parents, and iii) parents were on average older and thus more likely to be 'dependent' on the support of children. To do so, we limited the sample to children with at least nine years of schooling (i.e., those primarily affected by the policy reform). For the subset of households with co-resident parents and children, we also limited the sample to households where the difference in educational attainment between parents and children was $\geq$ 5.5 years (50th percentile), and stratified our model by parental ten-year age groups.

2.7. Data, Code Availability, and Ethics

This was a complete case analysis and all analyses were conducted in StataMP 15.1. Data is publicly available at no cost from IPUMS (http://ipums.org) (16). Code used to generate these results can be found at https://github.com/jwdeneve/Botswana-ParentalHealth. This study was pre-registered and approved by the Heidelberg University Hospital Ethics Committee [S-186/2018].

2.8. Patient and Public Involvement

Patients or the public were not involved in the design, or conduct, or reporting, or dissemination plans of our research.

For peer review only - http://bmjopen.bmj.com/site/about/guidelines.xhtml

3. Results

3.1. Descriptive Statistics

On average the children in our analytical sample were 24.8 years old (**Table 1**). Across 34,202 children born pre-reform, 13,073 (38.2%) had lost their father and 5,666 (16.6%) had lost their mother. In terms of household characteristics, the average number of family members per household was 5.0. In our subset of households with surviving, co-resident parents and children, there were a total of 29,266 (32.6%) child-parent dyads (10,082 of which were living with both parents). Among those, the average parental age was 50.8 and 57.0 years for mothers and fathers, respectively. The average gap in attained education between children and co-residing parents was substantial (5.2 and 6.0 years of schooling for mothers and fathers, respectively). **Table S2** shows detailed descriptive statistics for the subset of children co-residing with at least one parent.

(TABLE 1 AROUND HERE)

3.2. Association Between Children's Education and Parental Survival

Figure 3 illustrates heterogeneity in the unadjusted relationship between children's education and parental survival, separately for maternal and paternal survival. The positive association between children's education and parental survival was non-linear, levelling off after junior secondary school. In 'conventional' multivariable adjusted regression analysis, children's schooling was associated with increased parental survival. In the pooled (daughters and sons) sample, each additional year of children's schooling was associated with a 0.8 percentage point increase (95%

For peer review only - http://bmjopen.bmj.com/site/about/guidelines.xhtml

Confidence Interval [CI]: 0.7 – 0.9) and a 0.6 percentage point increase (95% CI: 0.5 – 0.7) in the probability of maternal and paternal survival, respectively (**Table 3**, *Panel A*). These associations persisted after controlling for parent's own socio-demographic characteristics in the subset of households with co-resident parents and children (**Table S3**). Although suggestive, these associations may be vulnerable to reverse causality or confounding by unobserved characteristics.

(FIGURE 3 AROUND HERE)

3.3. Causal Effect of Children's Education on Parental Survival

Table 2 shows the estimated reform impact on children's educational attainment, as indicated by total years of schooling completed and the probability of having completed at least ten years of schooling. All models controlled flexibly for single-year age indicators, a continuous term in year of birth, indicators for district of birth, and census year. In the pooled sample, the estimated effect of the reform on educational attainment was 0.4 additional years of schooling (95% CI: 0.3 – 0.5) among exposed child cohorts. Exposure to the reform was also associated with a 19.0 percentage point increase (95% CI: 17.5 – 20.4) in the probability of having completed at least ten years of schooling relative to a baseline probability of 43.5% among pre-reform child cohorts.

(TABLE 2 AROUND HERE)

For peer review only - http://bmjopen.bmj.com/site/about/guidelines.xhtml

Figure S4 in the supplementary webappendix plots parental survival and disability against reform exposure. We found little evidence of a discontinuity for post-reform cohorts. Multivariable regression models – which adjusted for children's single-year age indicators, a continuous trend in children's year of birth, district of birth indicators, and census year indicators –, presented in **Table 3**, also provide little evidence (at alpha=0.05, with 80% power) that exposure to the reform affected maternal survival (-0.2 percentage points; 95% CI: -1.3 – 0.9) or paternal survival (0.5 percentage points; 95% CI: -0.9 – 2.0). Our results were generally not sensitive to different modelling strategies for age, different specifications of the outcome, sample inclusion criteria (**Tables S3** – **S6**) or model (**Table S7**). The differences in potential confounders for the actual exposure (schooling) and the proposed instrument (reform cohort) are presented in **Figure S5**. These results suggest that the instrumental variable analysis would be generally less biased from these measured potential confounders than the multivariable adjusted regression analysis. One variable appeared to differ for the instrument (maternal schooling); however, adjusting for this variable did not meaningfully affect our results.

(TABLE 3 AROUND HERE)

For peer review only - http://bmjopen.bmj.com/site/about/guidelines.xhtml

3.4. Children's Education and Secondary Parental Outcomes

In **Table 4**, we show results for our secondary parental outcomes, including key parental demographic and socio-economic characteristics and disability categories. Consistent with our main results for maternal and paternal survival, we found little evidence that children's exposure to the reform affected any of these secondary parental outcomes (*Panel C*, **Table 4**).

(TABLE 4 AROUND HERE)

4. Discussion

Using census data from 89,721 adult children, we find that additional secondary education in children had few detectable survival benefits for their parents in Botswana. Although children's education was strongly associated with improved parental health in OLS regression models, these results could be due to e.g., reverse causality whereby chronically ill parents require care from children before completion of school. Our ITT estimates, in contrast, suggest that it is unlikely that children's exposure to the reform increases maternal and paternal survival by more than 0.9 and 2.0 percentage points, respectively, which corresponds to the value of the upper limit of the 95% confidence interval. Our 2SLS estimates suggest that is unlikely that each additional year of schooling increases maternal and paternal survival by more than 2.3 and 4.9 percentage points, respectively. Effects were consistent across a wide array of robustness checks, including alternative specifications of long-run trends, analytical sample, and multiple parental outcomes.

For peer review only - http://bmjopen.bmj.com/site/about/guidelines.xhtml

As with all instruments, a number of assumptions underpin a causal interpretation of these findings. A key assumption underlying our model is that the timing of children's birth around the reform period is random conditional on covariates (no confounding assumption). We controlled for generic time trends in our multivariable models to absorb gradual changes in child and parental characteristics over time. The main logic underlying our identification strategy is that the reform led to an unanticipated *additional* increase in children's schooling among affected cohorts.

There are many reasons why parental socio-economic characteristics and health outcomes might change across children's birth cohorts. Notably, in the early 2000s, Botswana became the first country in sub-Saharan Africa to offer large-scale public treatment for HIV to all qualifying citizens, which had massive and immediate effects on economic and health outcomes (27). Botswana has also implemented some of the most successful drought relief programs in Southern Africa, which were implemented to address the loss of livestock and improve nutritional outcomes (28). These changes in public health prevention programming and socio-economic context in Botswana, however, tend to be gradual over time and/or affect children of different ages. These phenomena would result in gradual changes in parental survival across children's ages and would be picked up in our controls for underlying trends in children's age. The availability of multiple census years also allowed us to generate variation in children's age in a given birth cohort. Our models were therefore robust to period effects (e.g., differences in HIV mortality and health systems across census years), which we controlled for implicitly by simultaneously adjusting for children's age and year of birth.

While the educational reform likely allows us to identify the causal effects of interest, relying on changes generated by the 1996 education reform and census data comes with limitations in terms

For peer review only - http://bmjopen.bmj.com/site/about/guidelines.xhtml

of external validity. First, all of the variation explored reflects variation in secondary schooling. Effects of schooling may be qualitatively and quantitatively different in primary school (29, 30) or higher education (10), and indeed the association between measured schooling and parental survival was non-linear. Second, the causal effects we identify do not necessarily represent the causal effects in the general population but in the subpopulation of compliers (i.e. children who attained increased schooling because of the reform). Nevertheless, one advantage of using Botswana's supply-side school reform is the important size of its impact (nearly half a year of additional schooling on a population level), and that it affected a large segment of the population. Third, educational attainment reported in the census may be subject to measurement error, which may bias the multivariable adjusted estimates towards the null. The instrumental variable analysis, however, is robust to classical (balanced) measurement error in education. Fourth, our measures of parental health may be susceptible to reporting bias. Self-reported health, however, has been suggested to be a valid and reliable indicator when assessing the health of adults in low- and middle-income settings (31). Fifth, parental disability outcomes which are available in the census data, such as visual impairment, are severe health outcomes. Children's education may affect other parental health outcomes or health behaviors, such as smoking and salt consumption. In China, for instance, an education program delivered to school children as part of the formal school curriculum reduced cardiovascular risk factors among their parents (32). In Malawi, a large school-based intervention is currently under way to reduce cardiovascular risk factors in children and their family members (33). Non-communicable diseases, in particular, are expected to pose an increasing challenge in Southern Africa, where health systems have focused on tackling infectious diseases and maternal and child health. Sixth, we observed exposed children only up to age 30 years. Parents' survival and disability may not have been affected during this time period.

For peer review only - http://bmjopen.bmj.com/site/about/guidelines.xhtml

However, this is a common limitation of prevention studies and our analysis captures longer follow-up than randomized controlled trials which observe parental health over a shorter (e.g., 1-2 year) horizon (32). Seventh, the census data only provided information on our secondary parental outcomes for the subset of children living with at least one parent. The effect of children's education on parental disability, for instance, may differ for children and parents who are not living under the same roof. Additionally, selection bias may occur if the decision of children to co-reside with parents depends on exposure to the reform and/or our outcomes for parental disability. However, our estimates for parental disability would only be biased by selection if the reform affected parental disability (i.e., our estimates are likely to be a valid test of the null hypothesis that offspring education does not affect their parents' health, even if there is selection bias) (34). Further investigation is warranted to examine if – and how – involving children in their parents' care may improve the health of the older generation in the region.

Overall, our findings suggest that increasing secondary education of offspring may play a more muted role in the survival and disability of parents than suggested by non-experimental observational and longitudinal studies in LMICs. The lack of parental health gains weakens the evidence base for large "upward" intergenerational benefits of human capital investment.

For peer review only - http://bmjopen.bmj.com/site/about/guidelines.xhtml

References

1. Cutler DM, Lleras-Muney A. Education and Health: Evaluating Theories and Evidence. In: J House, R Schoeni, G Kaplan, Pollack H, editors. Making Americans Healthier: Social and Economic Policy as Health Policy. New York: Russell Sage Foundation; 2008.

2. Karlsson O, De Neve J-W, Subramanian SV. Weakening association of parental education: analysis of child health outcomes in 43 low- and middle-income countries. International Journal of Epidemiology. 2019; 48(1):83-97. doi:10.1093/ije/dyy158

3. Mensch BS, Chuang EK, Melnikas AJ, Psaki SR. Evidence for causal links between education and maternal and child health: systematic review. Tropical Medicine & International Health. 2019; 24(5):504-22. doi:10.1111/tmi.13218

4. Cai F, Giles J, Meng X. How well do children insure parents against low retirement income? An analysis using survey data from urban China. Journal of Public Economics. 2006; 90(12):2229-55. doi:10.1016/j.jpubeco.2006.03.004

5. Bravo J. Intergenerational transfers and social protection in Latin America. National Transfers Accounts Working Paper Series2007.

6. Kohler IV, Kohler HP, Anglewicz P, Behrman JR. Intergenerational transfers in the era of HIV/AIDS: Evidence from rural Malawi. Demogr Res. 2012; 27:775-834.

7. Bloom DE, Canning D, Fink G. Implications of population ageing for economic growth. Oxford Review of Economic Policy. 2011; 26(4):583-612. doi:10.1093/oxrep/grq038

8. Kuziemko I. Human Capital Spillovers in Families: Do Parents Learn from or Lean on Their Children? Journal of Labor Economics. 2014; 32(4):755-86. doi:10.1086/677231

9. Evans R, Becker S. Children caring for parents with HIV and AIDS: global issues and policy responses. Bristol, UK ; Portland, OR: Policy Press; 2009.

10. Chen Y, Persson P, Polyakova M. The Roots of Health Inequality and The Value of Intra-Family Expertise. National Bureau of Economic Research (NBER) Working Paper No. 25618. 2019; [Available at: https://www.nber.org/papers/w25618].

11. Frankenberg E, Lillard L, Willis RJ. Patterns of Intergenerational Transfers in Southeast Asia. Journal of Marriage and Family. 2002; 64(3):627-41. doi:10.1111/j.1741-3737.2002.00627.x

12. World Bank. World Bank national accounts data, and OECD National Accounts data files. [Available at: https://data.worldbank.org/indicator]. 2019. https://data.worldbank.org/indicator. Accessed 18 May 2019.

13. Yang L, Martikainen P, Silventoinen K. Effects of Individual, Spousal, and Offspring Socioeconomic Status on Mortality Among Elderly People in China. Journal of Epidemiology. 2016; 26(11):602-9. doi:10.2188/jea.JE20150252

For peer review only - http://bmjopen.bmj.com/site/about/guidelines.xhtml

14. De Neve J-W, Harling G. Offspring schooling associated with increased parental survival in rural KwaZulu-Natal, South Africa. Social Science & Medicine. 2017; 176:149-57. doi:10.1016/j.socscimed.2017.01.015

15. De Neve J-W, Kawachi I. Spillovers between siblings and from offspring to parents are understudied: A review and future directions for research. Social Science & Medicine. 2017; 183:56-61. doi:10.1016/j.socscimed.2017.04.010

16. Integrated Public Use Microdata Series. Integrated Public Use Microdata Series, International: Version 6.3 [Machine-readable database]. In: University of Minnesota, editor. Minneapolis2014.

17. World Health Organization. Global Health Observatory data repository. In: World Health Organization, editor. Geneva, Switzerland [Available at: https://www.who.int/data/gho]2020.

18. Government of Botswana. The revised national policy on education. Gaborone, Botswana: Botswana Government Printer; 1994.

19. Stangl AL, Lloyd JK, Brady LM, Holland CE, Baral S. A systematic review of interventions to reduce HIV-related stigma and discrimination from 2002 to 2013: how far have we come? Journal of the International AIDS Society. 2013; 16:18734. doi:10.7448/ias.16.3.18734

20. Global Burden of Disease. GBD Results Tool. [Available at: http://ghdx.healthdata.org/gbd-results-tool]. 2020.

21. Axinn WG. The Effects of Children's Schooling on Fertility Limitation. Population Studies. 1993; 47(3):481-93.

22. Hernán MA, Robins JM. Instruments for causal Inference: an epidemiologist's dream? Epidemiology. 2006; 17(4):360-72. doi:10.1097/01.ede.0000222409.00878.37

23. Bor J, Moscoe E, Mutevedzi P, Newell M-L, Bärnighausen T. Regression Discontinuity Designs in Epidemiology. Epidemiology. 2014; 25(5):729-37. doi:10.1097/ede.0000000000000138

24. Davies NM, Thomas KH, Taylor AE, et al. How to compare instrumental variable and conventional regression analyses using negative controls and bias plots. International Journal of Epidemiology. 2017; 46(6):2067-77. doi:10.1093/ije/dyx014

25. Jackson JW, Swanson SA. Toward a Clearer Portrayal of Confounding Bias in Instrumental Variable Applications. Epidemiology. 2015; 26(4):498-504. doi:10.1097/ede.0000000000000287

26. Angrist JD, Imbens GW, Rubin DB. Identification of Causal Effects Using Instrumental Variables. Journal of the American Statistical Association. 1996; 91(434):444. doi:10.2307/2291629

27. Bussmann H, Wester CW, Ndwapi N, et al. Five-year outcomes of initial patients treated in Botswana's National Antiretroviral Treatment Program. Aids. 2008; 22(17):2303-11. doi:10.1097/QAD.0b013e3283129db0

28. Belbase K, Morgan R. Food security and nutrition monitoring for drought relief management: the case of Botswana. Food Policy. 1994; 19(3):285-300. doi:10.1016/0306-9192(94)90076-0

For peer review only - http://bmjopen.bmj.com/site/about/guidelines.xhtml

29. De Neve J-W, Fink G. Children's education and parental old age survival – Quasi-experimental evidence on the intergenerational effects of human capital investment. Journal of Health Economics. 2018; 58:76-89. doi:10.1016/j.jhealeco.2018.01.008

30. Ma M. Does children's education matter for parents' health and cognition? Evidence from China. Journal of Health Economics. 2019; 66:222-40. doi:10.1016/j.jhealeco.2019.06.004

31. Subramanian SV, Subramanyam MA, Selvaraj S, Kawachi I. Are self-reports of health and morbidities in developing countries misleading? Evidence from India. Soc Sci Med. 2009; 68(2):260-5. doi:10.1016/j.socscimed.2008.10.017

32. He FJ, Wu Y, Feng XX, et al. School based education programme to reduce salt intake in children and their families (School-EduSalt): cluster randomised controlled trial. Bmj. 2015; 350(mar18 13):h770-h. doi:10.1136/bmj.h770

33. UK Research and Innovation. NoToNa: Tackling cardiovascular risk in the adolescent life-course through a schools' salt-reduction intervention in sub-Saharan Africa. 2020. https://gtr.ukri.org/projects?ref=MR%2FR022186%2F1. Accessed 16 Jan 2020.

34. Hughes RA, Davies NM, Davey Smith G, Tilling K. Selection Bias When Estimating Average Treatment Effects Using One-sample Instrumental Variable Analysis. Epidemiology. 2019; 30(3):350-7. doi:10.1097/ede.0000000000000972

For peer review only

TABLES

Table 1. Selected characteristics of estimation sample.

	Pre-reform cohorts (n = 34,202)		Post-reform cohorts (n = 55,519)	
	**Census 2001**	**Census 2011**	**Census 2001**	**Census 2011**
Children's characteristics				
Mother alive, %	88.5	77.5	90.9	81.1
Father alive, %	71.3	50.7	77.6	63.2
Age, mean (SD)	23.4 (1.7)	33.4 (1.7)	18.9 (0.8)	23.8 (3.8)
Years of schooling, mean (SD)	8.9 (3.4)	9.5 (4.0)	9.3 (3.0)	10.4 (3.3)
Has at least ten years of schooling, %	42.0	45.3	65.1	77.1
Labor force participation, %	65.3	83.6	37.3	65.0
Speaks Setswana at home, %	82.5	83.8	82.0	81.9
Christian, %	70.2	79.0	71.8	78.0
Household characteristics				
Number of family members in household, mean (SD)	5.2 (3.9)	4.5 (3.5)	6.1 (3.8)	4.8 (3.7)
Any deaths in household last year, %	5.7	2.7	6.4	2.9
Ownership of dwelling, %	63.0	57.1	74.6	62.4
Access to electricity, %	24.6	57.2	23.4	55.4
Access to piped water, %	19.5	31.0	18.5	28.2
Telephone availability, %	39.1	10.4	36.6	11.4
Cellular phone availability, %	-	94.2	-	93.9
Receives remittances, %	21.5	30.4	23.1	32.1

Sample includes survey respondents who were citizens of Botswana, born in Botswana, at least 18 years old at the time of the census, and born in or after 1975. The unit of analysis was children born either during the pre-reform period (prior to 1981) or post-reform period (in or after 1981). SD=Standard Deviation. Source: Botswana Census 2001 and 2011.

For peer review only - http://bmjopen.bmj.com/site/about/guidelines.xhtml

Table 2. First stage results: effect of the reform on children's educational attainment.

Subsample	**Daughters**	**Sons**	**Both sexes**
Dependent variable			
Child's schooling (years)	*Difference in years of schooling (95% CI)*		
Reform indicator	0.458***	0.365***	0.414***
	(0.330, 0.586)	(0.210, 0.521)	(0.314, 0.514)
Observations	47,121	42,600	89,721
R-squared	0.088	0.080	0.088
Child has ≥ 10 years of schooling (1=yes, 0=no)	*Difference in Pr(Educ ≥ 10) (95% CI)*		
Reform indicator	22.3***	15.4***	19.0***
	(20.3, 24.2)	(13.3, 17.5)	(17.5, 20.4)
Observations	47,121	42,600	89,721
R-squared	0.152	0.118	0.136

Notes: Regression results from ordinary least squares models controlling for children's single-year age indicators, a continuous trend in children's year of birth, and children's district of birth. Regressions for the subsample with both children's sexes additionally control for an indicator for children's sex and interactions of each covariate with children's sex. Our models are robust to period effects, which we controlled for implicitly by simultaneously adjusting for children's age and year of birth. Children's exposure to the reform was defined as a binary 'Reform indicator' (1=year of birth > 1980; 0=otherwise). Pr (Educ ≥ 10) was defined at the probability of a child completing at least ten years of schooling by the time of the census (1=yes, 0=no). The sample includes survey respondents who were citizens born in Botswana, at least 18 years old at the time of the census, and born in or after 1975. 95% robust confidence intervals in parentheses. Binary outcomes were multiplied by 100 to facilitate the interpretation of coefficients and standard errors on a % point scale. *** p<0.01, ** p<0.05, * p<0.1. Source: Botswana Census 2001 and 2011.

For peer review only - http://bmjopen.bmj.com/site/about/guidelines.xhtml

Table 3. The association of children's schooling and parental survival.

Dependent variable	Mother alive (1=yes, 0=no)			Father alive (1=yes, 0=no)		
Subsample	Daughters	Sons	Both sexes	Daughters	Sons	Both sexes
			Risk difference (95% CI)			
A: OLS model						
Child's schooling (years)	0.9***	0.7***	0.8***	0.8***	0.5***	0.6***
	(0.8, 1.0)	(0.6, 0.8)	(0.7, 0.9)	(0.6, 0.9)	(0.4, 0.6)	(0.5, 0.7)
B: OLS model						
Child has ≥ 10 years of schooling (1=yes, 0=no)	4.6***	3.7***	4.1***	4.0***	1.9***	3.0***
	(3.8, 5.4)	(2.9, 4.5)	(3.6, 4.7)	(3.0, 4.9)	(0.9, 2.9)	(2.3, 3.7)
C: ITT model						
Reform indicator	-0.5	0.2	-0.2	0.4	0.7	0.5
	(-2.1, 1.0)	(-1.4, 1.8)	(-1.3, 0.9)	(-1.6, 2.5)	(-1.5, 2.8)	(-0.9, 2.0)
D: 2SLS model						
Child's schooling (years)	-1.2	0.6	-0.4	0.9	1.8	1.3
	(-4.6, 2.3)	(-3.9, 5.0)	(-3.2, 2.3)	(-3.5, 5.4)	(-4.1, 7.7)	(-2.3, 4.9)
F-statistic	49.0	21.1	65.6	49.0	21.1	65.6
Dependent variable, pre-reform (%)	83.3	83.4	83.4	61.3	62.4	61.8
Observations	47,121	42,600	89,721	47,121	42,600	89,721

Notes: **Panels A** and **B** show regression results from an ordinary least squares (OLS) model controlling for children's single-year age indicators, a continuous trend in children's year of birth, and children's district of birth. Regressions for the subsample with both children's sexes additionally control for an indicator for children's sex and interactions of each covariate with children's sex. Our models are robust to period effects, which we controlled for implicitly by simultaneously adjusting for children's age and year of birth. 'Child has ≥ 10 years of schooling' was defined at the probability of a child completing at least ten years of schooling by the time of the census (1=yes, 0=no). **Panel C** shows regression results from an intention-to-treat (ITT) OLS model in which children's exposure to the reform was defined as a binary 'Reform indicator' (1=year of birth > 1980; 0=otherwise). **Panel D** shows regression results from a two-stage least squares (2SLS) model, in which exposure to the reform was used as an instrument for children's years of schooling. Sample includes survey respondents who were citizens born in Botswana, at least 18 years old at the time of the census, and born in or after 1975. Binary outcomes were multiplied by 100 to facilitate the interpretation of coefficients and standard errors on a % point scale. 95% robust confidence intervals (CI) in parentheses. *** p<0.01, ** p<0.05, * p<0.1. Source: Botswana Census 2001 and 2011.

For peer review only - http://bmjopen.bmj.com/site/about/guidelines.xhtml

Table 4. The association of children's schooling and secondary parental outcomes.

Dependent variable ***Sample: both sexes***	**Number of children ever born to mother**	**Age of mother's youngest child in household (years)**	**Maternal labor force participation (1=yes, 0=no)**	**Parental employment disability (1=yes, 0=no)**	**Parental blindness (1=yes, 0=no)**	**Parental lower extremity disability (1=yes, 0=no)**
A: OLS model						
Child's schooling (years)	-0.2***	0.1***	1.6***	-0.5***	-0.1**	-0.1***
	(-0.2, -0.1)	(0.0, 0.1)	(1.4, 1.7)	(-0.6, -0.4)	(-0.2, -0.0)	(-0.1, -0.0)
B: OLS model						
Child has ≥ 10 years of schooling (1=yes, 0=no)	-0.7***	0.3***	8.5***	-2.2***	-0.5**	-0.5***
	(-0.8, -0.6)	(0.1, 0.5)	(7.2, 9.7)	(-2.8, -1.6)	(-0.9, -0.0)	(-0.8, -0.2)
C: ITT model						
Reform indicator	0.0	0.3	-2.1	0.3	0.0	0.1
	(-0.1, 0.1)	(-0.1, 0.7)	(-4.5, 0.7)	(-0.7, 1.4)	(-0.8, 0.8)	(-0.4, 0.7)
D: 2SLS model						
Child's schooling (years)	0.0	0.7	-4.8	0.8	0.0	0.3
	(-0.3, 0.3)	(-0.2, 1.6)	(-10.5, 2.0)	(-1.5, 3.1)	(-1.8, 1.8)	(-0.8, 1.5)
F-statistic	29.8	29.8	29.8	33.5	33.4	33.4
Probability dependent variable, pre-reform	6.3	17.8	38.8	6.3	3.8	1.6
Observations	26,664	26,664	26,664	29,212	29,212	29,212

Notes: **Panels A** and **B** show regression results from an ordinary least squares (OLS) model controlling for children's single-year age indicators, a continuous trend in children's year of birth, children's district of birth, an indicator for children's sex, and the interactions of each covariate with children's sex. Our models are robust to period effects, which we controlled for implicitly by simultaneously adjusting for children's age and year of birth. 'Child has ≥ 10 years of schooling' was defined at the probability of a child completing at least ten years of schooling by the time of the census (1=yes, 0=no). **Panel C** shows regression results from an intention-to-treat (ITT) OLS model in which exposure to the reform was defined as a binary indicator (1=year of birth > 1980; 0=otherwise). **Panel D** shows regression results from a two-stage least squares (2SLS) model, in which exposure to the reform was used as an instrument for years of schooling. Sample includes survey respondents who were citizens born in Botswana, at least 18 years old at the time of the census, born in or after 1975, and co-resided with their mother (columns 2-4) or at least one parent (columns 5-7) at the time of the census. Binary outcomes were multiplied by 100 to facilitate the interpretation of coefficients and standard errors on a % point scale. 95% robust confidence intervals in parentheses. *** p<0.01, ** p<0.05, * p<0.1. Source: Botswana Census 2001 and 2011.

For peer review only - http://bmjopen.bmj.com/site/about/guidelines.xhtml

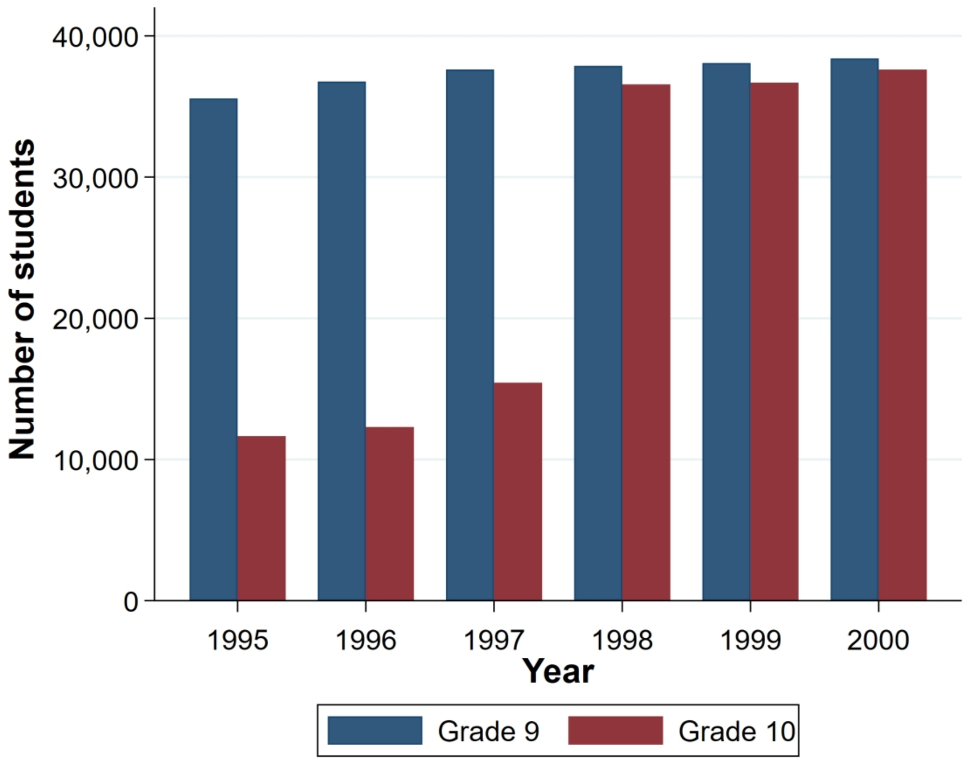

Figure 1. Enrollment in grade 9 and 10 in Botswana by year. Figure 1 shows total enrollment in grade 9 and 10 in Botswana by year. In January 1996, Botswana shifted grade 10 from senior secondary to junior secondary school, with the goal of increasing access. Botswana's education system is highly centralized and the reform was implemented rapidly throughout the country. More teachers were hired and classrooms were created. Moving grade 10 to junior secondary reduced travel time for students in addition to expanding the number of grade-10 seats. The reform also raised the benefits of completing grade 10 because it was now required to obtain a Junior Certificate. The result of this reform was a large increase in the number of students attending grade 10 from 1997 to 1998. Source: Botswana Education Statistics 1995 – 2000.

173x141mm (300 x 300 DPI)

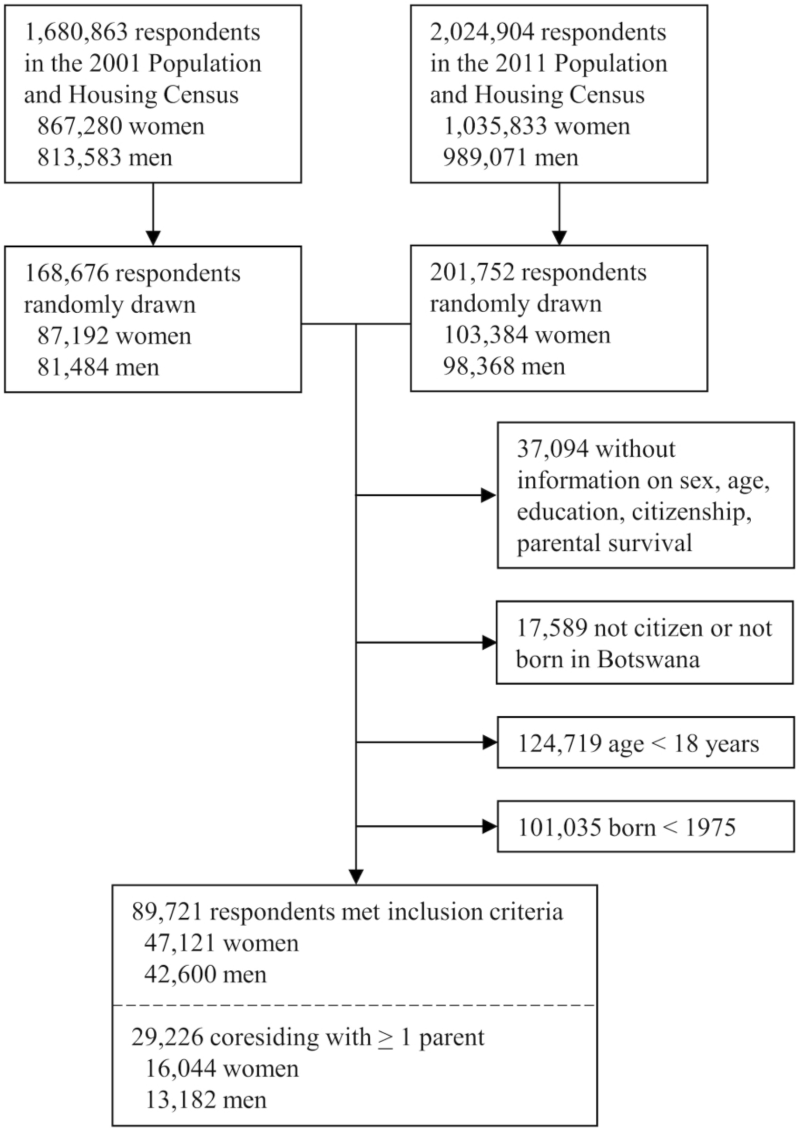

Figure 2. Study participant flow diagram. Figure 2 shows the flow of participants through the Botswana Census of 2001 and 2011.

163x222mm (300 x 300 DPI)

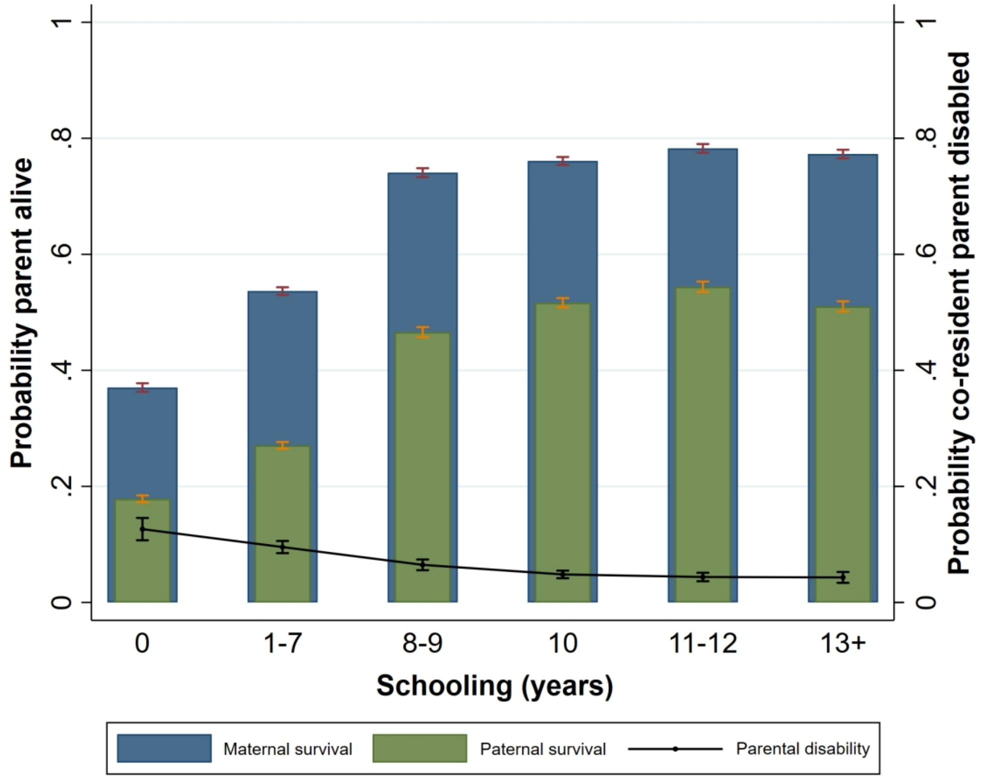

Figure 3. Parental survival and disability by children's educational attainment. Figure 3 shows the unadjusted relationship between children's schooling and parental survival status and disability. Educational attainment was defined as total years of schooling completed by the time of the census. Strong protective effects are seen for primary school completion, with effects levelling off after junior secondary school. The sample for parental survival includes respondents who are Botswana citizens, born in Botswana, and ages at least 22 at the time of the census. The sample for parental disability includes respondents who are Botswana citizens, born in Botswana, ages at least 22, and living with at least one parent at the time of the census. Source: Botswana Census 2011.

173x141mm (300 x 300 DPI)

Supplementary webappendix

- Text S1
- Figures S1-S5
- Tables S1-S7

For peer review only

For peer review only - http://bmjopen.bmj.com/site/about/guidelines.xhtml

Text S1. Study context and education policy reform

Botswana is a sparsely-populated, land-locked, middle-income country in southern Africa, with a rapidly growing economy which is highly dependent on diamond-mining. Botswana has among the world's highest HIV prevalence rates, and with the scale-up of HIV treatment, life expectancy at birth increased from 48.1 years to 61.9 years between 2001 and 2011 (World Health Organization 2020).

<u>Botswana education system and policy reform</u>

Botswana's public education system is based on the "7-3-2" system, including seven years of primary school, three years of junior secondary school, and two years of senior secondary school. In January 1996, the Government of Botswana shifted grade 10 from senior secondary school to junior secondary school, as part of implementing the country's Revised National Policy on Education (Government of Botswana 1994). Botswana's National Commission on Education had criticized the former "7-2-3" system for not adequately preparing students for the labor market. Botswana's public education system is strongly centralized so that the policy was implemented rapidly and universally. Moreover, the vast majority of the population attends public schools with less than 1% of secondary school students in private schools (Statistics Botswana 2013). More teachers were hired, and classrooms were created. Travel times were reduced, and the number of grade 10 seats increased dramatically since there were many more junior secondary than senior secondary schools during the pre-reform period. The schooling reform also raised the benefits of completing grade 10 because it was now required to obtain a "Junior Certificate".

The result of the reform was a very large increase in the number of students attending grade 10 from 1997 to 1998 when the first cohorts were affected (i.e., those starting junior secondary school in 1996). Previous studies have found that the reform led to a large increase in schooling attainment for affected cohorts (Borkum 2009; De Neve et al. 2015; Lindskog and Durevall 2019) (**Figure S1**). In addition to schooling attainment, the reform improved children's health outcomes (e.g., sexual and reproductive health) (Lindskog and Durevall 2019); economic outcomes (e.g., labor force participation) (Borkum 2009); skills; and health services utilization (e.g., HIV testing) (Bor and De Neve 2015). This natural experiment provides an opportunity to estimate the causal impact of schooling on parental outcomes, by comparing parents of birth cohorts exposed to the reform versus those unexposed.

For peer review only - http://bmjopen.bmj.com/site/about/guidelines.xhtml

Alternative hypotheses possibly affecting post-1980 birth cohorts

First, we consulted widely with scholars and government officials in Botswana to understand how the education policy reform was implemented and competing events that may have differentially affected post-1980 cohorts. Key stakeholders were identified through snowball sampling, and included staff from the Ministry of Health (n=2), Statistics Botswana (Central Statistics Botswana) (n=3), University of Botswana's Department of Economics (n=1), University of Botswana's Department of Education (n=2), the Botswana Harvard Partnership (n=2), as well as a teacher who worked in Botswana during the time of the 1996 reform (n=1). Interview questions covered the implementation of the 1996 reform; institutional and historical context; and any competing events. We asked for other social reforms or population changes that might have happened around the time and potentially have affected children born after 1980.

Second, we conducted a desk review of both published and unpublished documents. These included the Revised National Policy of Education (1994), the Revised National Population Policy (1997), the Seventh National Development Plan (1991-1997), as well as research and review articles on Botswana's education system and curriculum. In **Table S1**, we list alternative policy hypotheses with a brief description of each policy and whether or not these could be a concern for our main identification strategy. Potential concerns included, for instance, the roll-out of socio-economic reforms, vaccine or disaster relief programs, and/or HIV treatment and prevention programs if these affected specific children's birth cohorts.

For peer review only

Figure S1. Children's schooling by year of birth in Botswana

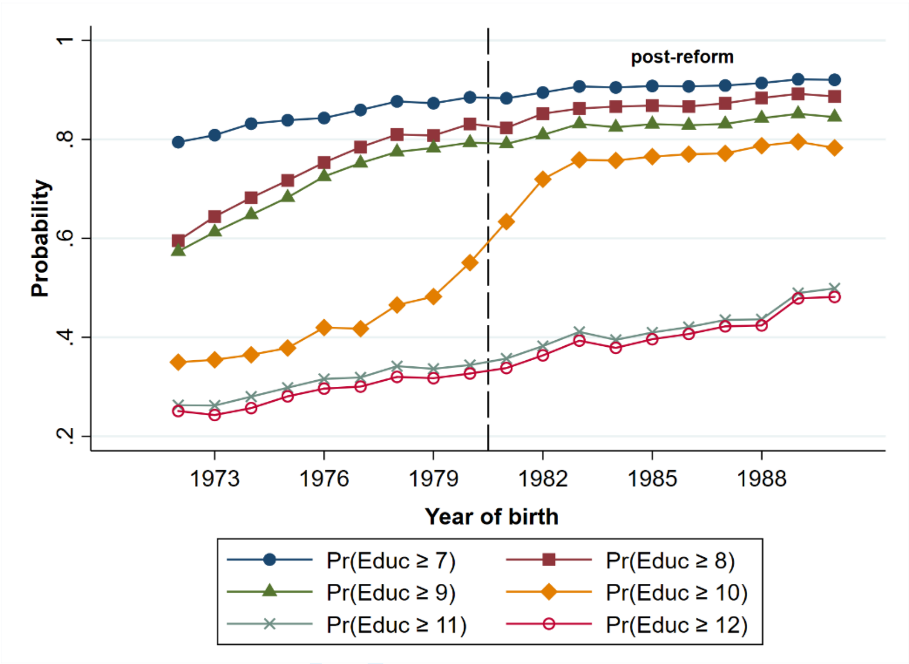

Notes: Figure replicates findings from De Neve et al., 2015 using data from the Botswana Census 2011 (*N*=108,092). The figure shows the probability that a child has attained at least X years of schooling by the time of the census. The sample includes survey respondents who were citizens of Botswana, born in Botswana and ages at least 18 years at the time of the census. Individuals born in 1981 or later (dashed line) would have entered junior secondary school in 1996 or later, and were thus classified as exposed to the education reform.

For peer review only - http://bmjopen.bmj.com/site/about/guidelines.xhtml

Figure S2. Assumptions underpinning causal inference

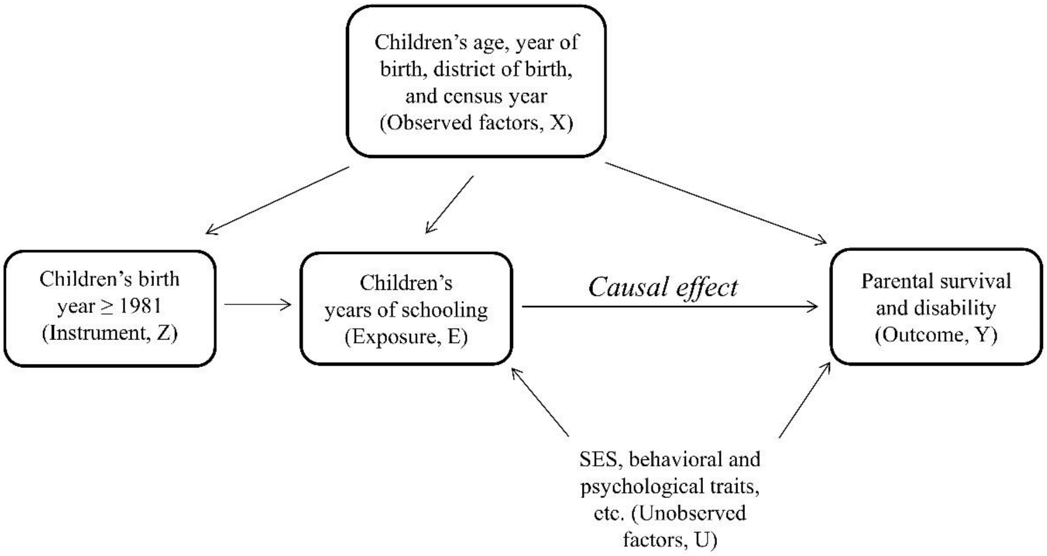

Notes: Directed acyclic graph illustrating the instrumental variable assumptions for causal interpretation. Conditional on *X*, *Z* is a valid instrument if *Z* causally affects *E*, *Z* is uncorrelated with *U*, and *Z* affects *Y* only through *E*. Under the assumption that *Z* only affects *E* in one direction, the instrumental variable estimators estimate a local average treatment effect.

For peer review only - http://bmjopen.bmj.com/site/about/guidelines.xhtml

Figure S3. Cohort sizes

(a) Census 2001

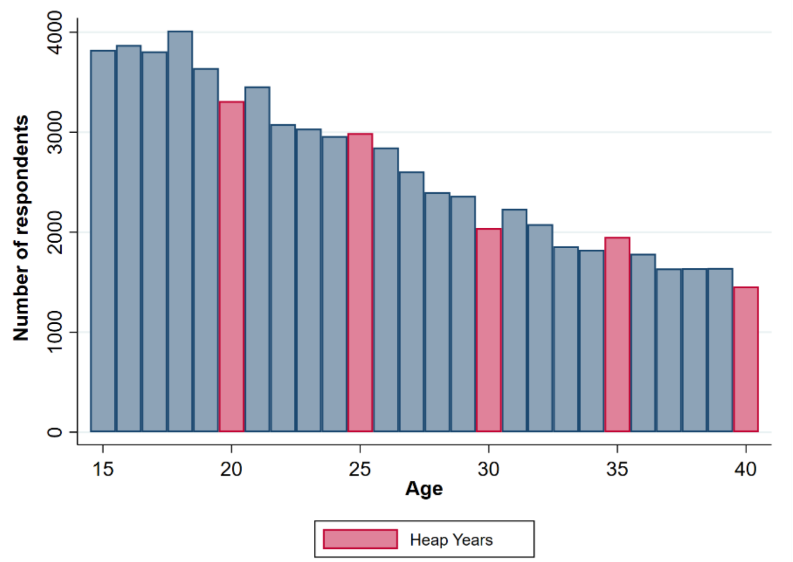

(b) Census 2011

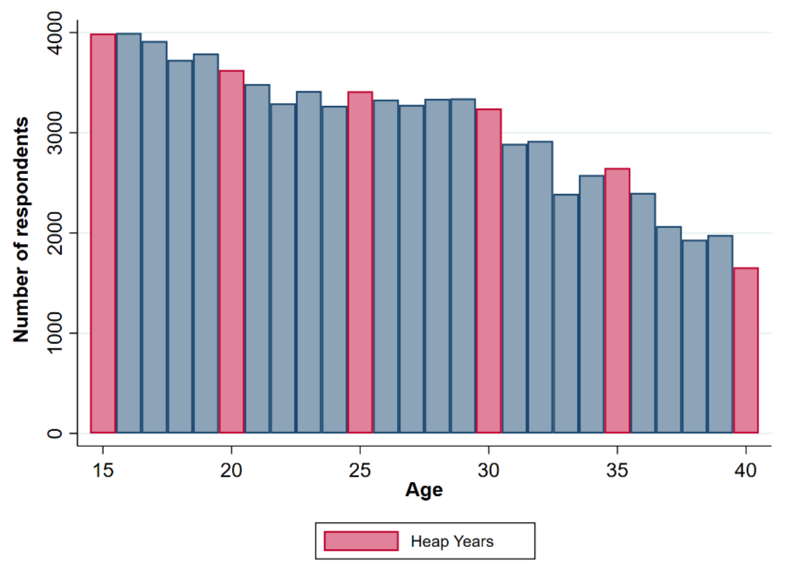

Notes: Figure shows the number of respondents by age (birth cohort), separately for each census wave. Respondents affected by the education policy reform were ages $\leq$ 20 (census 2001) or $\leq$ 30 (census 2011). Source: Botswana Census 2001 and 2011.

For peer review only - http://bmjopen.bmj.com/site/about/guidelines.xhtml

Figure S4. Parental survival and disability by children's year of birth

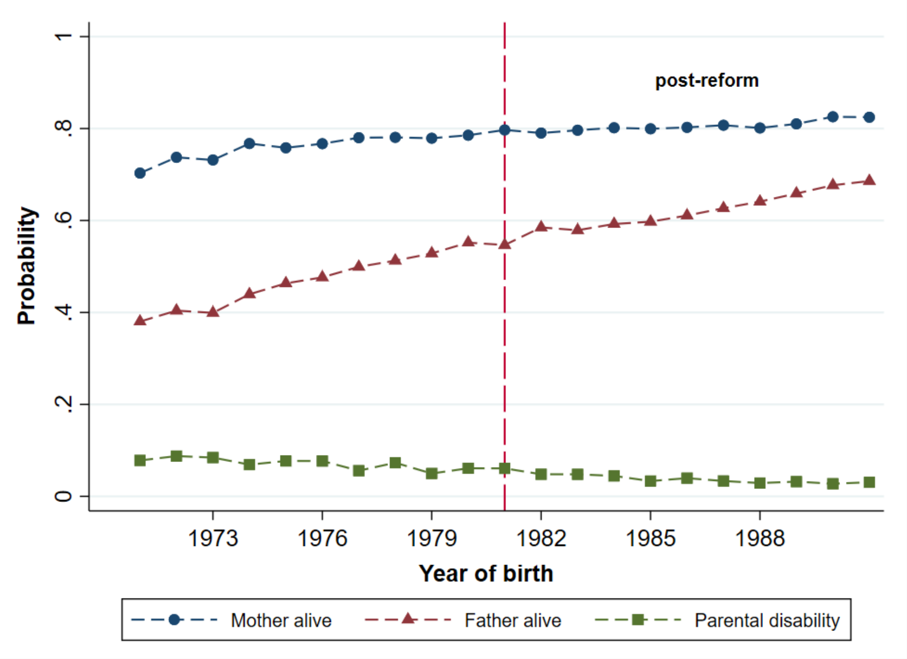

Notes: Figure shows survival of mothers (blue) and fathers (red) as well as parental disability (green) by children's birth cohort. The vertical dashed line indicates the timing of the policy reform. Parental disability was defined as any parental disability among the surviving mother and/or father. The sample for parental survival includes survey respondents who were citizens of Botswana and born in Botswana between 1971 and 1991; whereas the sample for disability additional includes only those children living with at least one parent. Source: Botswana Census 2011.

Figure S5. Bias component plots

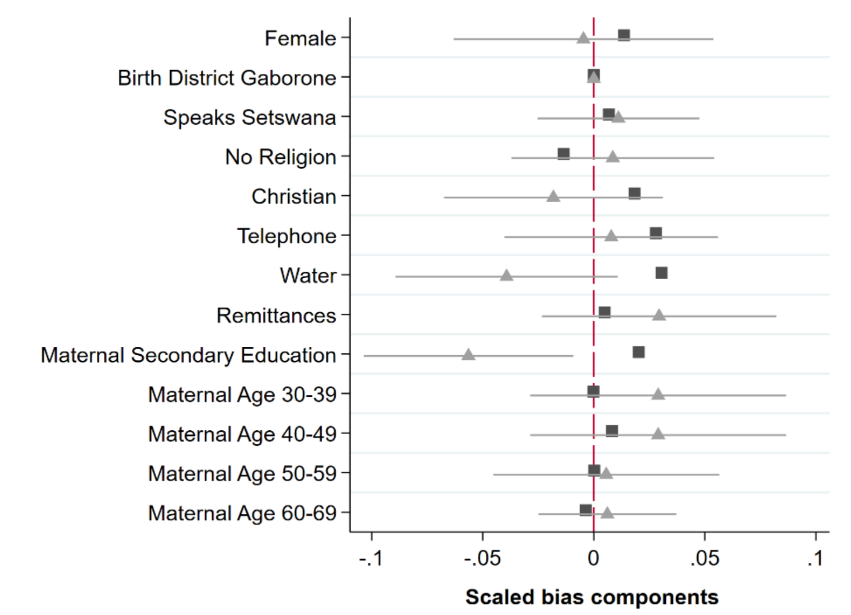

Notes: Bias component plots for the difference in children's sex, birth district, language, religion, household assets, household remittances, parental education, and parental age, by actual exposure (squares) and proposed instrument (triangles). The figures for the instrumental variable results account for the strength of the instrument as described in Jackson and Swanson (2015) and Davies (2017). All variables are binary. The horizontal lines indicate robust confidence intervals for years of schooling. Sample includes survey respondents who were citizens born in Botswana, at least 18 years old at the time of the census, born in or after 1975, and co-resided with at least one parent at the time of the census. Source: Botswana Census 2001 and 2011.

For peer review only - http://bmjopen.bmj.com/site/about/guidelines.xhtml

Table S1. Alternative Hypotheses Possibly Affecting Post-1980 Birth Cohorts.

**Alternative hypothesis**	**Policy (year) and description**	**Concern or not**
Curriculum changes	• Revised National Policy of Education (1994): increase the vocational orientation of academic subjects, increase the number of practical subjects, emphasize professional skills, relate the curriculum to the professional environment, and increase career guidance.	Not implemented nationally; gradual implementation over at least five years.
National ART program	• National ART Program (2002): ART became available free of charge through public health services. Aimed to deliver care with a high level of clinical monitoring and a low tolerance of adverse events, as in high-resource settings.	Rolled out later and/or did not affect specific birth cohorts.
Home based care, Prevention of Mother to Child Transmission, HIV testing policies	• Community Home Based Care (1995): ensure continuity of comprehensive care services and social support to AIDS patients.<br>• Prevention of Mother to Child Transmission (1999): available in all public health facilities.<br>• HIV testing (2000): introduction of voluntary counselling and testing centers.	Rolled out later and did not affect specific birth cohorts.
Abortion policy change	• The Penal Code Amendment Act of 11 October 1991 (1991): abortion was de-criminalized within the first 16 weeks of pregnancy under certain circumstances.	Implemented earlier and did not affect specific birth cohorts.
Family planning changes	• National Population Policy (1997): improve quality of life and standard of living of all people; through reduced population growth rate, low fertility, low morbidity and mortality, and a balanced population distribution.	Proposed almost two years after the 1996 reform (August 1997); unlikely to have affected specific birth cohorts.
Socio-economic reforms	• National Development Plan Number (1991-1997): outline short to medium-term development initiatives for Botswana	Implemented gradually over many years.
Drought relief program	• Botswana's drought relief program (1982-1990): address the loss of livestock and malnutrition, particularly among children sufficient food.	Implemented gradually over many years.

Abbreviation: ART: anti-retroviral therapy

For peer review only - http://bmjopen.bmj.com/site/about/guidelines.xhtml

Table S2. Descriptive statistics: subset of children living with at least one parent (N=29,226)

Subsample: children co-residing with ≥ 1 parent	**Pre-reform cohorts (n = 9,467)**		**Post-reform cohorts (n = 19,759)**	
	**Census 2001**	**Census 2011**	**Census 2001**	**Census 2011**
Children's characteristics				
Any parental disability, %	5.7	6.4	4.8	3.5
Age, mean (SD)	23.1 (1.7)	33.3 (1.7)	18.9 (0.8)	23.1 (3.7)
Years of schooling, mean (SD)	9.1 (3.3)	9.1 (3.6)	9.5 (2.8)	10.4 (3.1)
Has at least ten years of schooling, %	45.0	42.1	68.2	78.0
Labor force participation, %	58.2	78.2	32.4	59.8
Speaks Setswana at home, %	84.5	86.6	83.0	83.0
Christian, %	71.8	77.4	73.4	79.3
Household characteristics				
Number of family members in household, mean (SD)	8.0 (3.6)	7.7 (3.8)	7.6 (3.5)	7.1 (3.5)
Any deaths in household last year, %	7.6	5.1	6.9	4.0
Ownership of dwelling, %	90.0	94.3	87.0	86.3
Access to electricity, %	20.9	53.6	21.1	53.2
Access to piped water, %	14.6	21.1	16.1	25.1
Telephone availability, %	37.3	18.6	35.8	16.9
Cellular phone availability, %	-	95.0	-	95.1
Receives remittances, %	24.1	35.0	24.0	32.9

Sample includes survey respondents who were citizens of Botswana, born in Botswana, at least 18 years old at the time of the census, born in or after 1975, and who co-resided with at least one parent at the time of the census. The unit of analysis was children born either during the pre-reform period (prior to 1981) or post-reform period (in or after 1981). SD=Standard Deviation. Source: Botswana Census 2001 and 2011.

For peer review only

For peer review only - http://bmjopen.bmj.com/site/about/guidelines.xhtml

Table S3. OLS and ITT results controlling for parental socio-demographic characteristics

Dependent variable	**Parental disability (1=yes, 0=no)**		
Subsample	**Daughters**	**Sons**	**Both sexes**
	Risk difference (95% CI)		
A: OLS model			
Schooling (years)	-0.1*	-0.1	-0.1**
	(-0.2, 0.0)	(-0.2, 0.0)	(-0.2, -0.0)
B: OLS model			
≥ 10 years of schooling (1=yes,	-0.5	-1.0**	-0.7**
	(-1.3, 0.3)	(-1.9, -0.1)	(-1.3, -0.2)
C: ITT model			
Reform indicator	-0.3	-1.1	-0.7
	(-1.8, 1.1)	(-2.7, 0.5)	(-1.8, 0.4)
Additional controls			
Maternal age (years)	✓	✓	✓
Maternal schooling (years)	✓	✓	✓
Observations	15,006	12,127	27,133

Notes: Panels A and B show regression results from 'conventional' multivariable OLS models controlling for single-year age indicators, a continuous trend in year of birth, district of birth, as well as maternal age and maternal educational attainment (years of schooling completed). Regressions for the subsample with both children's sexes additionally control for an indicator for children's sex and interactions of each covariate with children's sex. Our models are robust to period effects, which we controlled for implicitly by simultaneously adjusting for children's age and year of birth. Panel C shows regression results from an ITT model (OLS), in which exposure to the reform was defined as a binary indicator (1=year of birth > 1980; 0=otherwise). The sample includes survey respondents who were citizens born in Botswana, at least 18 years old at the time of the census, born in or after 1975, and co-resided with at least their mother at the time of the census. Binary outcomes were multiplied by 100 to facilitate the interpretation of coefficients and standard errors on a % point scale. 95% robust confidence intervals in parentheses. *** p<0.01, ** p<0.05, * p<0.1. Source: Botswana Census 2001 and 2011.

For peer review only - http://bmjopen.bmj.com/site/about/guidelines.xhtml

Table S4. ITT results: testing the robustness of our findings to alternative specifications in children's age, YOB, additional controls for heap year, slope change in YOB, birth cohort windows, and analytical sample (outcome: maternal survival)

Dependent variable: mother alive (1=yes, 0=no) ***Sample: both sexes*** ***Model: ITT***	**(1)**	**(2)**	**(3)**	**(4)**	**(5)**	**(6)**	**(7)**	**(8)**	**(9)**	**(10)**	**(11)**	**(12)**
Coefficient on endogenous variable (child)												
Reform indicator	-0.2	-0.2	0.1	0.4	0.8	0.6	-0.1	-0.2	-0.6	-0.6	0.7	-0.0
	(0.6)	(1.0)	(0.5)	(0.4)	(0.7)	(0.6)	(0.4)	(0.6)	(1.0)	(0.6)	(0.9)	(1.2)
Basic covariates (child)												
i.Age##i.Sex	✓	✓	-	-	-	-	-	✓	✓	✓	✓	✓
c.YOB##i.Sex	✓	✓	✓	✓	✓	✓	✓	✓	✓	✓	✓	✓
i.CensusYear##i.Sex	✓	✓	✓	✓	✓	✓	✓	✓	✓	✓	✓	✓
i.BirthDistrictr##i.Sex	✓	✓	✓	✓	✓	✓	✓	✓	✓	✓	✓	✓
Additional controls (child)												
c.YOB2##i.Sex	-	✓	-	-	-	-	-	-	-	-	-	-
i.ThreeYearAgeGroup##i.Sex	-	-	✓	-	-	-	-	-	-	-	-	-
c.Age##i.Sex	-	-	-	✓	✓	✓	✓	-	-	-	-	-
c.Age2##i.Sex	-	-	-	✓	✓	✓	✓	-	-	-	-	-
c.Age3##i.Sex	-	-	-	✓	-	-	-	-	-	-	-	-
c.Age4##i.Sex	-	-	-	✓	-	-	-	-	-	-	-	-
i.HeapYear##i.Sex	-	-	-	-	-	-	-	✓	-	-	-	-
c.YOB#i.Reform_Indicator, c.YOB#i.Reform_Indicator#i.Sex	-	-	-	-	-	-	-	-	✓	-	-	-
Birth cohort windows (child)												
Narrower birth cohort window, 1978 ≤ YOB ≤ 1984	-	-	-	-	✓	-	-	-	-	-	-	-
Narrower birth cohort window, 1976 ≤ YOB ≤ 1986	-	-	-	-	-	✓	-	-	-	-	-	-
Earlier birth cohorts included, 1971 ≤ YOB ≤ 1993	-	-	-	-	-	-	✓	-	-	-	-	-
Subsample, children with ≥ 9 years of schooling	-	-	-	-	-	-	-	-	-	✓	-	-
Subsample, co-resides with parent and gap ≥ 5.5 years of schooling	-	-	-	-	-	-	-	-	-	-	✓	-
Subsample, co-resides with parent and paternal age ≥ 50 years	-	-	-	-	-	-	-	-	-	-	-	✓
Observations	89,721	89,721	89,721	89,721	41,942	59,866	106,774	89,721	89,721	71,608	14,547	9,691

Notes: Robustness checks for the intention-to-treat (ITT) regression results shown in column 4 in Table 3 in the main text (maternal survival). The sample in models 1–4 and models 8–12 includes survey respondents who were citizens born in Botswana, at least 18 years old at the time of the census, and born in or after 1975. Binary outcomes were multiplied by 100 to facilitate the interpretation of coefficients and standard errors on a % point scale. Robust standard errors in parentheses. *** p<0.01, ** p<0.05, * p<0.1. Source: Botswana Census 2001 and 2011. YOB=year of birth.

Table S5. ITT results: testing the robustness of our findings to alternative specifications in children's age, YOB, additional controls for heap year, slope change in YOB, birth cohort windows, and analytical sample (outcome: paternal survival)

Dependent variable: father alive (1=yes, 0=no) ***Sample: both sexes*** ***Model: ITT***	**(1)**	**(2)**	**(3)**	**(4)**	**(5)**	**(6)**	**(7)**	**(8)**	**(9)**	**(10)**	**(11)**	**(12)**
Coefficient on endogenous variable (child)												
Reform indicator	0.5	1.3	0.4	0.7	0.5	0.1	-0.2	-0.2	1.0	0.0	0.7	-0.5
	(0.8)	(1.4)	(0.7)	(0.6)	(0.9)	(0.7)	(0.5)	(0.6)	(1.2)	(0.8)	(0.9)	(2.1)
Basic covariates (child)												
i.Age##i.Sex	✓	✓	-	-	-	-	-	✓	✓	✓	✓	✓
c.YOB##i.Sex	✓	✓	✓	✓	✓	✓	✓	✓	✓	✓	✓	✓
i.CensusYear##i.Sex	✓	✓	✓	✓	✓	✓	✓	✓	✓	✓	✓	✓
i.BirthDistrictr##i.Sex	✓	✓	✓	✓	✓	✓	✓	✓	✓	✓	✓	✓
Additional controls (child)												
c.YOB2##i.Sex	-	✓	-	-	-	-	-	-	-	-	-	-
i.ThreeYearAgeGroup##i.Sex	-	-	✓	-	-	-	-	-	-	-	-	-
c.Age##i.Sex	-	-	-	✓	✓	✓	✓	-	-	-	-	-
c.Age2##i.Sex	-	-	-	✓	✓	✓	✓	-	-	-	-	-
c.Age3##i.Sex	-	-	-	✓	-	-	-	-	-	-	-	-
c.Age4##i.Sex	-	-	-	✓	-	-	-	-	-	-	-	-
i.HeapYear##i.Sex	-	-	-	-	-	-	-	✓	-	-	-	-
c.YOB#i.Reform_Indicator, c.YOB#i.Reform_Indicator#i.Sex	-	-	-	-	-	-	-	-	✓	-	-	-
Birth cohort windows (child)												
Narrower birth cohort window, 1978 ≤ YOB ≤ 1984	-	-	-	-	✓	-	-	-	-	-	-	-
Narrower birth cohort window, 1976 ≤ YOB ≤ 1986	-	-	-	-	-	✓	-	-	-			
Earlier birth cohorts included, 1971 ≤ YOB ≤ 1993	-	-	-	-	-	-	✓	-	-	-	-	-
Subsample, children with ≥ 9 years of schooling	-	-	-	-	-	-	-	-	-	✓	-	-
Subsample, co-resides with parent and gap ≥ 5.5 years of schooling	-	-	-	-	-	-	-	-	-	-	✓	-
Subsample, co-resides with parent and maternal age ≥ 50 years	-	-	-	-	-	-	-	-	-	-	-	✓
Observations	89,721	89,721	89,721	89,721	41,942	59,866	106,774	89,721	89,721	71,608	14,547	13,856

Notes: Robustness checks for the intention-to-treat (ITT) regression results shown in column 7 in Table 3 in the main text (paternal survival). The sample in models 1–4 and models 8–12 includes survey respondents who were citizens born in Botswana, at least 18 years old at the time of the census, and born in or after 1975. Binary outcomes were multiplied by 100 to facilitate the interpretation of coefficients and standard errors on a % point scale. Robust standard errors in parentheses. *** p<0.01, ** p<0.05, * p<0.1. Source: Botswana Census 2001 and 2011. YOB=year of birth.

For peer review only - http://bmjopen.bmj.com/site/about/guidelines.xhtml

Table S6. OLS and ITT results: stratified by parental age group

Dependent variable	**Parental disability (1=yes, 0=no)**		
Parental age group	**Age 50-59**	**Age 60-69**	**Age 70-79**
	Risk difference (95% CI)		
A: OLS model			
Schooling (years)	-0.0	-0.3***	-0.1
	(-0.2, 0.1)	(-0.5, -0.1)	(-0.6, 0.3)
B: OLS model			
≥ 10 years of schooling (1=yes, 0=no)	-0.5	-1.6**	-2.0
	(-1.3, 0.3)	(-3.0, -0.2)	(-4.9, 0.9)
C: ITT model			
Reform indicator	-0.6	-1.0	-2.1
	(-2.4, 1.3)	(-4.6, 2.5)	(-10.2, 6.1)
Probability dependent variable, pre-reform	5.3	8.2	13.9
Observations	12,447	6,329	2,262

Notes: Panels A and B show regression results from an OLS model controlling for single-year age indicators, a continuous trend in year of birth, district of birth, and the interaction of each covariate with children's sex. Our models are robust to period effects, which we controlled for implicitly by simultaneously adjusting for children's age and year of birth. Panel C shows regression results from an ITT model (OLS), in which exposure to the reform was defined as a binary indicator (1=year of birth > 1980; 0=otherwise). The sample includes citizens born in Botswana, at least 18 years old at the time of the census, and born in or after 1975. Binary outcomes were multiplied by 100 to facilitate the interpretation of coefficients and standard errors on a % point scale. 95% robust confidence intervals in parentheses. *** p<0.01, ** p<0.05, * p<0.1. Source: Botswana Census 2001 and 2011.

For peer review only - http://bmjopen.bmj.com/site/about/guidelines.xhtml

Table S7. OLS and ITT results: logistic regression models.

Dependent variable	**Mother alive (1=yes, 0=no)**			**Father alive (1=yes, 0=no)**		
Subsample	**Daughters**	**Sons**	**Both sexes**	**Daughters**	**Sons**	**Both sexes**
	Odds ratios (95% CI)					
A: OLS model						
Schooling (years)	1.063***	1.048***	1.055***	1.035***	1.023***	1.028***
	(1.056, 1.071)	(1.041, 1.056)	(1.050, 1.060)	(1.029, 1.041)	(1.017, 1.029)	(1.024, 1.033)
B: OLS model						
≥ 10 years of schooling (1=yes, 0=no)	1.402***	1.309***	1.356***	1.199***	1.091***	1.145***
	(1.328, 1.479)	(1.238, 1.384)	(1.304, 1.409)	(1.148, 1.252)	(1.044, 1.141)	(1.110, 1.181)
C: ITT model						
Reform indicator	0.988	1.054	1.018	1.094	1.052	1.074
	(0.776, 1.258)	(0.816, 1.362)	(0.854, 1.214)	(0.913, 1.311)	(0.869, 1.274)	(0.942, 1.225)
Observations	47,121	42,600	89,721	47,121	42,600	89,721

Notes: Logistic regression models controlling for single-year children's age indicators, a continuous trend in children's year of birth and year of birth squared, and children's district of birth. Regressions for the subsample with both sexes additionally control for an indicator for children's sex and interactions of each covariate with children's sex. Our models are robust to period effects, which we controlled for implicitly by simultaneously adjusting for children's age and year of birth. Panel C shows regression results from an intention-to-treat (ITT) model in which exposure to the reform was defined as a binary indicator (1=year of birth > 1980; 0=otherwise). The sample includes survey respondents who were citizens born in Botswana, at least 18 years old at the time of the census, and born in or after 1975. Robust 95% confidence intervals in parentheses. *** p<0.01, ** p<0.05, * p<0.1. Source: Botswana Census 2001 and 2011.

For peer review only - http://bmjopen.bmj.com/site/about/guidelines.xhtml

References for supplementary webappendix

1. Bor, J.and J.-W. De Neve (2015). "A Social Vaccine? HIV Infection, Fertility, and the Non-Pecuniary Returns to Secondary Schooling in Botswana." Population Association of America Annual Meeting. San Diego [Available at: https://paa2015.princeton.edu/abstracts/151006].
2. Borkum, E. (2009). "Grade structure, educational attainment and labor market outcomes: Evidence from Botswana" (Job Market Paper). Columbia University.
3. Government of Botswana. (1991). National Development Plan - 7. Gaborone, Botswana: Botswana Government Printer.
4. Government of Botswana. (1994). The revised national policy on education. Gaborone, Botswana: Botswana Government Printer.
5. Government of Botswana. (1997). National Population Policy. Gaborone, Botswana: Botswana Government Printer.
6. De Neve J-W, Fink G, Subramanian SV, Moyo S, & Bor J (2015) Length of secondary schooling and risk of HIV infection in Botswana: evidence from a natural experiment. *The Lancet Global Health* 3(8):e470-e477.
7. Statistics Botswana (2013). "Education Report Draft from 2009/10 Botswana Core Welfare Indicators Survey." Gaborone, Botswana: Government of Botswana [Available at: http://www.statsbots.org.bw/].
8. World Health Organization (2020). "Global Health Observatory data repository." World Health Organization. Geneva, Switzerland [Available at: https://www.who.int/data/gho].

For peer review only

STROBE Statement—Checklist of items that should be included in reports of ***cross-sectional studies***

	Item No	Recommendation
**Title and abstract**	1	*(a)* Indicate the study's design with a commonly used term in the title or the abstract<br>Title page
		(b) Provide in the abstract an informative and balanced summary of what was done and what was found<br>Page 3
**Introduction**		
Background/rationale	2	Explain the scientific background and rationale for the investigation being reported<br>Pages 5-6
Objectives	3	State specific objectives, including any prespecified hypotheses<br>Pages 5-6
**Methods**		
Study design	4	Present key elements of study design early in the paper<br>Pages 7-14
Setting	5	Describe the setting, locations, and relevant dates, including periods of recruitment, exposure, follow-up, and data collection<br>Pages 7-8; Text S1 in the Supplementary webappendix
Participants	6	*(a)* Give the eligibility criteria, and the sources and methods of selection of participants<br>Pages 7-8
Variables	7	Clearly define all outcomes, exposures, predictors, potential confounders, and effect modifiers. Give diagnostic criteria, if applicable<br>Pages 8-14
Data sources/ measurement	8*	For each variable of interest, give sources of data and details of methods of assessment (measurement). Describe comparability of assessment methods if there is more than one group<br>Pages 7-9
Bias	9	Describe any efforts to address potential sources of bias<br>Pages 12-14, Pages 19-20
Study size	10	Explain how the study size was arrived at<br>See Figure 2 for a study participant flow diagram
Quantitative variables	11	Explain how quantitative variables were handled in the analyses. If applicable, describe which groupings were chosen and why<br>Pages 8-9
Statistical methods	12	*(a)* Describe all statistical methods, including those used to control for confounding<br>Pages 10-14
		(b) Describe any methods used to examine subgroups and interactions<br>Pages 10-14
		(c) Explain how missing data were addressed<br>Page 14
		(d) If applicable, describe analytical methods taking account of sampling strategy
		(*e*) Describe any sensitivity analyses Pages 13-14
**Results**		
Participants	13*	(a) Report numbers of individuals at each stage of study—eg numbers potentially

For peer review only - http://bmjopen.bmj.com/site/about/guidelines.xhtml

		eligible, examined for eligibility, confirmed eligible, included in the study, completing follow-up, and analysed<br>See Figure 2 for a study participant flow diagram
		(b) Give reasons for non-participation at each stage<br>See Figure 2 for a study participant flow diagram
		(c) Consider use of a flow diagram<br>Figure 2
Descriptive data	14*	(a) Give characteristics of study participants (eg demographic, clinical, social) and information on exposures and potential confounders<br>Page 15
		(b) Indicate number of participants with missing data for each variable of interest
Outcome data	15*	Report numbers of outcome events or summary measures<br>Page 15
Main results	16	*(a)* Give unadjusted estimates and, if applicable, confounder-adjusted estimates and their precision (eg, 95% confidence interval). Make clear which confounders were adjusted for and why they were included<br>Tables 2 and 3
		(b) Report category boundaries when continuous variables were categorized<br>Tables 1 and 2
		(*c*) If relevant, consider translating estimates of relative risk into absolute risk for a meaningful time period Tables 2 and 3 report absolute Risk Differences (95% CIs).
Other analyses	17	Report other analyses done—eg analyses of subgroups and interactions, and sensitivity analyses Pages 17-18
**Discussion**		
Key results	18	Summarise key results with reference to study objectives<br>Page 18
Limitations	19	Discuss limitations of the study, taking into account sources of potential bias or imprecision. Discuss both direction and magnitude of any potential bias<br>Pages 19-20
Interpretation	20	Give a cautious overall interpretation of results considering objectives, limitations, multiplicity of analyses, results from similar studies, and other relevant evidence<br>Pages 18-21
Generalisability	21	Discuss the generalisability (external validity) of the study results<br>Pages 19-20
**Other information**		
Funding	22	Give the source of funding and the role of the funders for the present study and, if applicable, for the original study on which the present article is based<br>Title page

*Give information separately for exposed and unexposed groups.

Note: An Explanation and Elaboration article discusses each checklist item and gives methodological background and published examples of transparent reporting. The STROBE checklist is best used in conjunction with this article (freely available on the Web sites of PLoS Medicine at http://www.plosmedicine.org/, Annals of Internal Medicine at http://www.annals.org/, and Epidemiology at http://www.epidem.com/). Information on the STROBE Initiative is available at www.strobe-statement.org.

For peer review only - http://bmjopen.bmj.com/site/about/guidelines.xhtml
For peer review only

BMJ Open

Causal Effect of Children’s Secondary Education on Parental Health Outcomes – Findings from a Natural Experiment in Botswana

Journal:	*BMJ Open*
Manuscript ID	bmjopen-2020-043247.R1
Article Type:	Original research
Date Submitted by the Author:	31-Oct-2020
Complete List of Authors:	Ludwig, Jan Ole; Heidelberg Univ, Institute of Global Health, Medical Faculty and University Hospital<br>Davies, Neil; University of Bristol, Medical Research Council Integrative Epidemiology Unit; Bristol Medical School, Population Health Sciences<br>Bor, Jacob; Boston University, Global Health; Boston University School of Public Health, Department of Epidemiology<br>De Neve, JW; Heidelberg Univ, Institute of Global Health, Medical Faculty and University Hospital
**Primary Subject Heading**:	Epidemiology
Secondary Subject Heading:	Health economics, Public health
Keywords:	EPIDEMIOLOGY, HEALTH ECONOMICS, PUBLIC HEALTH

SCHOLARONE™ Manuscripts

I, the Submitting Author has the right to grant and does grant on behalf of all authors of the Work (as defined in the below author licence), an exclusive licence and/or a non-exclusive licence for contributions from authors who are: i) UK Crown employees; ii) where BMJ has agreed a CC-BY licence shall apply, and/or iii) in accordance with the terms applicable for US Federal Government officers or employees acting as part of their official duties; on a worldwide, perpetual, irrevocable, royalty-free basis to BMJ Publishing Group Ltd ("BMJ") its licensees and where the relevant Journal is co-owned by BMJ to the co-owners of the Journal, to publish the Work in this journal and any other BMJ products and to exploit all rights, as set out in our licence.

The Submitting Author accepts and understands that any supply made under these terms is made by BMJ to the Submitting Author unless you are acting as an employee on behalf of your employer or a postgraduate student of an affiliated institution which is paying any applicable article publishing charge ("APC") for Open Access articles. Where the Submitting Author wishes to make the Work available on an Open Access basis (and intends to pay the relevant APC), the terms of reuse of such Open Access shall be governed by a Creative Commons licence – details of these licences and which Creative Commons licence will apply to this Work are set out in our licence referred to above.

Other than as permitted in any relevant BMJ Author's Self Archiving Policies, I confirm this Work has not been accepted for publication elsewhere, is not being considered for publication elsewhere and does not duplicate material already published. I confirm all authors consent to publication of this Work and authorise the granting of this licence.

For peer review only

Causal Effect of Children's Secondary Education on Parental Health Outcomes – Findings from a Natural Experiment in Botswana

Authors: Jan Ole Ludwig, B.M.[1], Neil M. Davies, Ph.D.[2,3,4], Jacob Bor Sc.D. S.M.[5,6], Jan-Walter De Neve, M.D. Sc.D.[1,*]

Affiliations:

[1] Heidelberg Institute of Global Health, Medical Faculty and University Hospital, Heidelberg University, Im Neuenheimer Feld 130.3, Heidelberg 69120, Germany.

[2] Medical Research Council Integrative Epidemiology Unit, University of Bristol, BS8 2BN, United Kingdom.

[3] Population Health Sciences, Bristol Medical School, University of Bristol, Barley House, Oakfield Grove, Bristol, BS8 2BN, United Kingdom.

[4] K.G. Jebsen Center for Genetic Epidemiology, Department of Public Health and Nursing, NTNU, Norwegian University of Science and Technology, Norway.

[5] Department of Global Health, Boston University School of Public Health, Boston, MA 02118, United States.

[6] Department of Epidemiology, Boston University School of Public Health, Boston, MA 02118, United States.

Contributions Statement:

JOL and JWDN conceived and designed the study. JL conducted the statistical analyses under the guidance of NMD, JB, and JWDN. JOL wrote the first draft of the report. NMD, JB, and JWDN reviewed and contributed important revisions to the report. All authors approved the final submitted version of the report.

Conflict of Interest:

The authors declare that they have no conflict of interest.

Funding:

JOL was supported by the Else Kröner-Fresenius-Stiftung within the Heidelberg Graduate School of Global Health (grant number: not applicable). NMD was supported by an Economics and Social Research Council (ESRC) Future Research Leaders grant (grant number: ES/N000757/1) and a

For peer review only - http://bmjopen.bmj.com/site/about/guidelines.xhtml

Norwegian Research Council grant (grant number: 295989). JB and JWDN were supported by the NICHD of National Institutes of Health (grant number: R03-HD098982). JWDN was also supported by the Alexander von Humboldt Foundation, funded by Germany's Federal Ministry of Education and Research Health (grant number: not applicable), German Research Foundation (grant number: 405898232), and Heidelberg University's Excellence Initiative Health (grant number: not applicable). The funders had no role in study design, data collection and analysis, decision to publish, or preparation of the manuscript. The contents are the responsibility of the authors and do not necessarily reflect the views of any of the funders or the US government.

Data Sharing Statement:

Data is publicly available at no cost from IPUMS (http://ipums.org).

Acknowledgments:

We thank respondents in the Botswana 2001 and 2011 Census, and staff at the Botswana Central Statistics Office. We also thank Prashant Bharadwaj for helpful comments.

***Correspondence:**

Jan-Walter De Neve, Heidelberg Institute of Global Health, Medical Faculty and University Hospital, Heidelberg University, Im Neuenheimer Feld 130.3, Heidelberg 69120, Germany. E-mail: janwalter.deneve@uni-heidelberg.de. Phone: +49 (0) 6221 56-32873.

For peer review only

Abstract

Objectives: A growing literature highlights the intergenerational transmission of human capital, from parents to children. However, far less is known about "upward transmission", from children to parents. In this study we use a 1996 Botswana education policy reform as a natural experiment to identify the causal effect of children's secondary schooling on their parents' health.

Setting: Botswana's decennial census (2001 and 2011). Data were obtained through the Integrated Public Use Microdata Series (IPUMS) and are 10% random samples of the complete population in each of these census years (i.e., 'full censuses').

Participants: Survey respondents who were citizens born in Botswana, at least 18 years old at the time of the census, and born in or after 1975 ($N = 89{,}721$).

Primary and secondary outcome measures: Parental survival and disability at the time of the census, separately for mothers and fathers.

Results: The 1996 reform caused a large increase in grade ten enrollment, inducing an additional 0.4 years of schooling for the first cohorts affected (95% Confidence Interval: 0.3 – 0.5, $P < 0.001$). The reform, however, had no effect on parental survival and disability by the time exposed child cohorts reach age 30. Results were robust to a wide array of sensitivity analyses.

Conclusions: This study found little evidence that parents' survival and disability was affected by their offspring's educational attainment in Botswana. Parents' health may not be necessarily affected by increasing their offspring's educational attainment.

Keywords: Children's Education; Parental Health; Intergenerational; Natural Experiment; Instrumental Variable Analysis; Low- and Middle-Income Countries

For peer review only - http://bmjopen.bmj.com/site/about/guidelines.xhtml

Strengths and Limitations of this Study

- While a large literature has investigated the role of parental human capital on children's well-being, relatively little is known about "upward transmission", from children to parents.
- We explore the 1996 Botswana education policy reform as a natural experiment to estimate the causal effect of children's secondary education on their parents' health.
- Using nearly 90,000 census records from 2001 and 2011, we show that the 1996 reform resulted in an additional 0.4 years of schooling among exposed child cohorts.
- Children's education, however, had few detectable effects on parental survival and disability. Parents' health may not be affected by increasing their offspring's education.
- Limitations of our study include limited generalizability beyond the reform analyzed and lack of information on pathways and alternative parental health outcomes.

For peer review only

1. Introduction

Investments in children's human capital may yield large dividends not only for themselves (1) and their offspring (2, 3), but also for their parents. While a large literature has documented the substantial resource flows from parents to children, a growing literature from Asia (4), Latin America (5), and sub-Saharan Africa (6) suggests that intergenerational wealth transfers have started to reverse in aging low- and middle-income societies where governmental support for older adults is limited (7). In addition to sharing their wealth with parents, better-educated children may share their increased knowledge (8) and skills (9) with older household members, and may be better equipped to interact with the health system to help manage the health needs of their aging parents. Additional education may also open access to careers in the health sector, leading to increased intra-family medical expertise, with positive health benefits for family members (10). Adult children have been suggested to "repay" their parents for investments received in childhood, particularly in settings where families function as de facto social institutions (11).

The past decades have brought a massive increase in educational opportunities for younger cohorts in low- and middle-income countries (LMICs). Gross domestic product per capita in LMICs more than doubled between 2000 and 2018, from 2,310 to 4,662 in 2010 USD (12). The extent to which this younger generation shares its increased resources with their parents, however, remains largely unknown. More than a dozen studies have reported a positive correlation between children's human capital and parental health, including in China (13), India (14), and South Africa (15). Given the large number of potential factors (such as genetic traits) that may confound the relationship between children's human capital and parental health, however, these correlations are difficult to interpret and are unlikely to identify causal effects of educational opportunities

For peer review only - http://bmjopen.bmj.com/site/about/guidelines.xhtml

provided to children. A recent review concluded that spillover effects from offspring to parents are understudied and (quasi-)experimental studies from low-resource settings are needed (16).

In this study, we explore a large secondary schooling reform in sub-Saharan Africa to determine the impact of increasing educational investment in younger generations on their parents. In 1996, the government of Botswana launched a major education reform. Between 1997 and 1998, grade ten enrollment in the country more than doubled, from 15,437 to 36,562 (**Figure** 1, see **Text S1** and **Table S1)** in the supplementary materials for additional background). At the cohort level, the reform induced an additional 0.4 years of schooling among affected cohorts relative to the attainment the affected cohorts would have achieved if previous trends had continued. This reform constitutes a 'natural experiment' through comparison of cohorts exposed to the reform versus those unexposed, while controlling for gradual changes across cohorts. Using two waves of Botswana's decennial census (2001, 2011) to disentangle age and cohort effects, we used the resulting variation in exposure to identify the causal effect of children's secondary education on parental health.

(FIGURE 1 AROUND HERE)

For peer review only - http://bmjopen.bmj.com/site/about/guidelines.xhtml

2. Material and Methods

2.1. Data Sources and Study Population

Data for this study were obtained from the Botswana Population and Housing Census 2001 and 2011 through the Integrated Public Use Microdata Series (IPUMS) (17). The microdata provided by IPUMS are 10% random samples of the complete population in each of these years (i.e., 'full censuses'). Subnational administrative boundaries were harmonized across censuses by IPUMS. The censuses were conducted by the Central Statistics Office Botswana and included all persons in the country at the time of the census without regard of their usual residence. The censuses also collected basic demographic information on members of the household who were citizens outside of the country on the census night, but who would typically live in the household if they would have been in Botswana. In the 2011 Census, for instance, 24,561 citizens were living outside of Botswana (1.2%). Data on sex, age, years of schooling, and the survival status of mothers and fathers was available for 88.9% of respondents, yielding a total of 333,334 individuals in the pooled 2001 and 2011 census dataset (**Figure 2**). The census data do not allow matching individuals between census years.

(FIGURE 2 ABOUT HERE)

The study population included all citizens of Botswana and born in Botswana. Children ages younger than 18 were excluded because they would not have had the opportunity to complete secondary school. Children born prior to 1975 were excluded because previous secondary school

For peer review only - http://bmjopen.bmj.com/site/about/guidelines.xhtml

reforms led to changes in education for these older cohorts. Using a narrow subsample of pre- and post-reform cohorts (i.e., born between 1975 and 1993 based on the age and birth cohort cutoffs described above), there were good reasons to believe that control cohorts differed from exposed cohorts mostly with respect to their access to grade ten. We explore alternative sample specifications in sensitivity analyses (**Section 2.6**). Children not born in Botswana were excluded because they would not have been exposed to the reform if they migrated in adulthood.

2.2. Measurement of Exposure and Endpoints

Data on education, age in years, gender, citizenship, district of birth, parental survival, parental disability, parental age, and parental years of schooling were extracted from the census datasets. Year of birth was calculated based on age collected in the census. The key independent variable of interest was the child's total years of schooling ("What is the highest grade/level that [respondent] has completed?"). Only formal schooling was counted. Years of school accounts for the number of years of study regardless of the track or kind of study. Our main outcome of interest was parental survival status, separately for mothers and fathers. Parental survival status indicated whether the person's biological parent was alive at the time of the census (e.g., "Is [the person's] biological mother alive?") and was observed regardless of whether the parent is co-resident with the adult child. During the period of study, Botswana had very high adult mortality rates, due in large part to the country's HIV epidemic. In 2001, the probability of a 15-year old reaching age 60 ($_{45}e_{15}$) was about 37% (18). In this context, children's education could affect parental survival because information about HIV was disseminated via secondary school "life skills" curricula (19); because HIV stigma may be lower among the better educated, leading to higher HIV testing (20); and/or because better educated children may help their parents navigate the health system and gain access to HIV treatment (9). Other major determinants of morbidity and mortality among adults

For peer review only - http://bmjopen.bmj.com/site/about/guidelines.xhtml

ages 50 – 69 years in Botswana include cardiovascular diseases, neoplasms, and diabetes and kidney diseases (21). Diabetic retinopathy and peripheral neuropathy, for instance, are increasingly common causes of severe visual impairment and non-traumatic lower extremity disability, including in the context of sub-Saharan Africa.

Pointer variables provided by IPUMS indicate the location within the household of every child's parent, and make it possible to construct individual-level variables representing the characteristics of co-resident parents. For the subset of households with surviving, co-resident parents and children, we were therefore able to assess the impact of the schooling reform on key parental socio-economic characteristics and disability, as additional secondary outcomes. Children's education, for instance, has been suggested to affect parental fertility decisions by creating additional costs (educational expenses) and decreasing the perceived benefits derived from children by removing them from the family productive system (22). The census also asked a series of questions about disability in the 2001 and 2011 waves. Specifically, they asked about employment disability, blind- or vision-impaired, deaf or hearing-impaired, mute or speech-impaired, disability affecting lower extremities, disability affecting upper extremities, mental disability, and psychological disability. For our analysis, we coded the following parental disability variables: "employment disability" defined as whether a parent was economically inactive because of health-related reasons; "blind or vision-impaired" defined as whether a parent was blind or had limited vision; and "disability affecting lower extremities" defined as whether a parent was unable to use one or both legs (17). Disabilities were reported by a household respondent (typically the household head). Data on financial transfers between children and their parents, frequency of contact between children and parents, and the uptake of social or healthcare services were not collected during the census.

For peer review only - http://bmjopen.bmj.com/site/about/guidelines.xhtml

2.3. Unit of Analysis

Our unit of analysis was the adult child. In doing so, we follow the data structure and variables provided by IPUMS closely, which maps well unto our analytical approach since our identification strategy is based on cohort-specific variation to the education reform at the child level. Moreover, the census datasets do not contain information on all offspring's educational attainment which precludes an analysis at the parent level (17). The census datasets thus differ from more standard surveys focused on older adults and which contain information on the educational attainment of all offspring (e.g., the Health and Retirement Study in the United States). For our main analysis, we included adult children regardless of whether they were the head of household, their marital status, or whether the adult child co-resided with their parent(s).

2.4. Instrumental Variable Analysis

An instrumental variable (IV) is a variable that is associated with the treatment of interest, but is independent of confounding factors and has no direct effect on the outcome (23). In our application, the instrument was a 'reform cohort' indicator, taking the value one if the respondent was born in a cohort that was exposed to the education policy reform and zero otherwise. In Botswana, children were historically expected to start primary school at age seven years and enter junior secondary school at age 15 years. Individuals born in 1981 or later would therefore have entered junior secondary school in 1996 or later and were classified as exposed (**Figure S1**). We used this exogenous variation in educational attainment resulting from the policy reform to identify the causal effect of children's education on their parents' survival. First, we assessed whether children's cohorts exposed to the reform had higher educational attainment than birth cohorts not exposed to the reform using multivariable adjusted ordinary least squares (OLS) linear

For peer review only - http://bmjopen.bmj.com/site/about/guidelines.xhtml

probability regression models. We estimated the effect of exposure to the reform on total years of schooling completed and the probability of completing at least ten years of schooling. Second, as a benchmark for our causal analysis, we estimated the 'naïve' cross-sectional association of children's education with their parents' survival, acknowledging that this association may reflect reverse causality and effects of confounders and therefore does not have a causal interpretation. We assessed the crude relationship graphically and in multivariable adjusted regression analyses. Third, we assessed the intention-to-treat (ITT) effect of having a child affected by the reform on parental survival. We examined the relationship graphically and adjusted for covariates. Fourth, we estimated 2SLS (instrumental variables) regression models, using exposure to the reform as an instrument for total years of children's schooling while adjusting for covariates. Natural experiments that change the probability of an exposure can be analyzed like randomized controlled trials with non-compliance. Exposure to the reform was modeled as an intercept shift for cohorts affected by the reform. Specifically, we estimated first stage, ITT, and 2SLS models of the form:

(1) $Children'sEduc_i=\beta_0+\beta_1 1[Children'sYearofBirth>1980]_i+\beta_2 f(Children'sYearofBirth)+\beta_3 1[Children'sAge]i+\beta_4 1[Children'sDistrictofBirth]_i+\varepsilon_i$

(2) $ParentalSurvival_i=\gamma_0+\gamma_1 1[Children'sYearofBirth>1980]_i+\gamma_2 f(Children'sYearofBirth)+\gamma_3 1[Children'sAge]i+\gamma_4 1[Children'sDistrictofBirth]_i+v_i$

(3) $ParentalSurvival_i=\alpha_0+\alpha_1 Children'sEduc_i\_hat+\alpha_2 f(Children'sYearofBirth)+\alpha_3 1[Children'sAge]_i+\alpha_4 1[Children'sDistrictofBirth]_i+\zeta_i$

with $Children'sEduc_i\_hat$ *predicted in (1).*

In all models, we controlled for children's single-year age indicators ($Children'sAge_i$) to account for trends in age and education across children's cohorts. We focused on this age specification as

For peer review only - http://bmjopen.bmj.com/site/about/guidelines.xhtml

it is highly flexible, but assess alternate specifications in sensitivity analyses. We also included a linear term in children's year of birth and indicators for children's district of birth. We estimated models for women and men separately, as well as a pooled sample. When pooling sexes, we additionally included children's sex and the interactions of sex with all other covariates. Data on parent's own socio-demographic characteristics was also available for the sub-sample of children co-residing with a least one parent. When testing the association between children's education and secondary parental outcomes in a 'conventional' multivariable regression analysis, we therefore additionally included controls for parental age (years) and parental education (years of schooling).

2.5. Assumptions for Causal Inference

For a causal interpretation of our effect estimates, four assumptions must be satisfied (see **Figure S2** for a directed acyclic graph). First, our instrument must be associated with educational attainment. This is testable and we found evidence of large effects (F-statistic: 65.6). The large value of the F-statistic indicates that our instrument was strongly associated with schooling. Second, the instrument must be independent of unobserved confounders of the instrument-outcome association conditional on observed covariates. This implies that children born before and after the reform cutoff were similar, after controlling for children's single-year age indicators, a continuous term in children's year of birth, and children's district of birth. Critically, the availability of multiple census years allowed us to generate variation in children's age in a given birth cohort group. Our models are therefore also robust to the existence of period effects, which we control for by simultaneously adjusting for children's age and a continuous term in year of birth (noting that census_year = birth cohort + age). We also assessed alternative specifications of children's age (quadratic, cubic, quartic) and year of birth (quadratic), and restricted the sample

to a narrower birth cohort window around the reform cutoff (+/- 3 years and +/- 5 years around the cutoff). We also allowed for different linear slopes in year of birth on either side of the reform cutoff akin to a 'fuzzy' regression discontinuity design (24).

We also used covariate balance plots to estimate the association of our exposure (schooling) and our instrument (reform indicator) with observed covariates, as described in detail elsewhere (25, 26). While this analysis cannot prove that our instrument is not associated with unobserved covariates, it can provide some indication if our instrument is less strongly associated with measured covariates than our exposure. Third, for our 2SLS estimates to have a causal interpretation, we assume that exposure to the policy reform affected our parental outcomes only through changes in secondary schooling. This assumption is plausible given that the education reform was a supply-side intervention (19), which would not have affected parents except through their children's increased access to school. Fourth, to interpret our results as local average treatment effects, we assume that there are no defiers (i.e., the monotonicity assumption) (27).

2.6. Sensitivity Analyses

In addition to the robustness checks described above, we conducted a wide range of sensitivity analyzes, including earlier birth cohorts (i.e., those born between 1971 and 1993, instead of those born between 1975 and 1993), modelling the outcome using a logit link function, and controlling for an indicator for heap year (defined as ages that are a multiple of five) to account for measurement error in age due to potential age heaping. Results could also be biased by differential response rates by birth cohort, manipulation of our 'running variable', or by selection bias from mortality risk or migration associated with being born after 1980. We therefore assessed cohort

For peer review only - http://bmjopen.bmj.com/site/about/guidelines.xhtml

sizes by birth cohort (**Figure S3**). We note that we present our results for secondary parental outcomes under the null hypothesis that there is no effect of the reform on parental survival.

To further probe the robustness of our main findings, we conducted a number of subsample analyses. Specifically, we included households for whom additional education among children was most likely to have relatively large consequences for parental survival. Our hypothesis was that the role of children's resources may be more pronounced in households where: i) children were more likely to be affected by the reform, ii) there was a large intergenerational gap in educational attainment between adult children and their parents, and iii) parents were on average older and thus more likely to be 'dependent' on the support of children. To do so, we limited the sample to children with at least nine years of schooling (i.e., those primarily affected by the policy reform). For the subset of households with co-resident parents and children, we also limited the sample to households where the difference in educational attainment between parents and children was $\geq$ 5.5 years (50th percentile), and stratified our model by parental ten-year age groups.

2.7. Data, Code Availability, and Ethics

This was a complete case analysis and all analyses were conducted in StataMP 15.1. Data is publicly available at no cost from IPUMS (http://ipums.org) (17). Code used to generate these results can be found at https://github.com/jwdeneve/Botswana-ParentalHealth. This study was pre-registered and approved by the Heidelberg University Hospital Ethics Committee [S-186/2018].

2.8. Patient and Public Involvement

Patients or the public were not involved in the design, or conduct, or reporting, or dissemination plans of our research.

For peer review only - http://bmjopen.bmj.com/site/about/guidelines.xhtml

3. Results

3.1. Descriptive Statistics

On average the children in our analytical sample were 24.8 years old (**Table 1**). Across 34,202 children born pre-reform, 13,073 (38.2%) had lost their father and 5,666 (16.6%) had lost their mother. In terms of household characteristics, the average number of family members per household was 5.0. In our subset of households with surviving, co-resident parents and children, there were a total of 29,266 (32.6%) child-parent dyads (10,082 of which were living with both parents). Among those, the average parental age was 50.8 and 57.0 years for mothers and fathers, respectively. The average gap in attained education between children and co-residing parents was substantial (5.2 and 6.0 years of schooling for mothers and fathers, respectively). **Table S2** shows detailed descriptive statistics for the subset of children co-residing with at least one parent.

(TABLE 1 AROUND HERE)

3.2. Association Between Children's Education and Parental Survival

Figure 3 illustrates heterogeneity in the unadjusted relationship between children's education and parental survival, separately for maternal and paternal survival. The positive association between children's education and parental survival was non-linear, levelling off after junior secondary school. In 'conventional' multivariable adjusted regression analysis, children's schooling was associated with increased parental survival. In the pooled (daughters and sons) sample, each additional year of children's schooling was associated with a 0.8 percentage point increase (95%

For peer review only - http://bmjopen.bmj.com/site/about/guidelines.xhtml

Confidence Interval [CI]: 0.7 – 0.9) and a 0.6 percentage point increase (95% CI: 0.5 – 0.7) in the probability of maternal and paternal survival, respectively. These associations persisted after controlling for parent's own socio-demographic characteristics in the subset of households with co-resident parents and children (**Table S3**). Although suggestive, these associations may be vulnerable to reverse causality or confounding by unobserved characteristics.

(FIGURE 3 AROUND HERE)

3.3. Causal Effect of Children's Education on Parental Survival

Table 2 shows the estimated reform impact on children's educational attainment, as indicated by total years of schooling completed and the probability of having completed at least ten years of schooling. All models controlled flexibly for single-year age indicators, a continuous term in year of birth, indicators for district of birth, and census year. In the pooled sample, the estimated effect of the reform on educational attainment was 0.4 additional years of schooling (95% CI: 0.3 – 0.5) among exposed child cohorts. Exposure to the reform was also associated with a 19.0 percentage point increase (95% CI: 17.5 – 20.4) in the probability of having completed at least ten years of schooling relative to a baseline probability of 43.5% among pre-reform child cohorts.

(TABLE 2 AROUND HERE)

For peer review only - http://bmjopen.bmj.com/site/about/guidelines.xhtml

Figure S4 in the supplementary webappendix plots parental survival and disability against reform exposure. We found little evidence of a discontinuity for post-reform cohorts. Multivariable regression models – which adjusted for children's single-year age indicators, a continuous trend in children's year of birth, district of birth indicators, and census year indicators –, presented in **Table 3**, also provide little evidence (at alpha=0.05, with 80% power) that exposure to the reform affected maternal survival (-0.2 percentage points; 95% CI: -1.3 – 0.9) or paternal survival (0.5 percentage points; 95% CI: -0.9 – 2.0). Our results were generally not sensitive to different modelling strategies for age, different specifications of the outcome, sample inclusion criteria (**Tables S3** – **S6**) or model (**Table S7**). The differences in potential confounders for the actual exposure (schooling) and the proposed instrument (reform cohort) are presented in **Figure S5**. These results suggest that the instrumental variable analysis would be generally less biased from these measured potential confounders than the multivariable adjusted regression analysis. One variable appeared to differ for the instrument (maternal schooling); however, adjusting for this variable did not meaningfully affect our results.

(TABLE 3 AROUND HERE)

For peer review only - http://bmjopen.bmj.com/site/about/guidelines.xhtml

3.4. Children's Education and Secondary Parental Outcomes

In **Table 4**, we show results for our secondary parental outcomes, including key parental demographic and socio-economic characteristics and disability categories. Consistent with our main results for maternal and paternal survival, we found little evidence that children's exposure to the reform affected any of these secondary parental outcomes (*Panel C*, **Table 4**).

(TABLE 4 AROUND HERE)

4. Discussion

Using census data from 89,721 adult children, we find that additional secondary education in children had few detectable survival benefits for their parents in Botswana. Although children's education was strongly associated with improved parental health in OLS regression models, these results could be due to e.g., reverse causality whereby chronically ill parents require care from children before completion of school. Our ITT estimates, in contrast, suggest that it is unlikely that children's exposure to the reform increases maternal and paternal survival by more than 0.9 and 2.0 percentage points, respectively, which corresponds to the value of the upper limit of the 95% confidence interval. Our 2SLS estimates suggest that is unlikely that each additional year of schooling increases maternal and paternal survival by more than 2.3 and 4.9 percentage points, respectively. Effects were consistent across a wide array of robustness checks, including alternative specifications of long-run trends, analytical sample, and multiple parental outcomes.

For peer review only - http://bmjopen.bmj.com/site/about/guidelines.xhtml

As with all instruments, a number of assumptions underpin a causal interpretation of these findings. A key assumption underlying our model is that the timing of children's birth around the reform period is random conditional on covariates (no confounding assumption). We controlled for generic time trends in our multivariable models to absorb gradual changes in child and parental characteristics over time. The main logic underlying our identification strategy is that the reform led to an unanticipated *additional* increase in children's schooling among affected cohorts.

There are many reasons why parental socio-economic characteristics and health outcomes might change across children's birth cohorts. Notably, in the early 2000s, Botswana became the first country in sub-Saharan Africa to offer large-scale public treatment for HIV to all qualifying citizens, which had massive and immediate effects on economic and health outcomes (28). Botswana has also implemented some of the most successful drought relief programs in Southern Africa, which were implemented to address the loss of livestock and improve nutritional outcomes (29). These changes in public health prevention programming and socio-economic context in Botswana, however, tend to be gradual over time and/or affect children of different ages. These phenomena would result in gradual changes in parental survival across children's ages and would be picked up in our controls for underlying trends in children's age. The availability of multiple census years also allowed us to generate variation in children's age in a given birth cohort. Our models were therefore robust to period effects (e.g., differences in HIV mortality and health systems across census years), which we controlled for implicitly by simultaneously adjusting for children's age and year of birth. Although violations of these instrumental variable conditions appear unlikely in our study, it is impossible to prove that they are definitively met.

For peer review only - http://bmjopen.bmj.com/site/about/guidelines.xhtml

While the educational reform likely allows us to identify the causal effects of interest, relying on changes generated by the 1996 education reform and census data comes with limitations in terms of external validity. First, all of the variation explored reflects variation in secondary schooling. Effects of schooling may be qualitatively and quantitatively different in primary school (30, 31) or higher education (10), and indeed the association between measured schooling and parental survival was non-linear. Second, the causal effects we identify do not necessarily represent the causal effects in the general population but in the subpopulation of compliers (i.e. children who attained increased schooling because of the reform). Nevertheless, one advantage of using Botswana's supply-side school reform is the important size of its impact (nearly half a year of additional schooling on a population level), and that it affected a large segment of the population. Third, educational attainment reported in the census may be subject to measurement error, which may bias the multivariable adjusted estimates towards the null. The instrumental variable analysis, however, is robust to classical (balanced) measurement error in education. Fourth, the opportunity costs of schooling (e.g., not being able to work) are part and parcel of schooling, and it is impossible to increase schooling without reducing whatever activities children would have been doing at the time. Therefore, the exposure could be most precisely stated as an additional year of schooling – and a year less of whatever the child would have been doing otherwise. Fifth, our measures of parental health may be susceptible to reporting bias. Self-reported health, however, has been suggested to be a valid and reliable indicator when assessing the health of adults in low- and middle-income settings (32). Sixth, parental disability outcomes which are available in the census data, such as visual impairment, are severe health outcomes. Children's education may affect other parental health outcomes or health behaviors, such as HIV treatment, smoking, or salt consumption. In China, for instance, an education program delivered to school children as part of

the formal school curriculum reduced cardiovascular risk factors among their parents (33). In Malawi, a large school-based intervention is currently under way to reduce cardiovascular risk factors in children and their family members (34). Non-communicable diseases, in particular, are expected to pose an increasing challenge in Southern Africa, where health systems have focused on tackling infectious diseases and maternal and child health. Seventh, we observed exposed children only up to age 30 years. Parents' survival and disability may not have been affected during this time period. However, this is a common limitation of prevention studies and our analysis captures longer follow-up than randomized controlled trials which observe parental health over a shorter (e.g., 1-2 year) horizon (33). Eighth, the census data only provided information on our secondary parental outcomes for the subset of children living with at least one parent. The effect of children's education on parental disability, for instance, may differ for children and parents who are not living under the same roof. Additionally, selection bias may occur if the decision of children to co-reside with parents depends on exposure to the reform and/or our outcomes for parental disability. However, our estimates for parental disability would only be biased by selection if the reform affected parental disability (i.e., our estimates are likely to be a valid test of the null hypothesis that offspring education does not affect their parents' health, even if there is selection bias) (35). Further investigation is warranted to examine if – and how – involving children in their parents' care may improve the health of the older generation in the region.

Overall, our findings suggest that increasing secondary education of offspring may play a more muted role in the survival and disability of parents than suggested by non-experimental observational and longitudinal studies in LMICs. The lack of parental health gains weakens the evidence base for large "upward" intergenerational benefits of human capital investment.

Contributions Statement:

JOL and JWDN conceived and designed the study. JL conducted the statistical analyses under the guidance of NMD, JB, and JWDN. JOL wrote the first draft of the report. NMD, JB, and JWDN reviewed and contributed important revisions to the report. All authors approved the final submitted version of the report.

Conflict of Interest:

The authors declare that they have no conflict of interest.

Funding:

JOL was supported by the Else Kröner-Fresenius-Stiftung within the Heidelberg Graduate School of Global Health (grant number: not applicable). NMD was supported by an Economics and Social Research Council (ESRC) Future Research Leaders grant (grant number: ES/N000757/1) and a Norwegian Research Council grant (grant number: 295989). JB and JWDN were supported by the NICHD of National Institutes of Health (grant number: R03-HD098982). JWDN was also supported by the Alexander von Humboldt Foundation, funded by Germany's Federal Ministry of Education and Research Health (grant number: not applicable), German Research Foundation (grant number: 405898232), and Heidelberg University's Excellence Initiative Health (grant number: not applicable). The funders had no role in study design, data collection and analysis, decision to publish, or preparation of the manuscript. The contents are the responsibility of the authors and do not necessarily reflect the views of any of the funders or the US government.

Data Sharing Statement:

Data is publicly available at no cost from IPUMS (http://ipums.org).

Acknowledgments:

We thank respondents in the Botswana 2001 and 2011 Census, and staff at the Botswana Central Statistics Office. We also thank Prashant Bharadwaj for helpful comments.

For peer review only

References

1. Cutler DM, Lleras-Muney A. Education and Health: Evaluating Theories and Evidence. In: J House, R Schoeni, G Kaplan, Pollack H, editors. Making Americans Healthier: Social and Economic Policy as Health Policy. New York: Russell Sage Foundation; 2008.

2. Karlsson O, De Neve J-W, Subramanian SV. Weakening association of parental education: analysis of child health outcomes in 43 low- and middle-income countries. International Journal of Epidemiology. 2019; 48(1):83-97. doi:10.1093/ije/dyy158

3. Mensch BS, Chuang EK, Melnikas AJ, Psaki SR. Evidence for causal links between education and maternal and child health: systematic review. Tropical Medicine & International Health. 2019; 24(5):504-22. doi:10.1111/tmi.13218

4. Cai F, Giles J, Meng X. How well do children insure parents against low retirement income? An analysis using survey data from urban China. Journal of Public Economics. 2006; 90(12):2229-55. doi:10.1016/j.jpubeco.2006.03.004

5. Bravo J. Intergenerational transfers and social protection in Latin America. National Transfers Accounts Working Paper Series2007.

6. Kohler IV, Kohler HP, Anglewicz P, Behrman JR. Intergenerational transfers in the era of HIV/AIDS: Evidence from rural Malawi. Demogr Res. 2012; 27:775-834.

7. Bloom DE, Canning D, Fink G. Implications of population ageing for economic growth. Oxford Review of Economic Policy. 2011; 26(4):583-612. doi:10.1093/oxrep/grq038

8. Kuziemko I. Human Capital Spillovers in Families: Do Parents Learn from or Lean on Their Children? Journal of Labor Economics. 2014; 32(4):755-86. doi:10.1086/677231

9. Evans R, Becker S. Children caring for parents with HIV and AIDS: global issues and policy responses. Bristol, UK ; Portland, OR: Policy Press; 2009.

10. Chen Y, Persson P, Polyakova M. The Roots of Health Inequality and The Value of Intra-Family Expertise. National Bureau of Economic Research (NBER) Working Paper No. 25618. 2019; [Available at: https://www.nber.org/papers/w25618].

11. Frankenberg E, Lillard L, Willis RJ. Patterns of Intergenerational Transfers in Southeast Asia. Journal of Marriage and Family. 2002; 64(3):627-41. doi:10.1111/j.1741-3737.2002.00627.x

12. World Bank. World Bank national accounts data, and OECD National Accounts data files. [Available at: https://data.worldbank.org/indicator]. 2019. https://data.worldbank.org/indicator. Accessed 18 May 2019.

13. Yang L, Martikainen P, Silventoinen K. Effects of Individual, Spousal, and Offspring Socioeconomic Status on Mortality Among Elderly People in China. Journal of Epidemiology. 2016; 26(11):602-9. doi:10.2188/jea.JE20150252

For peer review only - http://bmjopen.bmj.com/site/about/guidelines.xhtml

14. Thoma B, Sudharsanan N, Karlsson O, Joe W, Subramanian SV, De Neve J-W. Children's education and parental old-age health: Evidence from a population-based, nationally representative study in India. Population Studies. 2020:1-16. doi:10.1080/00324728.2020.1775873

15. De Neve J-W, Harling G. Offspring schooling associated with increased parental survival in rural KwaZulu-Natal, South Africa. Social Science & Medicine. 2017; 176:149-57. doi:10.1016/j.socscimed.2017.01.015

16. De Neve J-W, Kawachi I. Spillovers between siblings and from offspring to parents are understudied: A review and future directions for research. Social Science & Medicine. 2017; 183:56-61. doi:10.1016/j.socscimed.2017.04.010

17. Integrated Public Use Microdata Series. Integrated Public Use Microdata Series, International: Version 6.3 [Machine-readable database]. In: University of Minnesota, editor. Minneapolis2014.

18. World Health Organization. Global Health Observatory data repository. In: World Health Organization, editor. Geneva, Switzerland [Available at: https://www.who.int/data/gho]2020.

19. Government of Botswana. The revised national policy on education. Gaborone, Botswana: Botswana Government Printer; 1994.

20. Stangl AL, Lloyd JK, Brady LM, Holland CE, Baral S. A systematic review of interventions to reduce HIV-related stigma and discrimination from 2002 to 2013: how far have we come? Journal of the International AIDS Society. 2013; 16:18734. doi:10.7448/ias.16.3.18734

21. Global Burden of Disease. GBD Results Tool. [Available at: http://ghdx.healthdata.org/gbd-results-tool]. 2020.

22. Axinn WG. The Effects of Children's Schooling on Fertility Limitation. Population Studies. 1993; 47(3):481-93.

23. Hernán MA, Robins JM. Instruments for causal Inference: an epidemiologist's dream? Epidemiology. 2006; 17(4):360-72. doi:10.1097/01.ede.0000222409.00878.37

24. Bor J, Moscoe E, Mutevedzi P, Newell M-L, Bärnighausen T. Regression Discontinuity Designs in Epidemiology. Epidemiology. 2014; 25(5):729-37. doi:10.1097/ede.0000000000000138

25. Davies NM, Thomas KH, Taylor AE, et al. How to compare instrumental variable and conventional regression analyses using negative controls and bias plots. International Journal of Epidemiology. 2017; 46(6):2067-77. doi:10.1093/ije/dyx014

26. Jackson JW, Swanson SA. Toward a Clearer Portrayal of Confounding Bias in Instrumental Variable Applications. Epidemiology. 2015; 26(4):498-504. doi:10.1097/ede.0000000000000287

27. Angrist JD, Imbens GW, Rubin DB. Identification of Causal Effects Using Instrumental Variables. Journal of the American Statistical Association. 1996; 91(434):444. doi:10.2307/2291629

28. Bussmann H, Wester CW, Ndwapi N, et al. Five-year outcomes of initial patients treated in Botswana's National Antiretroviral Treatment Program. Aids. 2008; 22(17):2303-11. doi:10.1097/QAD.0b013e3283129db0

For peer review only - http://bmjopen.bmj.com/site/about/guidelines.xhtml

29. Belbase K, Morgan R. Food security and nutrition monitoring for drought relief management: the case of Botswana. Food Policy. 1994; 19(3):285-300. doi:10.1016/0306-9192(94)90076-0

30. De Neve J-W, Fink G. Children's education and parental old age survival – Quasi-experimental evidence on the intergenerational effects of human capital investment. Journal of Health Economics. 2018; 58:76-89. doi:10.1016/j.jhealeco.2018.01.008

31. Ma M. Does children's education matter for parents' health and cognition? Evidence from China. Journal of Health Economics. 2019; 66:222-40. doi:10.1016/j.jhealeco.2019.06.004

32. Subramanian SV, Subramanyam MA, Selvaraj S, Kawachi I. Are self-reports of health and morbidities in developing countries misleading? Evidence from India. Soc Sci Med. 2009; 68(2):260-5. doi:10.1016/j.socscimed.2008.10.017

33. He FJ, Wu Y, Feng XX, et al. School based education programme to reduce salt intake in children and their families (School-EduSalt): cluster randomised controlled trial. Bmj. 2015; 350(mar18 13):h770-h. doi:10.1136/bmj.h770

34. UK Research and Innovation. NoToNa: Tackling cardiovascular risk in the adolescent life-course through a schools' salt-reduction intervention in sub-Saharan Africa. 2020. https://gtr.ukri.org/projects?ref=MR%2FR022186%2F1. Accessed 16 Jan 2020.

35. Hughes RA, Davies NM, Davey Smith G, Tilling K. Selection Bias When Estimating Average Treatment Effects Using One-sample Instrumental Variable Analysis. Epidemiology. 2019; 30(3):350-7. doi:10.1097/ede.0000000000000972

For peer review only

For peer review only - http://bmjopen.bmj.com/site/about/guidelines.xhtml

TABLES

Table 1. Selected characteristics of estimation sample.

	Pre-reform cohorts (n = 34,202)		Post-reform cohorts (n = 55,519)	
	Census 2001	Census 2011	Census 2001	Census 2011
Children's characteristics				
Mother alive, %	88.5	77.5	90.9	81.1
Father alive, %	71.3	50.7	77.6	63.2
Age, mean (SD)	23.4 (1.7)	33.4 (1.7)	18.9 (0.8)	23.8 (3.8)
Years of schooling, mean (SD)	8.9 (3.4)	9.5 (4.0)	9.3 (3.0)	10.4 (3.3)
Has at least ten years of schooling, %	42.0	45.3	65.1	77.1
Labor force participation, %	65.3	83.6	37.3	65.0
Speaks Setswana at home, %	82.5	83.8	82.0	81.9
Christian, %	70.2	79.0	71.8	78.0
Household characteristics				
Number of family members in household, mean (SD)	5.2 (3.9)	4.5 (3.5)	6.1 (3.8)	4.8 (3.7)
Any deaths in household last year, %	5.7	2.7	6.4	2.9
Ownership of dwelling, %	63.0	57.1	74.6	62.4
Access to electricity, %	24.6	57.2	23.4	55.4
Access to piped water, %	19.5	31.0	18.5	28.2
Telephone availability, %	39.1	10.4	36.6	11.4
Cellular phone availability, %	-	94.2	-	93.9
Receives remittances, %	21.5	30.4	23.1	32.1

Sample includes survey respondents who were citizens of Botswana, born in Botswana, at least 18 years old at the time of the census, and born in or after 1975. The unit of analysis was children born either during the pre-reform period (prior to 1981) or post-reform period (in or after 1981). SD=Standard Deviation. Source: Botswana Census 2001 and 2011.

For peer review only - http://bmjopen.bmj.com/site/about/guidelines.xhtml

Table 2. First stage results: effect of the reform on children's educational attainment.

Subsample	**Daughters**	**Sons**	**Both sexes**
Dependent variable			
Child's schooling (years)	*Difference in years of schooling (95% CI)*		
Reform indicator	0.458***	0.365***	0.414***
	(0.330, 0.586)	(0.210, 0.521)	(0.314, 0.514)
Observations	47,121	42,600	89,721
R-squared	0.088	0.080	0.088
Child has ≥ 10 years of schooling (1=yes, 0=no)	*Difference in Pr(Educ ≥ 10) (95% CI)*		
Reform indicator	22.3***	15.4***	19.0***
	(20.3, 24.2)	(13.3, 17.5)	(17.5, 20.4)
Observations	47,121	42,600	89,721
R-squared	0.152	0.118	0.136

Notes: Regression results from ordinary least squares models controlling for children's single-year age indicators, a continuous trend in children's year of birth, and children's district of birth. Regressions for the subsample with both children's sexes additionally control for an indicator for children's sex and interactions of each covariate with children's sex. Our models are robust to period effects, which we controlled for implicitly by simultaneously adjusting for children's age and year of birth. Children's exposure to the reform was defined as a binary 'Reform indicator' (1=year of birth > 1980; 0=otherwise). Pr (Educ ≥ 10) was defined at the probability of a child completing at least ten years of schooling by the time of the census (1=yes, 0=no). The sample includes survey respondents who were citizens born in Botswana, at least 18 years old at the time of the census, and born in or after 1975. 95% robust confidence intervals in parentheses. Binary outcomes were multiplied by 100 to facilitate the interpretation of coefficients and standard errors on a % point scale. *** p<0.01, ** p<0.05, * p<0.1. Source: Botswana Census 2001 and 2011.

view only

Table 3. The association of children's schooling and parental survival.

Dependent variable	**Mother alive (1=yes, 0=no)**			**Father alive (1=yes, 0=no)**		
Subsample	**Daughters**	**Sons**	**Both sexes**	**Daughters**	**Sons**	**Both sexes**
			Risk difference (95% CI)			
A: OLS model						
Child's schooling (years)	0.9***	0.7***	0.8***	0.8***	0.5***	0.6***
	(0.8, 1.0)	(0.6, 0.8)	(0.7, 0.9)	(0.6, 0.9)	(0.4, 0.6)	(0.5, 0.7)
B: OLS model						
Child has ≥ 10 years of schooling (1=yes, 0=no)	4.6***	3.7***	4.1***	4.0***	1.9***	3.0***
	(3.8, 5.4)	(2.9, 4.5)	(3.6, 4.7)	(3.0, 4.9)	(0.9, 2.9)	(2.3, 3.7)
C: ITT model						
Reform indicator	-0.5	0.2	-0.2	0.4	0.7	0.5
	(-2.1, 1.0)	(-1.4, 1.8)	(-1.3, 0.9)	(-1.6, 2.5)	(-1.5, 2.8)	(-0.9, 2.0)
D: 2SLS model						
Child's schooling (years)	-1.2	0.6	-0.4	0.9	1.8	1.3
	(-4.6, 2.3)	(-3.9, 5.0)	(-3.2, 2.3)	(-3.5, 5.4)	(-4.1, 7.7)	(-2.3, 4.9)
F-statistic	49.0	21.1	65.6	49.0	21.1	65.6
Dependent variable, pre-reform (%)	83.3	83.4	83.4	61.3	62.4	61.8
Observations	47,121	42,600	89,721	47,121	42,600	89,721

Notes: **Panels A** and **B** show regression results from an ordinary least squares (OLS) model controlling for children's single-year age indicators, a continuous trend in children's year of birth, and children's district of birth. Regressions for the subsample with both children's sexes additionally control for an indicator for children's sex and interactions of each covariate with children's sex. Our models are robust to period effects, which we controlled for implicitly by simultaneously adjusting for children's age and year of birth. 'Child has ≥ 10 years of schooling' was defined at the probability of a child completing at least ten years of schooling by the time of the census (1=yes, 0=no). **Panel C** shows regression results from an intention-to-treat (ITT) OLS model in which children's exposure to the reform was defined as a binary 'Reform indicator' (1=year of birth > 1980; 0=otherwise). **Panel D** shows regression results from a two-stage least squares (2SLS) model, in which exposure to the reform was used as an instrument for children's years of schooling. Sample includes survey respondents who were citizens born in Botswana, at least 18 years old at the time of the census, and born in or after 1975. Binary outcomes were multiplied by 100 to facilitate the interpretation of coefficients and standard errors on a % point scale. 95% robust confidence intervals (CI) in parentheses. *** p<0.01, ** p<0.05, * p<0.1. Source: Botswana Census 2001 and 2011.

For peer review only - http://bmjopen.bmj.com/site/about/guidelines.xhtml

Table 4. The association of children's schooling and secondary parental outcomes.

Dependent variable / ***Sample: both sexes***	**Number of children ever born to mother**	**Age of mother's youngest child in household (years)**	**Maternal labor force participation (1=yes, 0=no)**	**Parental employment disability (1=yes, 0=no)**	**Parental blindness (1=yes, 0=no)**	**Parental lower extremity disability (1=yes, 0=no)**
A: OLS model						
Child's schooling (years)	-0.2***	0.1***	1.6***	-0.5***	-0.1**	-0.1***
	(-0.2, -0.1)	(0.0, 0.1)	(1.4, 1.7)	(-0.6, -0.4)	(-0.2, -0.0)	(-0.1, -0.0)
B: OLS model						
Child has ≥ 10 years of schooling (1=yes, 0=no)	-0.7***	0.3***	8.5***	-2.2***	-0.5**	-0.5***
	(-0.8, -0.6)	(0.1, 0.5)	(7.2, 9.7)	(-2.8, -1.6)	(-0.9, -0.0)	(-0.8, -0.2)
C: ITT model						
Reform indicator	0.0	0.3	-2.1	0.3	0.0	0.1
	(-0.1, 0.1)	(-0.1, 0.7)	(-4.5, 0.7)	(-0.7, 1.4)	(-0.8, 0.8)	(-0.4, 0.7)
D: 2SLS model						
Child's schooling (years)	0.0	0.7	-4.8	0.8	0.0	0.3
	(-0.3, 0.3)	(-0.2, 1.6)	(-10.5, 2.0)	(-1.5, 3.1)	(-1.8, 1.8)	(-0.8, 1.5)
F-statistic	29.8	29.8	29.8	33.5	33.4	33.4
Probability dependent variable, pre-reform	6.3	17.8	38.8	6.3	3.8	1.6
Observations	26,664	26,664	26,664	29,212	29,212	29,212

Notes: **Panels A** and **B** show regression results from an ordinary least squares (OLS) model controlling for children's single-year age indicators, a continuous trend in children's year of birth, children's district of birth, an indicator for children's sex, and the interactions of each covariate with children's sex. Our models are robust to period effects, which we controlled for implicitly by simultaneously adjusting for children's age and year of birth. 'Child has ≥ 10 years of schooling' was defined at the probability of a child completing at least ten years of schooling by the time of the census (1=yes, 0=no). **Panel C** shows regression results from an intention-to-treat (ITT) OLS model in which exposure to the reform was defined as a binary indicator (1=year of birth > 1980; 0=otherwise). **Panel D** shows regression results from a two-stage least squares (2SLS) model, in which exposure to the reform was used as an instrument for years of schooling. Sample includes survey respondents who were citizens born in

For peer review only - http://bmjopen.bmj.com/site/about/guidelines.xhtml

Botswana, at least 18 years old at the time of the census, born in or after 1975, and co-resided with their mother (columns 2-4) or at least one parent (columns 5-7) at the time of the census. Binary outcomes were multiplied by 100 to facilitate the interpretation of coefficients and standard errors on a % point scale. 95% robust confidence intervals in parentheses. *** $p<0.01$, ** $p<0.05$, * $p<0.1$. Source: Botswana Census 2001 and 2011.

For peer review only

FIGURES

Figure 1. Enrollment in grade 9 and 10 in Botswana by year.

Notes: Figure 1 shows total enrollment in grade 9 and 10 in Botswana by year. In January 1996, Botswana shifted grade 10 from senior secondary to junior secondary school, with the goal of increasing access. Botswana's education system is highly centralized and the reform was implemented rapidly throughout the country. More teachers were hired and classrooms were created. Moving grade 10 to junior secondary reduced travel time for students in addition to expanding the number of grade-10 seats. The reform also raised the benefits of completing grade 10 because it was now required to obtain a Junior Certificate. The result of this reform was a large increase in the number of students attending grade 10 from 1997 to 1998. Source: Botswana Education Statistics 1995 – 2000.

Figure 2. Study participant flow diagram.

Notes: Figure 2 shows the flow of participants through the Botswana Census of 2001 and 2011.

Figure 3. Parental survival and disability by children's educational attainment.

Notes: Figure 3 shows the unadjusted relationship between children's schooling and parental survival status and disability. Educational attainment was defined as total years of schooling completed by the time of the census. Strong protective effects are seen for primary school completion, with effects levelling off after junior secondary school. The sample for parental survival includes respondents who are Botswana citizens, born in Botswana, and ages at least 22 at the time of the census. The sample for parental disability includes respondents who are Botswana citizens, born in Botswana, ages at least 22, and living with at least one parent at the time of the census. Source: Botswana Census 2011.

For peer review only - http://bmjopen.bmj.com/site/about/guidelines.xhtml

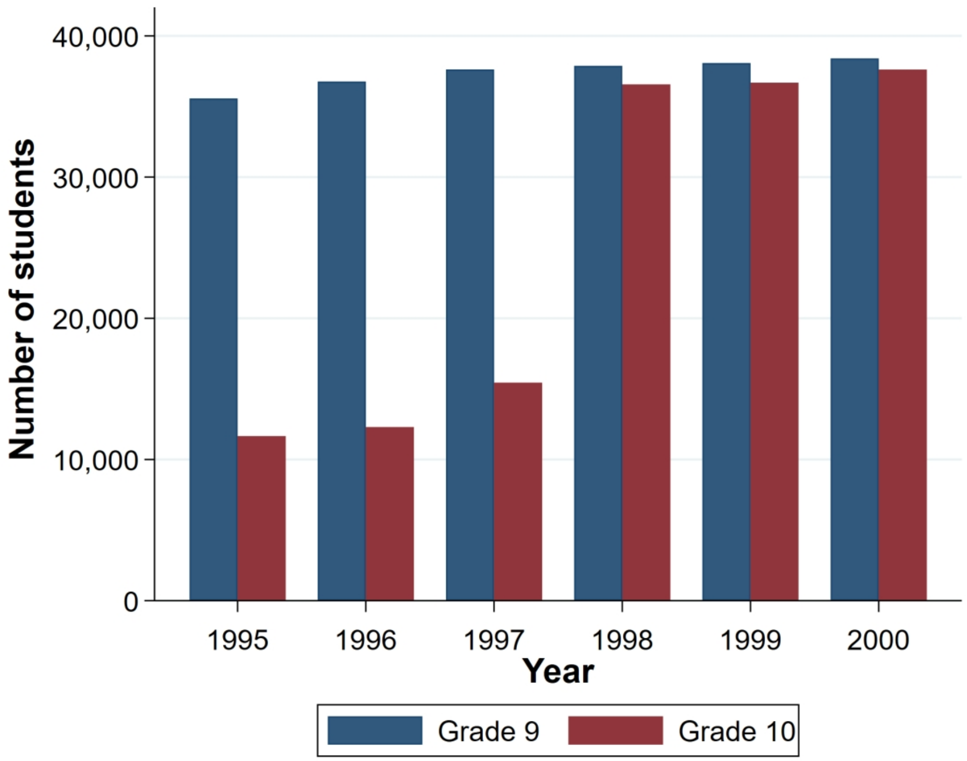

Figure 1. Enrollment in grade 9 and 10 in Botswana by year. Figure 1 shows total enrollment in grade 9 and 10 in Botswana by year. In January 1996, Botswana shifted grade 10 from senior secondary to junior secondary school, with the goal of increasing access. Botswana's education system is highly centralized and the reform was implemented rapidly throughout the country. More teachers were hired and classrooms were created. Moving grade 10 to junior secondary reduced travel time for students in addition to expanding the number of grade-10 seats. The reform also raised the benefits of completing grade 10 because it was now required to obtain a Junior Certificate. The result of this reform was a large increase in the number of students attending grade 10 from 1997 to 1998. Source: Botswana Education Statistics 1995 – 2000.

173x141mm (300 x 300 DPI)

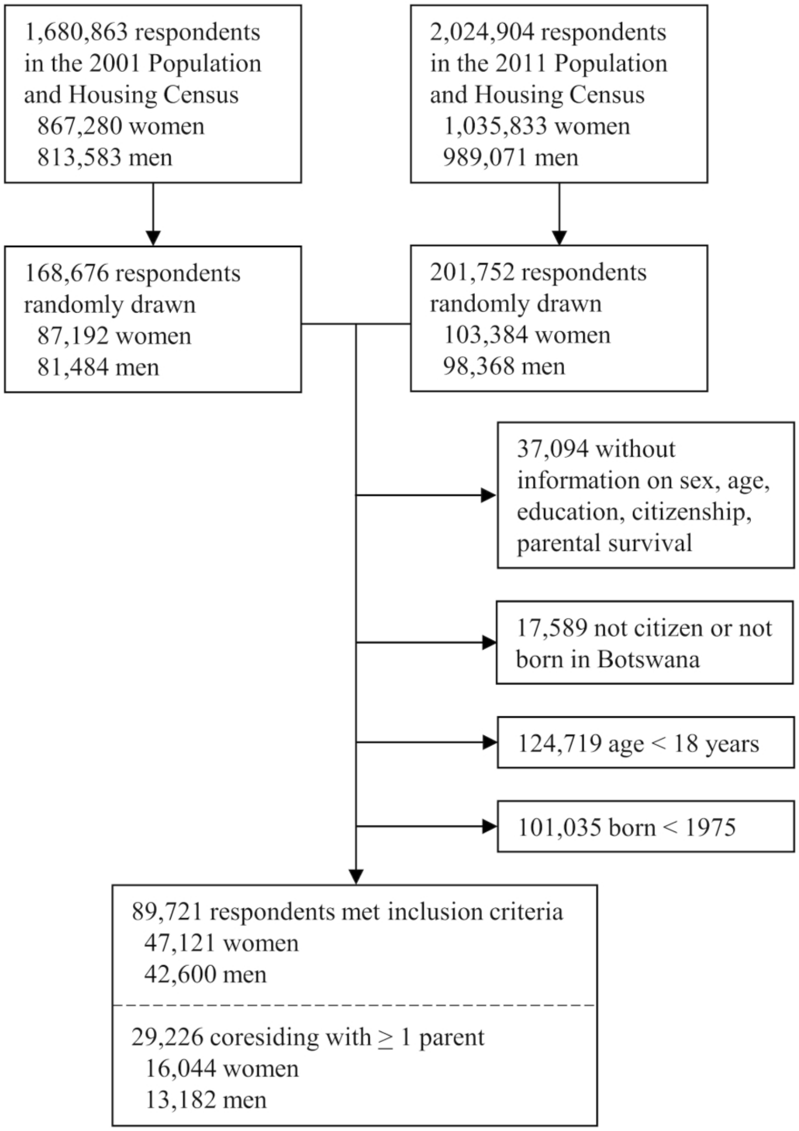

Figure 2. Study participant flow diagram. Figure 2 shows the flow of participants through the Botswana Census of 2001 and 2011.

163x222mm (300 x 300 DPI)

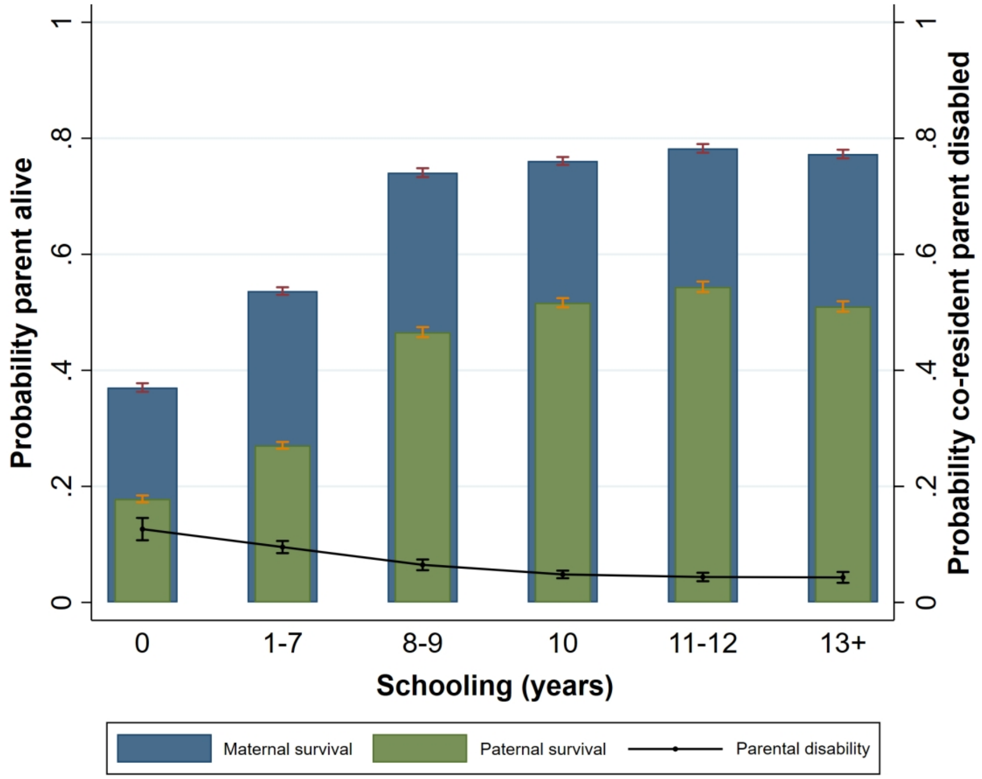

Figure 3. Parental survival and disability by children's educational attainment. Figure 3 shows the unadjusted relationship between children's schooling and parental survival status and disability. Educational attainment was defined as total years of schooling completed by the time of the census. Strong protective effects are seen for primary school completion, with effects levelling off after junior secondary school. The sample for parental survival includes respondents who are Botswana citizens, born in Botswana, and ages at least 22 at the time of the census. The sample for parental disability includes respondents who are Botswana citizens, born in Botswana, ages at least 22, and living with at least one parent at the time of the census. Source: Botswana Census 2011.

173x141mm (300 x 300 DPI)

Supplementary webappendix

- Text S1
- Figures S1-S5
- Tables S1-S7

For peer review only

For peer review only - http://bmjopen.bmj.com/site/about/guidelines.xhtml

Text S1. Study context and education policy reform

Botswana is a sparsely-populated, land-locked, middle-income country in southern Africa, with a rapidly growing economy which is highly dependent on diamond-mining. Botswana has among the world's highest HIV prevalence rates, and with the scale-up of HIV treatment, life expectancy at birth increased from 48.1 years to 61.9 years between 2001 and 2011 (World Health Organization 2020).

Botswana education system and policy reform

Botswana's public education system is based on the "7-3-2" system, including seven years of primary school, three years of junior secondary school, and two years of senior secondary school. In January 1996, the Government of Botswana shifted grade 10 from senior secondary school to junior secondary school, as part of implementing the country's Revised National Policy on Education (Government of Botswana 1994). Botswana's National Commission on Education had criticized the former "7-2-3" system for not adequately preparing students for the labor market. Botswana's public education system is strongly centralized so that the policy was implemented rapidly and universally. Moreover, the vast majority of the population attends public schools with less than 1% of secondary school students in private schools (Statistics Botswana 2013). More teachers were hired, and classrooms were created. Travel times were reduced, and the number of grade 10 seats increased dramatically since there were many more junior secondary than senior secondary schools during the pre-reform period. The schooling reform also raised the benefits of completing grade 10 because it was now required to obtain a "Junior Certificate".

The result of the reform was a very large increase in the number of students attending grade 10 from 1997 to 1998 when the first cohorts were affected (i.e., those starting junior secondary school in 1996). Previous studies have found that the reform led to a large increase in schooling attainment for affected cohorts (Borkum 2009; De Neve et al. 2015; Lindskog and Durevall 2019) (**Figure S1**). In addition to schooling attainment, the reform improved children's health outcomes (e.g., sexual and reproductive health) (Lindskog and Durevall 2019); economic outcomes (e.g., labor force participation) (Borkum 2009); skills; and health services utilization (e.g., HIV testing) (Bor and De Neve 2015). This natural experiment provides an opportunity to estimate the causal impact of schooling on parental outcomes, by comparing parents of birth cohorts exposed to the reform versus those unexposed. The 1996 policy change to an education system with three years of junior secondary school is currently still in operation.

For peer review only - http://bmjopen.bmj.com/site/about/guidelines.xhtml

Alternative hypotheses possibly affecting post-1980 birth cohorts

First, we consulted widely with scholars and government officials in Botswana to understand how the education policy reform was implemented and competing events that may have differentially affected post-1980 cohorts. Key stakeholders were identified through snowball sampling, and included staff from the Ministry of Health (n=2), Statistics Botswana (Central Statistics Botswana) (n=3), University of Botswana's Department of Economics (n=1), University of Botswana's Department of Education (n=2), the Botswana Harvard Partnership (n=2), as well as a teacher who worked in Botswana during the time of the 1996 reform (n=1). Interview questions covered the implementation of the 1996 reform; institutional and historical context; and any competing events. We asked for other social reforms or population changes that might have happened around the time and potentially have affected children born after 1980.

Second, we conducted a desk review of both published and unpublished documents. These included the Revised National Policy of Education (1994), the Revised National Population Policy (1997), the Seventh National Development Plan (1991-1997), as well as research and review articles on Botswana's education system and curriculum. In **Table S1**, we list alternative policy hypotheses with a brief description of each policy and whether or not these could be a concern for our main identification strategy. Potential concerns included, for instance, the roll-out of socio-economic reforms, vaccine or disaster relief programs, and/or HIV treatment and prevention programs if these affected specific children's birth cohorts.

For peer review only

Figure S1. Children’s schooling by year of birth in Botswana

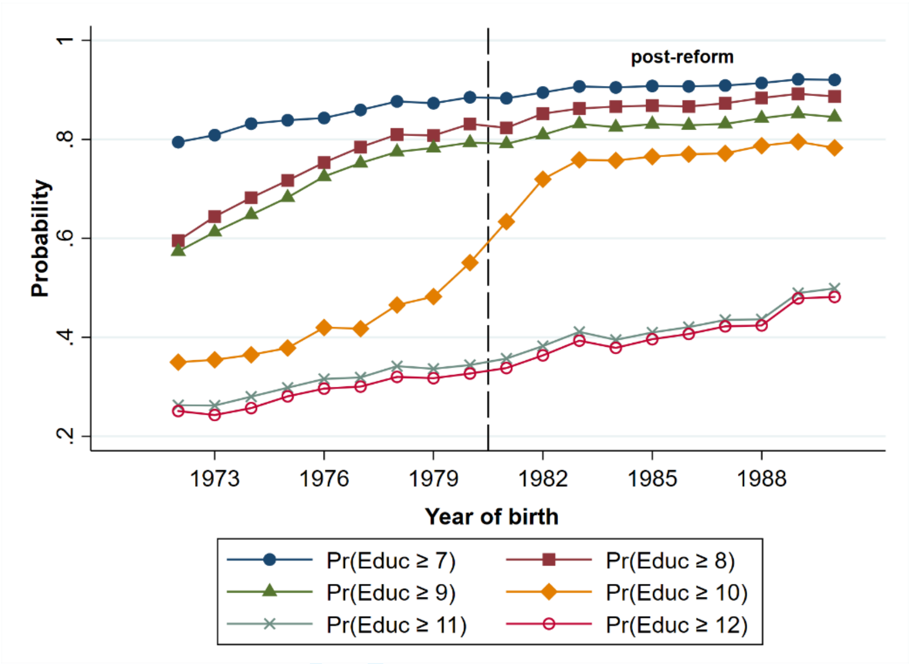

Notes: Figure replicates findings from De Neve et al., 2015 using data from the Botswana Census 2011 (*N*=108,092). The figure shows the probability that a child has attained at least X years of schooling by the time of the census. The sample includes survey respondents who were citizens of Botswana, born in Botswana and ages at least 18 years at the time of the census. Individuals born in 1981 or later (dashed line) would have entered junior secondary school in 1996 or later, and were thus classified as exposed to the education reform.

Figure S2. Assumptions underpinning causal inference

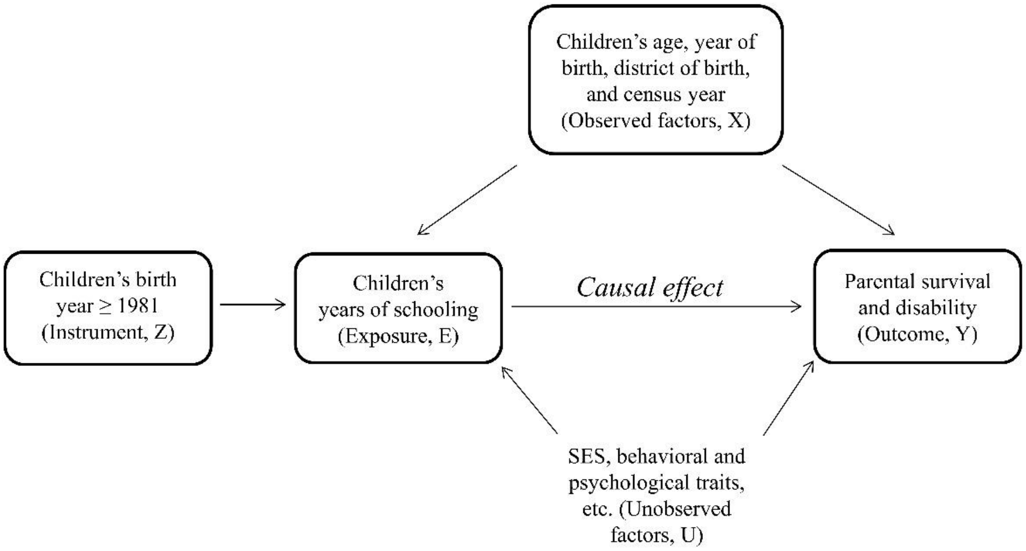

Notes: Directed acyclic graph illustrating the instrumental variable assumptions for causal interpretation. Conditional on *X*, *Z* is a valid instrument if *Z* causally affects *E*, *Z* is uncorrelated with *U*, and *Z* affects *Y* only through *E*. Under the assumption that *Z* only affects *E* in one direction, the instrumental variable estimators estimate a local average treatment effect.

For peer review only - http://bmjopen.bmj.com/site/about/guidelines.xhtml

Figure S3. Cohort sizes

(a) Census 2001

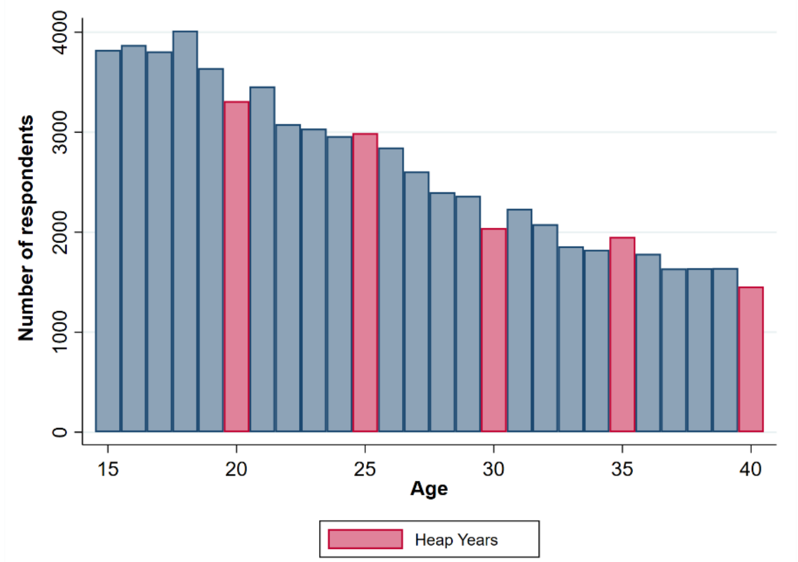

(b) Census 2011

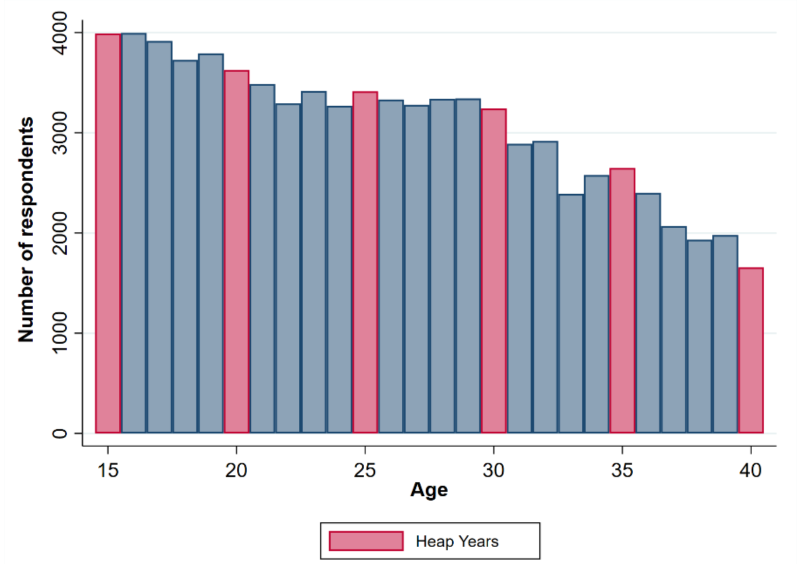

Notes: Figure shows the number of respondents by age (birth cohort), separately for each census wave. Respondents affected by the education policy reform were ages $\leq$ 20 (census 2001) or $\leq$ 30 (census 2011). Source: Botswana Census 2001 and 2011.

For peer review only - http://bmjopen.bmj.com/site/about/guidelines.xhtml

Figure S4. Parental survival and disability by children's year of birth

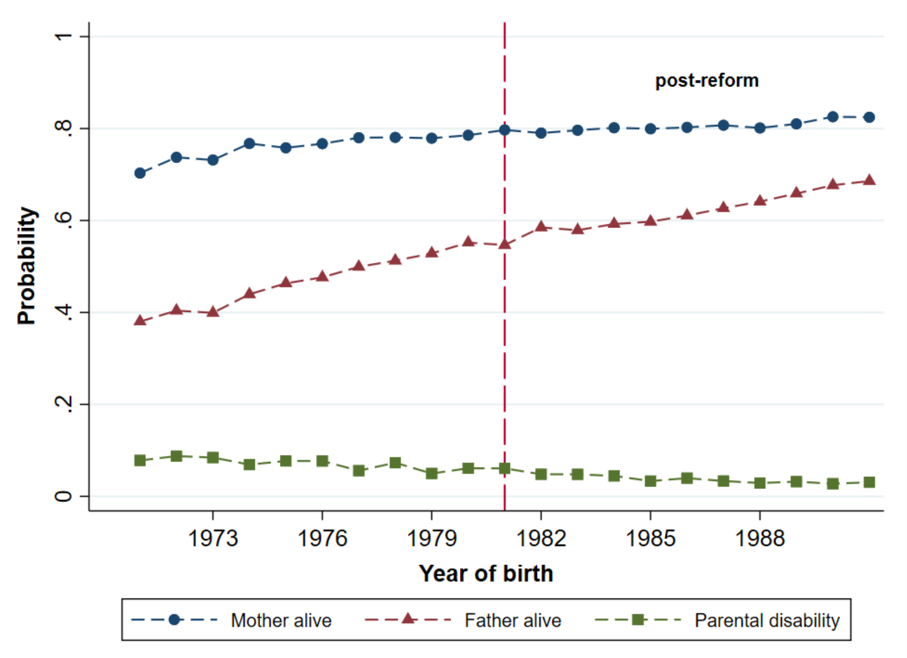

Notes: Figure shows survival of mothers (blue) and fathers (red) as well as parental disability (green) by children's birth cohort. The vertical dashed line indicates the timing of the policy reform. Parental disability was defined as any parental disability among the surviving mother and/or father. The sample for parental survival includes survey respondents who were citizens of Botswana and born in Botswana between 1971 and 1991; whereas the sample for disability additional includes only those children living with at least one parent. Source: Botswana Census 2011.

For peer review only - http://bmjopen.bmj.com/site/about/guidelines.xhtml

Figure S5. Bias component plots

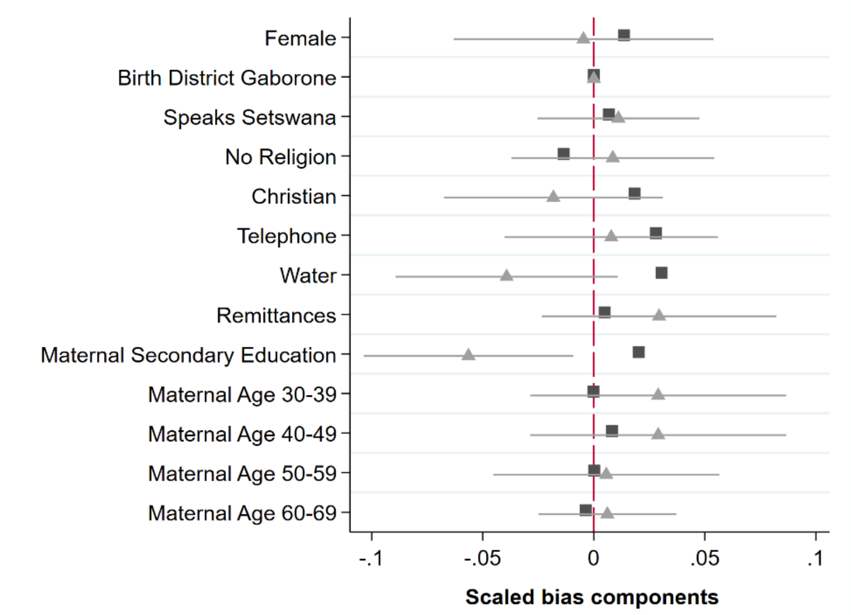

Notes: Bias component plots for the difference in children's sex, birth district, language, religion, household assets, household remittances, parental education, and parental age, by actual exposure (squares) and proposed instrument (triangles). The figures for the instrumental variable results account for the strength of the instrument as described in Jackson and Swanson (2015) and Davies (2017). All variables are binary. The horizontal lines indicate robust confidence intervals for years of schooling. Sample includes survey respondents who were citizens born in Botswana, at least 18 years old at the time of the census, born in or after 1975, and co-resided with at least one parent at the time of the census. Source: Botswana Census 2001 and 2011.

For peer review only - http://bmjopen.bmj.com/site/about/guidelines.xhtml

Table S1. Alternative Hypotheses Possibly Affecting Post-1980 Birth Cohorts.

Alternative hypothesis	Policy (year) and description	Concern or not
Curriculum changes	• Revised National Policy of Education (1994): increase the vocational orientation of academic subjects, increase the number of practical subjects, emphasize professional skills, relate the curriculum to the professional environment, and increase career guidance.	Not implemented nationally; gradual implementation over at least five years.
National ART program	• National ART Program (2002): ART became available free of charge through public health services. Aimed to deliver care with a high level of clinical monitoring and a low tolerance of adverse events, as in high-resource settings.	Rolled out later and/or did not affect specific birth cohorts.
Home based care, Prevention of Mother to Child Transmission, HIV testing policies	• Community Home Based Care (1995): ensure continuity of comprehensive care services and social support to AIDS patients.<br>• Prevention of Mother to Child Transmission (1999): available in all public health facilities.<br>• HIV testing (2000): introduction of voluntary counselling and testing centers.	Rolled out later and did not affect specific birth cohorts.
Abortion policy change	• The Penal Code Amendment Act of 11 October 1991 (1991): abortion was de-criminalized within the first 16 weeks of pregnancy under certain circumstances.	Implemented earlier and did not affect specific birth cohorts.
Family planning changes	• National Population Policy (1997): improve quality of life and standard of living of all people; through reduced population growth rate, low fertility, low morbidity and mortality, and a balanced population distribution.	Proposed almost two years after the 1996 reform (August 1997); unlikely to have affected specific birth cohorts.
Socio-economic reforms	• National Development Plan Number (1991-1997): outline short to medium-term development initiatives for Botswana	Implemented gradually over many years.
Drought relief program	• Botswana's drought relief program (1982-1990): address the loss of livestock and malnutrition, particularly among children sufficient food.	Implemented gradually over many years.

Abbreviation: ART: anti-retroviral therapy

For peer review only - http://bmjopen.bmj.com/site/about/guidelines.xhtml

Table S2. Descriptive statistics: subset of children living with at least one parent (N=29,226)

Subsample: children co-residing with ≥ 1 parent	**Pre-reform cohorts (n = 9,467)**		**Post-reform cohorts (n = 19,759)**	
	**Census 2001**	**Census 2011**	**Census 2001**	**Census 2011**
Children's characteristics				
Any parental disability, %	5.7	6.4	4.8	3.5
Age, mean (SD)	23.1 (1.7)	33.3 (1.7)	18.9 (0.8)	23.1 (3.7)
Years of schooling, mean (SD)	9.1 (3.3)	9.1 (3.6)	9.5 (2.8)	10.4 (3.1)
Has at least ten years of schooling, %	45.0	42.1	68.2	78.0
Labor force participation, %	58.2	78.2	32.4	59.8
Speaks Setswana at home, %	84.5	86.6	83.0	83.0
Christian, %	71.8	77.4	73.4	79.3
Household characteristics				
Number of family members in household, mean (SD)	8.0 (3.6)	7.7 (3.8)	7.6 (3.5)	7.1 (3.5)
Any deaths in household last year, %	7.6	5.1	6.9	4.0
Ownership of dwelling, %	90.0	94.3	87.0	86.3
Access to electricity, %	20.9	53.6	21.1	53.2
Access to piped water, %	14.6	21.1	16.1	25.1
Telephone availability, %	37.3	18.6	35.8	16.9
Cellular phone availability, %	-	95.0	-	95.1
Receives remittances, %	24.1	35.0	24.0	32.9

Sample includes survey respondents who were citizens of Botswana, born in Botswana, at least 18 years old at the time of the census, born in or after 1975, and who co-resided with at least one parent at the time of the census. The unit of analysis was children born either during the pre-reform period (prior to 1981) or post-reform period (in or after 1981). SD=Standard Deviation. Source: Botswana Census 2001 and 2011.

For peer review only

Table S3. OLS and ITT results controlling for parental socio-demographic characteristics

Dependent variable	**Parental disability (1=yes, 0=no)**		
Subsample	**Daughters**	**Sons**	**Both sexes**
	Risk difference (95% CI)		
A: OLS model			
Schooling (years)	-0.1*	-0.1	-0.1**
	(-0.2, 0.0)	(-0.2, 0.0)	(-0.2, -0.0)
B: OLS model			
≥ 10 years of schooling (1=yes,	-0.5	-1.0**	-0.7**
	(-1.3, 0.3)	(-1.9, -0.1)	(-1.3, -0.2)
C: ITT model			
Reform indicator	-0.3	-1.1	-0.7
	(-1.8, 1.1)	(-2.7, 0.5)	(-1.8, 0.4)
Additional controls			
Maternal age (years)	✓	✓	✓
Maternal schooling (years)	✓	✓	✓
Observations	15,006	12,127	27,133

Notes: Panels A and B show regression results from ‘conventional’ multivariable OLS models controlling for single-year age indicators, a continuous trend in year of birth, district of birth, as well as maternal age and maternal educational attainment (years of schooling completed). Regressions for the subsample with both children’s sexes additionally control for an indicator for children’s sex and interactions of each covariate with children’s sex. Our models are robust to period effects, which we controlled for implicitly by simultaneously adjusting for children’s age and year of birth. Panel C shows regression results from an ITT model (OLS), in which exposure to the reform was defined as a binary indicator (1=year of birth > 1980; 0=otherwise). The sample includes survey respondents who were citizens born in Botswana, at least 18 years old at the time of the census, born in or after 1975, and co-resided with at least their mother at the time of the census. Binary outcomes were multiplied by 100 to facilitate the interpretation of coefficients and standard errors on a % point scale. 95% robust confidence intervals in parentheses. *** p<0.01, ** p<0.05, * p<0.1. Source: Botswana Census 2001 and 2011.

For peer review only - http://bmjopen.bmj.com/site/about/guidelines.xhtml

Table S4. ITT results: testing the robustness of our findings to alternative specifications in children's age, YOB, additional controls for heap year, slope change in YOB, birth cohort windows, and analytical sample (outcome: maternal survival)

Dependent variable: mother alive (1=yes, 0=no)<br>***Sample: both sexes***<br>***Model: ITT***	**(1)**	**(2)**	**(3)**	**(4)**	**(5)**	**(6)**	**(7)**	**(8)**	**(9)**	**(10)**	**(11)**	**(12)**
Coefficient on endogenous variable (child)												
Reform indicator	-0.2	-0.2	0.1	0.4	0.8	0.6	-0.1	-0.2	-0.6	-0.6	0.7	-0.0
	(0.6)	(1.0)	(0.5)	(0.4)	(0.7)	(0.6)	(0.4)	(0.6)	(1.0)	(0.6)	(0.9)	(1.2)
Basic covariates (child)												
i.Age##i.Sex	✓	✓	-	-	-	-	-	✓	✓	✓	✓	✓
c.YOB##i.Sex	✓	✓	✓	✓	✓	✓	✓	✓	✓	✓	✓	✓
i.CensusYear##i.Sex	✓	✓	✓	✓	✓	✓	✓	✓	✓	✓	✓	✓
i.BirthDistrictr##i.Sex	✓	✓	✓	✓	✓	✓	✓	✓	✓	✓	✓	✓
Additional controls (child)												
c.YOB2##i.Sex	-	✓	-	-	-	-	-	-	-	-	-	-
i.ThreeYearAgeGroup##i.Sex	-	-	✓	-	-	-	-	-	-	-	-	-
c.Age##i.Sex	-	-	-	✓	✓	✓	✓	-	-	-	-	-
c.Age2##i.Sex	-	-	-	✓	✓	✓	✓	-	-	-	-	-
c.Age3##i.Sex	-	-	-	✓	-	-	-	-	-	-	-	-
c.Age4##i.Sex	-	-	-	✓	-	-	-	-	-	-	-	-
i.HeapYear##i.Sex	-	-	-	-	-	-	-	✓	-	-	-	-
c.YOB#i.Reform_Indicator, c.YOB#i.Reform_Indicator#i.Sex	-	-	-	-	-	-	-	-	✓	-	-	-
Birth cohort windows (child)												
Narrower birth cohort window, 1978 ≤ YOB ≤ 1984	-	-	-	-	✓	-	-	-	-	-	-	-
Narrower birth cohort window, 1976 ≤ YOB ≤ 1986	-	-	-	-	-	✓	-	-	-	-	-	-
Earlier birth cohorts included, 1971 ≤ YOB ≤ 1993	-	-	-	-	-	-	✓	-	-	-	-	-
Subsample, children with ≥ 9 years of schooling	-	-	-	-	-	-	-	-	-	✓	-	-
Subsample, co-resides with parent and gap ≥ 5.5 years of schooling	-	-	-	-	-	-	-	-	-	-	✓	-
Subsample, co-resides with parent and paternal age ≥ 50 years	-	-	-	-	-	-	-	-	-	-	-	✓
Observations	89,721	89,721	89,721	89,721	41,942	59,866	106,774	89,721	89,721	71,608	14,547	9,691

Notes: Robustness checks for the intention-to-treat (ITT) regression results shown in column 4 in Table 3 in the main text (maternal survival). The sample in models 1–4 and models 8–12 includes survey respondents who were citizens born in Botswana, at least 18 years old at the time of the census, and born in or after 1975. Binary outcomes were multiplied by 100 to facilitate the interpretation of coefficients and standard errors on a % point scale. Robust standard errors in parentheses. *** p<0.01, ** p<0.05, * p<0.1. Source: Botswana Census 2001 and 2011. YOB=year of birth.

For peer review only - http://bmjopen.bmj.com/site/about/guidelines.xhtml

Table S5. ITT results: testing the robustness of our findings to alternative specifications in children's age, YOB, additional controls for heap year, slope change in YOB, birth cohort windows, and analytical sample (outcome: paternal survival)

Dependent variable: father alive (1=yes, 0=no)<br>***Sample: both sexes***<br>***Model: ITT***	**(1)**	**(2)**	**(3)**	**(4)**	**(5)**	**(6)**	**(7)**	**(8)**	**(9)**	**(10)**	**(11)**	**(12)**
Coefficient on endogenous variable (child)												
Reform indicator	0.5	1.3	0.4	0.7	0.5	0.1	-0.2	-0.2	1.0	0.0	0.7	-0.5
	(0.8)	(1.4)	(0.7)	(0.6)	(0.9)	(0.7)	(0.5)	(0.6)	(1.2)	(0.8)	(0.9)	(2.1)
Basic covariates (child)												
i.Age##i.Sex	✓	✓	-	-	-	-	-	✓	✓	✓	✓	✓
c.YOB##i.Sex	✓	✓	✓	✓	✓	✓	✓	✓	✓	✓	✓	✓
i.CensusYear##i.Sex	✓	✓	✓	✓	✓	✓	✓	✓	✓	✓	✓	✓
i.BirthDistrictr##i.Sex	✓	✓	✓	✓	✓	✓	✓	✓	✓	✓	✓	✓
Additional controls (child)												
c.YOB2##i.Sex	-	✓	-	-	-	-	-	-	-	-	-	-
i.ThreeYearAgeGroup##i.Sex	-	-	✓	-	-	-	-	-	-	-	-	-
c.Age##i.Sex	-	-	-	✓	✓	✓	✓	-	-	-	-	-
c.Age2##i.Sex	-	-	-	✓	✓	✓	✓	-	-	-	-	-
c.Age3##i.Sex	-	-	-	✓	-	-	-	-	-	-	-	-
c.Age4##i.Sex	-	-	-	✓	-	-	-	-	-	-	-	-
i.HeapYear##i.Sex	-	-	-	-	-	-	-	✓	-	-	-	-
c.YOB#i.Reform_Indicator, c.YOB#i.Reform_Indicator#i.Sex	-	-	-	-	-	-	-	-	✓	-	-	-
Birth cohort windows (child)												
Narrower birth cohort window, 1978 ≤ YOB ≤ 1984	-	-	-	-	✓	-	-	-	-	-	-	-
Narrower birth cohort window, 1976 ≤ YOB ≤ 1986	-	-	-	-	-	✓	-	-	-			
Earlier birth cohorts included, 1971 ≤ YOB ≤ 1993	-	-	-	-	-	-	✓	-	-	-	-	-
Subsample, children with ≥ 9 years of schooling	-	-	-	-	-	-	-	-	-	✓	-	-
Subsample, co-resides with parent and gap ≥ 5.5 years of schooling	-	-	-	-	-	-	-	-	-	-	✓	-
Subsample, co-resides with parent and maternal age ≥ 50 years	-	-	-	-	-	-	-	-	-	-	-	✓
Observations	89,721	89,721	89,721	89,721	41,942	59,866	106,774	89,721	89,721	71,608	14,547	13,856

Notes: Robustness checks for the intention-to-treat (ITT) regression results shown in column 7 in Table 3 in the main text (paternal survival). The sample in models 1–4 and models 8–12 includes survey respondents who were citizens born in Botswana, at least 18 years old at the time of the census, and born in or after 1975. Binary outcomes were multiplied by 100 to facilitate the interpretation of coefficients and standard errors on a % point scale. Robust standard errors in parentheses. *** p<0.01, ** p<0.05, * p<0.1. Source: Botswana Census 2001 and 2011. YOB=year of birth.

Table S6. OLS and ITT results: stratified by parental age group

Dependent variable	**Parental disability (1=yes, 0=no)**		
Parental age group	**Age 50-59**	**Age 60-69**	**Age 70-79**
	Risk difference (95% CI)		
A: OLS model			
Schooling (years)	-0.0	-0.3***	-0.1
	(-0.2, 0.1)	(-0.5, -0.1)	(-0.6, 0.3)
B: OLS model			
≥ 10 years of schooling (1=yes, 0=no)	-0.5	-1.6**	-2.0
	(-1.3, 0.3)	(-3.0, -0.2)	(-4.9, 0.9)
C: ITT model			
Reform indicator	-0.6	-1.0	-2.1
	(-2.4, 1.3)	(-4.6, 2.5)	(-10.2, 6.1)
Probability dependent variable, pre-reform	5.3	8.2	13.9
Observations	12,447	6,329	2,262

Notes: Panels A and B show regression results from an OLS model controlling for single-year age indicators, a continuous trend in year of birth, district of birth, and the interaction of each covariate with children's sex. Our models are robust to period effects, which we controlled for implicitly by simultaneously adjusting for children's age and year of birth. Panel C shows regression results from an ITT model (OLS), in which exposure to the reform was defined as a binary indicator (1=year of birth > 1980; 0=otherwise). The sample includes citizens born in Botswana, at least 18 years old at the time of the census, and born in or after 1975. Binary outcomes were multiplied by 100 to facilitate the interpretation of coefficients and standard errors on a % point scale. 95% robust confidence intervals in parentheses. *** p<0.01, ** p<0.05, * p<0.1. Source: Botswana Census 2001 and 2011.

For peer review only - http://bmjopen.bmj.com/site/about/guidelines.xhtml

Table S7. OLS and ITT results: logistic regression models.

Dependent variable	**Mother alive (1=yes, 0=no)**			**Father alive (1=yes, 0=no)**		
Subsample	**Daughters**	**Sons**	**Both sexes**	**Daughters**	**Sons**	**Both sexes**
	Odds ratios (95% CI)					
A: OLS model						
Schooling (years)	1.063***	1.048***	1.055***	1.035***	1.023***	1.028***
	(1.056, 1.071)	(1.041, 1.056)	(1.050, 1.060)	(1.029, 1.041)	(1.017, 1.029)	(1.024, 1.033)
B: OLS model						
≥ 10 years of schooling (1=yes, 0=no)	1.402***	1.309***	1.356***	1.199***	1.091***	1.145***
	(1.328, 1.479)	(1.238, 1.384)	(1.304, 1.409)	(1.148, 1.252)	(1.044, 1.141)	(1.110, 1.181)
C: ITT model						
Reform indicator	0.988	1.054	1.018	1.094	1.052	1.074
	(0.776, 1.258)	(0.816, 1.362)	(0.854, 1.214)	(0.913, 1.311)	(0.869, 1.274)	(0.942, 1.225)
Observations	47,121	42,600	89,721	47,121	42,600	89,721

Notes: Logistic regression models controlling for single-year children's age indicators, a continuous trend in children's year of birth and year of birth squared, and children's district of birth. Regressions for the subsample with both sexes additionally control for an indicator for children's sex and interactions of each covariate with children's sex. Our models are robust to period effects, which we controlled for implicitly by simultaneously adjusting for children's age and year of birth. Panel C shows regression results from an intention-to-treat (ITT) model in which exposure to the reform was defined as a binary indicator (1=year of birth > 1980; 0=otherwise). The sample includes survey respondents who were citizens born in Botswana, at least 18 years old at the time of the census, and born in or after 1975. Robust 95% confidence intervals in parentheses. *** p<0.01, ** p<0.05, * p<0.1. Source: Botswana Census 2001 and 2011.

For peer review only - http://bmjopen.bmj.com/site/about/guidelines.xhtml

References for supplementary webappendix

1. Bor, J.and J.-W. De Neve (2015). "A Social Vaccine? HIV Infection, Fertility, and the Non-Pecuniary Returns to Secondary Schooling in Botswana." Population Association of America Annual Meeting. San Diego [Available at: https://paa2015.princeton.edu/abstracts/151006].
2. Borkum, E. (2009). "Grade structure, educational attainment and labor market outcomes: Evidence from Botswana" (Job Market Paper). Columbia University.
3. Government of Botswana. (1991). National Development Plan - 7. Gaborone, Botswana: Botswana Government Printer.
4. Government of Botswana. (1994). The revised national policy on education. Gaborone, Botswana: Botswana Government Printer.
5. Government of Botswana. (1997). National Population Policy. Gaborone, Botswana: Botswana Government Printer.
6. De Neve J-W, Fink G, Subramanian SV, Moyo S, & Bor J (2015) Length of secondary schooling and risk of HIV infection in Botswana: evidence from a natural experiment. *The Lancet Global Health* 3(8):e470-e477.
7. Statistics Botswana (2013). "Education Report Draft from 2009/10 Botswana Core Welfare Indicators Survey." Gaborone, Botswana: Government of Botswana [Available at: http://www.statsbots.org.bw/].
8. World Health Organization (2020). "Global Health Observatory data repository." World Health Organization. Geneva, Switzerland [Available at: https://www.who.int/data/gho].

For peer review only

For peer review only - http://bmjopen.bmj.com/site/about/guidelines.xhtml

STROBE Statement—Checklist of items that should be included in reports of ***cross-sectional studies***

	Item No	Recommendation
**Title and abstract**	1	*(a)* Indicate the study's design with a commonly used term in the title or the abstract<br>Title page
		(b) Provide in the abstract an informative and balanced summary of what was done and what was found<br>Page 3
**Introduction**		
Background/rationale	2	Explain the scientific background and rationale for the investigation being reported<br>Pages 5-6
Objectives	3	State specific objectives, including any prespecified hypotheses<br>Pages 5-6
**Methods**		
Study design	4	Present key elements of study design early in the paper<br>Pages 7-14
Setting	5	Describe the setting, locations, and relevant dates, including periods of recruitment, exposure, follow-up, and data collection<br>Pages 7-8; Text S1 in the Supplementary webappendix
Participants	6	*(a)* Give the eligibility criteria, and the sources and methods of selection of participants<br>Pages 7-8
Variables	7	Clearly define all outcomes, exposures, predictors, potential confounders, and effect modifiers. Give diagnostic criteria, if applicable<br>Pages 8-14
Data sources/ measurement	8*	For each variable of interest, give sources of data and details of methods of assessment (measurement). Describe comparability of assessment methods if there is more than one group<br>Pages 7-9
Bias	9	Describe any efforts to address potential sources of bias<br>Pages 12-14, Pages 19-20
Study size	10	Explain how the study size was arrived at<br>See Figure 2 for a study participant flow diagram
Quantitative variables	11	Explain how quantitative variables were handled in the analyses. If applicable, describe which groupings were chosen and why<br>Pages 8-9
Statistical methods	12	*(a)* Describe all statistical methods, including those used to control for confounding<br>Pages 10-14
		(b) Describe any methods used to examine subgroups and interactions<br>Pages 10-14
		(c) Explain how missing data were addressed<br>Page 14
		(d) If applicable, describe analytical methods taking account of sampling strategy
		(*e*) Describe any sensitivity analyses Pages 13-14
**Results**		
Participants	13*	(a) Report numbers of individuals at each stage of study—eg numbers potentially

For peer review only

		eligible, examined for eligibility, confirmed eligible, included in the study, completing follow-up, and analysed<br>See Figure 2 for a study participant flow diagram
		(b) Give reasons for non-participation at each stage<br>See Figure 2 for a study participant flow diagram
		(c) Consider use of a flow diagram<br>Figure 2
Descriptive data	14*	(a) Give characteristics of study participants (eg demographic, clinical, social) and information on exposures and potential confounders<br>Page 15
		(b) Indicate number of participants with missing data for each variable of interest
Outcome data	15*	Report numbers of outcome events or summary measures<br>Page 15
Main results	16	*(a)* Give unadjusted estimates and, if applicable, confounder-adjusted estimates and their precision (eg, 95% confidence interval). Make clear which confounders were adjusted for and why they were included<br>Tables 2 and 3
		(b) Report category boundaries when continuous variables were categorized<br>Tables 1 and 2
		(c) If relevant, consider translating estimates of relative risk into absolute risk for a meaningful time period Tables 2 and 3 report absolute Risk Differences (95% CIs).
Other analyses	17	Report other analyses done—eg analyses of subgroups and interactions, and sensitivity analyses Pages 17-18
**Discussion**		
Key results	18	Summarise key results with reference to study objectives<br>Page 18
Limitations	19	Discuss limitations of the study, taking into account sources of potential bias or imprecision. Discuss both direction and magnitude of any potential bias<br>Pages 19-20
Interpretation	20	Give a cautious overall interpretation of results considering objectives, limitations, multiplicity of analyses, results from similar studies, and other relevant evidence<br>Pages 18-21
Generalisability	21	Discuss the generalisability (external validity) of the study results<br>Pages 19-20
**Other information**		
Funding	22	Give the source of funding and the role of the funders for the present study and, if applicable, for the original study on which the present article is based<br>Title page

*Give information separately for exposed and unexposed groups.

Note: An Explanation and Elaboration article discusses each checklist item and gives methodological background and published examples of transparent reporting. The STROBE checklist is best used in conjunction with this article (freely available on the Web sites of PLoS Medicine at http://www.plosmedicine.org/, Annals of Internal Medicine at http://www.annals.org/, and Epidemiology at http://www.epidem.com/). Information on the STROBE Initiative is available at www.strobe-statement.org.

For peer review only
